# A Review of Trimming in Isogeometric Analysis: Challenges, Data Exchange and Simulation Aspects

**DOI:** 10.1007/s11831-017-9220-9

**Published:** 2017-06-02

**Authors:** Benjamin Marussig, Thomas J. R. Hughes

**Affiliations:** Institute for Computational Engineering and Sciences, The University of Texas at Austin, 201 East 24th St, Stop C0200, Austin, TX 78712 USA

## Abstract

We review the treatment of trimmed geometries in the context of design, data exchange, and computational simulation. Such models are omnipresent in current engineering modeling and play a key role for the integration of design and analysis. The problems induced by trimming are often underestimated due to the conceptional simplicity of the procedure. In this work, several challenges and pitfalls are described.

## Introduction

Trimming is much more complicated than most people think. It is one of the most fundamental procedures in Computer Aided Geometric Design (CAGD) that allows the construction of complex geometries. Unfortunately, it is also the source of one of the most serious impediments to interoperability between Computer Aided Design (CAD) systems and downstream applications like numerical simulation [268]. This work aims to increase awareness of this issue by providing a broad overview of trimmed geometries, addressing design, data exchange, and analysis aspects.

Once upon a time, the original vision of CAD was the holistic treatment of the engineering design process [247]. However, it has emerged as an autonomous discipline which seeks to optimize the modeling and visualization of geometric objects. On the other hand, computational analysis has focused on the problem-solving part of engineering. Thus, the main attention has been drawn to the development of mathematical models governing physical phenomena as well as the reliability and efficiency of their numerical treatment. Still, design models are usually the starting point of the analysis process in order to define the domain of interest. In current engineering design, however, they are subsequently approximated by finite element meshes for computation. Since this is a fundamental step in conventional simulations, there is a substantial body of literature on meshing, see e.g., [28, 94, 98, 281, 322] and the references cited therein. Finite element analysis (FEA) was a widely used commercially available procedure in engineering prior to the advent of commercial CAD. Nevertheless, FEA finds itself separated from design by its own representation of geometrical objects, which is different from CAD. The given situation has contributed to a loss of communication between these fields, both of which are essential in the process of addressing practical engineering problems.

Isogeometric analysis [66, 140] provides an alternative to the conventional analysis methodology that converts CAD models for use in FEA. The key idea is to perform numerical simulations based on CAGD technologies. Besides the fact that this synthesis offers several computational benefits, such as high continuity [67, 68, 187], the long term goal of isogeometric analysis is to enhance the overall engineering product development process by closing the gap between design and analysis. An invaluable byproduct of this effort is the initiation of a dialog between these two communities which had drifted apart.

**Fig. 1:**
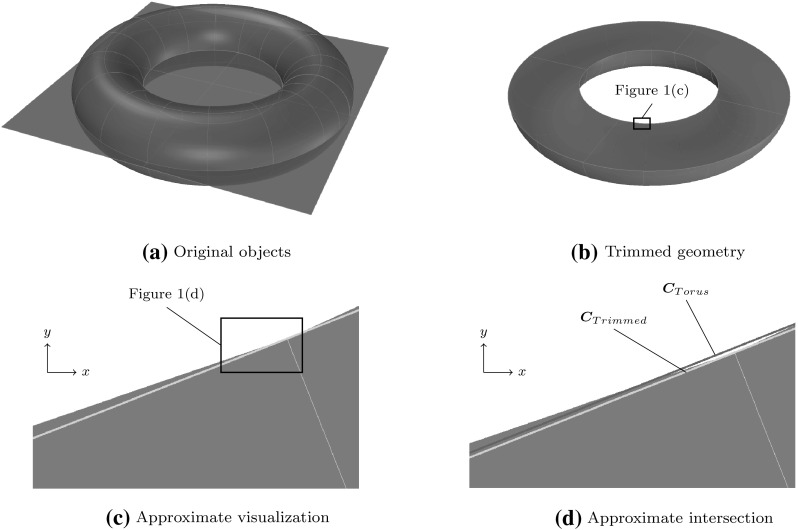
Model of a half of a torus: **a** initial non-trimmed torus and surface defined in the *xy*-plane, **b** resulting trimmed object, **c** closeup showing the deviation from the visualization mesh (*blue background*) and the computed intersection curve ${\varvec{C}}_{Trimmed}$ (*yellow*) of the objects, and **d** closeup illustrating the difference of the original inner *circle* of the torus ${\varvec{C}}_{Torus}$ (*red*) to the intersection curve computed. The images **c** and **d** are captured in top view. (Color figure online)

Gaining insight into each others’ fields is an essential component to tackle the problem of interoperability between analysis and design representations. It is easily overlooked that each discipline has its open challenges and limitations. Due to the capability of current CAD systems it may seem that design models are ideal creations, but this notion is simply not true. There are several open issues that need to be solved. Compact overviews are given in the independent compilations of Kasik et al. [151] and Piegl [228]. In these papers, robustness and interoperability issues are identified as crucial CAD problems. Although trimmed geometries are not explicitly mentioned in these papers, they play a central role in both cases.

The most common description of CAGD models is the boundary representation (B-Rep) where an object is represented by its boundary surfaces rather than a volume discretization. These surfaces are usually constructed independently from each other and often only certain regions of a surface are supposed to be part of the actual object. Trimming allows a modeler to cut away the superfluous surface areas. To be precise, the visualization of the surfaces is adapted while their parameterization and mathematical description remain unchanged. This procedure is very convenient and inevitable in many operations such as surface-to-surface intersection. However, the main problem is that trimming *cannot* practically be performed exactly within CAGD applications. Thus, the final object possesses small gaps and overlaps between its surfaces. Figure 1 illustrates some inaccuracies of a model defined by a torus intersected by a plane. Note that the discrepancy between the computed intersection ${\varvec{C}}_{Trimmed}$ and the related exact solution ${\varvec{C}}_{Torus}$ is scarcely visible. The imperfections of trimmed geometries are usually very well hidden from the user, but they surface as soon as a design model is applied to downstream applications. To use the words of Piegl [228]:

While one can cheat the eye in computer graphics and animation, the milling machine is not as forgiving.

Numerical simulation of practical trimmed models is more than the analysis of a specific type of a CAGD representation. It rather addresses the core issue of the interoperability between design and analysis, namely the appropriate treatment of the deficiencies of design models. To be clear, this problem is not restricted to isogeometric analysis, but manifests itself as complications during the meshing process in the case of conventional analysis methodology. In fact, geometry repair and corrections of design models are mandatory tasks, before actual mesh generation can be applied [86, 114]. Isogeometric analysis of trimmed geometries tackles these issues directly at the source, i.e., the design model. Thus, many pitfalls that may occur in a meshing process can be circumvented [61].

It is important to note that the CAD community is also influenced by the ongoing dialog. An increasing number of researchers propose new modeling concepts that take the needs of downstream applications into account, see e.g. [61, 203, 247]. We believe that the aligned efforts of both communities are the keys to unite design and analysis, resulting in a holistic treatment of the engineering design process.

This paper intends to encourage the interaction of these fields by providing an overview of various aspects related to trimmed geometries. Section 2 begins by reviewing some basic concepts frequently used in CAGD. It is focused on non-uniform rational B-spline (NURBS) based B-Rep models since they are the most popular representation in engineering design. Based on this, Sect. 3 addresses the role of trimming in the context of design. A critical assessment of exchanging data between different software packages is provided in Sect. 4. Finally, various strategies to deal with trimmed geometries in an isogeometric analysis process are outlined in Sect. 5. Each of these three review sections closes with a brief summary of the main points and their discussion. Section 6 moves on to focus on a particular aspect, namely the stabilization of a trimmed basis. In the concluding section, the main findings are summarized and some open research questions are listed.

## CAGD Fundamentals

B-splines and their rational counterpart NURBS provide the basis for the geometric modeling of most engineering models. This section gives a brief overview of this CAGD technology focusing on aspects which are crucial for the subsequent discussion. For further information related to spline theory the interested reader is referred to [34, 62, 83]. Detailed descriptions of efficient algorithms can be found in [230]. In the present paper, the terms B-spline and NURBS are used to refer to basis functions. The geometric objects described using these functions, i.e., curves and surfaces, may be generally denoted as patches.

### Basis Functions

B-splines ${B_{i,p}}$ consist of piecewise polynomial segments which are connected by a certain smoothness. They are defined recursively for a fixed polynomial degree $p$ by a strictly convex combination of B-splines of the previous degree, $p-1,$ given by

**Fig. 2:**
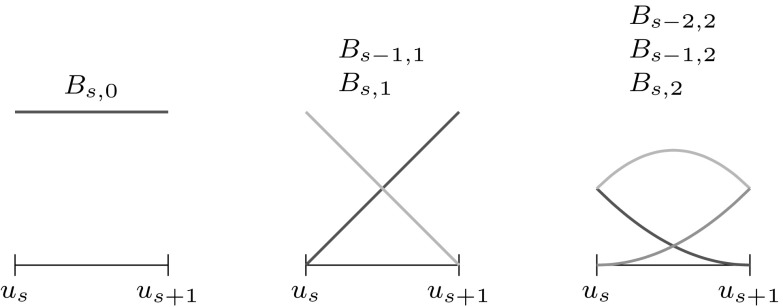
Non-vanishing B-splines ${B_{i,p}}$ of knot span $s$ for different degrees $p= \{0,\,1,\,2\}$ which are based on a knot vector with equally spaced knots

1 $$\begin{aligned}{B_{i,p}}(u) &= \frac{u-{u_{i}}}{{u_{i+p}}-{u_{i}}} \, {B_{i,p-1}}(u) \nonumber \\&\quad + \frac{{u_{i+p+1}}-u}{{u_{i+p+1}}-{u_{i+1}}} \, {B_{i+1,p-1}}(u) ,\end{aligned}$$

with

2 $$\begin{aligned}{B_{i,0}}(u)& = \left\{\begin{array}{ll} 1 & {if}\,{u_{i}}\leq u< {u_{i+1}},\\ 0 & {otherwise.} \end{array}\right. \end{aligned}$$

**Fig. 3:**
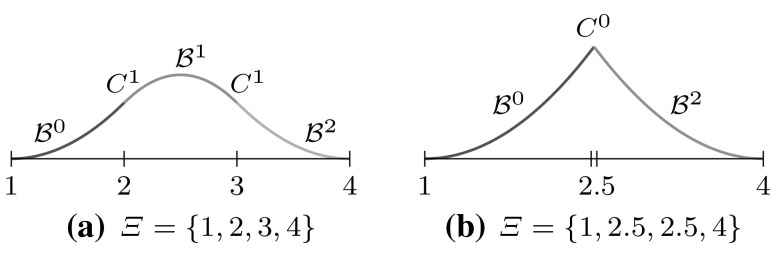
Polynomial segments ${{\mathcal {B}}^{s}}$ of a quadratic B-spline due to different knot vectors $\varXi .$ Note the different continuity *C* between the segments based on the knot multiplicity

The essential element for this construction is the *knot vector* $\varXi$ characterized as a non-decreasing sequence of coordinates ${u_{i}} \leqslant {u_{i+1}}$. The parameters ${u_{i}}$ are termed *knots* and the half-open interval $[{u_{i}},\, {u_{i+1}})$ is called $i$th *knot span*. Each knot span has $p+1$ non-vanishing B-splines as illustrated in Fig. 2. Each basis function is entirely defined by $p+ 2$ knots and its support, ${{\text{supp}}}{ \{{B_{i,p}} \} }=\{ {u_{i}}, \ldots , {u_{i+p+1}} \},$ is local. Within each non-zero knot span $s,\, {u_{s}} < {u_{s+1}},$ of its support, ${B_{i,p}}$ is described by a polynomial segment ${{\mathcal {B}}^{s}_{i}}.$ Each knot value indicates a location within the parameter space which is not ${C^{\infty} }$-continuous, i.e., where two adjacent ${{\mathcal {B}}^{s}_{i}}$ join. Successive knots may share the same value, which is indicated by the knot multiplicity $m,$ i.e., ${u_{i}} = {u_{i+1}} = \cdots = {u_{i+m-1}}.$ In general, the continuity between adjacent segments is ${C^{p-m}}.$ This control of continuity is demonstrated for a quadratic B-spline in Fig. 3. If the multiplicity of the first and last knot is equal to $p+1,$ the knot vector is denoted an *open* knot vector. The knot sequence

3 $$\begin{aligned} \varXi = \left\{ {u_{0}} = \cdots = {u_{p}},\, {u_{p+1}} = \cdots = {u_{2p+1}} \right\} , \end{aligned}$$

is a special from of such a knot vector since it yields the classical $p$th-degree Bernstein polynomials. To be precise, Bernstein polynomials are usually defined over the interval $[0,\,1].$ If necessary, the restriction to this interval can be easily accomplished by a coordinate transformation.

As a whole, B-splines based on a common knot vector $\varXi$ form a *partition of unity*, i.e.,

4 $$\begin{aligned} {\sum _{i=0}^{I-1}} {B_{i,p}}(u) = 1,\quad u\in \left[ {u_{0}},\,{u_{I+p}} \right] , \end{aligned}$$

and they are *linearly independent*, i.e.,

5 $$\begin{aligned} {\sum _{i=0}^{I-1}} {B_{i,p}}(u) {c_{i}} = 0, \end{aligned}$$

is satisfied if and only if $c_{i} = 0,~i= 0,\ldots ,I-1.$ Due to the latter property, every piecewise polynomial ${f_{p,\varXi} }$ of degree $p$ over a knot sequence $\varXi$ can be uniquely described by a linear combination of the corresponding ${B_{i,p}}.$ Hence, they form a *basis* of the space ${{\mathbb {S}}_{p,\varXi }}$ collecting all such functions

6 $$\begin{aligned} {{\mathbb {S}}_{p,\varXi }} ={ \sum _{i=0}^{I-1}} {B_{i,p}} {c_{i}}, \quad {c_{i}} \in {\mathbb {R}}. \end{aligned}$$

**Fig. 4:**
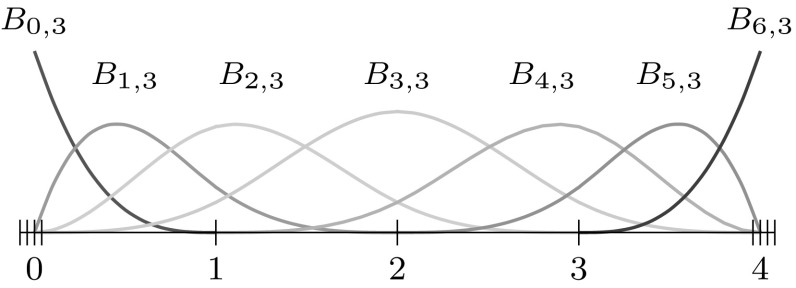
B-spline basis specified by an open knot vector, i.e., $\varXi =\{0,\,0,\,0,\,0,\,1,\,2,\,3,\,4,\,4,\,4,\,4\}$

An example of a cubic B-spline basis defined by an open knot vector is shown in Fig. 4.

The first derivative of B-splines are computed by a linear combination of B-splines of the previous degree

7 $$\begin{aligned} {B^{\prime }_{i,p}}(u)&= \frac{p}{{u_{i+p}}-{u_{i}}} {B_{i,p-1}}(u) \nonumber \\&\quad - \frac{p}{{u_{i+p+1}}-{u_{i+1}}} {B_{i+1,p-1}}(u). \end{aligned}$$

For the computation of the *k*th derivative, this is generalized to

8 $$\begin{aligned} {B^{(k)}_{i,p}}(u)&= \frac{p!}{(p- k )! } {\sum _{\ell =0}^{k}} {a_{k,\ell} } {B_{i+\ell ,p-k}}(u), \end{aligned}$$

with

$$\begin{aligned}&{a_{0,0}} = 1, \nonumber \\&{a_{k,0}} = \frac{{a_{k-1,0}}}{{u_{i+p-k+1}}-{u_{i}}}, \nonumber \\&{a_{k,\ell }} = \frac{{a_{k-1,\ell} } - {a_{k-1,\ell -1}}}{u_{i+p+\ell -k+1}-u_{i+\ell }} \quad \ell = 1,\ldots ,k-1, \nonumber \\&{a_{k,k}} = \frac{-{a_{k-1,k-1}}}{{u_{i+p+1}}-{u_{i+k}}} \nonumber . \end{aligned}$$

#### *Remark 1*

The knot differences of the denominators involved in the recursive formulae (1), (7) and (8) can become zero. In such a case the quotient is defined to be zero.

### Curves

B-spline curves of degree $p$ are defined by basis functions ${B_{i,p}}$ due to a knot vector $\varXi$ with corresponding coefficients in model space^1^ ${{\varvec{c}}_{i}}$ which denote *control points*. The geometrical mapping ${\mathcal {X}}$ from parameter space to model space is given by

9 $$\begin{aligned} {\mathcal {X}}(u)&:= {\varvec{C}}(u) = {\sum _{i=0}^{I-1}} {B_{i,p}}(u) {{\varvec{c}}_{i}}, \end{aligned}$$

with $I$ representing the total number of basis functions. The derivative is

10 $$\begin{aligned} {{\mathbf {J}}_{{\mathcal {X}}}} (u)&:= {\sum _{i=0}^{I-1}} {{B^{\prime}}_{i,p}}(u) {{\varvec{c}}_{i}}. \end{aligned}$$

**Fig. 5:**
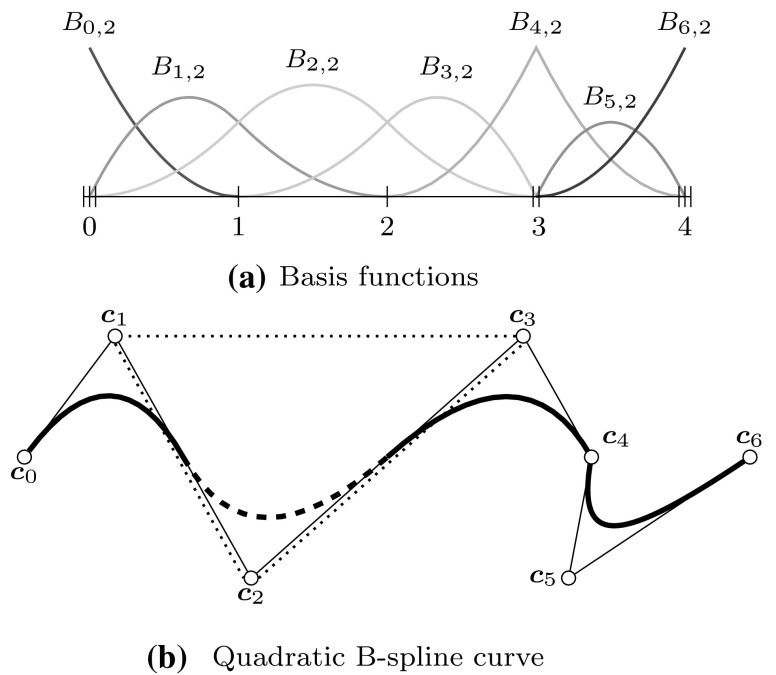
Example of a B-spline curve: **a** B-splines based on $\varXi =\{0,\,0,\,0,\,1,\,2,\,3,\,3,\,4,\,4,\,4\}$ and **b** a corresponding piecewise polynomial curve. In **b**, *circles* denote control points and the *dotted lines* indicate the convex hull of the *dashed curve* segment $u\in [1,\,2)$

In general, control points ${{\varvec{c}}_{i}}$ are not interpolatory, i.e., they do not lie on the curve. The connection of ${{\varvec{c}}_{i}}$ by straight lines is called the *control polygon* and it provides an approximation of the actual curve. An important property of a B-spline curve is that it is contained within the *convex hull* of its control polygon. In particular, a polynomial segment related to a non-zero knot span $s,$ i.e., $u\in [{u_{s}},\,{u_{s+1}}),$ is in the convex hull of the control points ${{\varvec{c}}_{s-p}},\ldots ,{{\varvec{c}}_{s}}.$ The continuity of the whole piecewise polynomial curve ${\varvec{C}}(u)$ is inherited from its underlying basis functions, i.e., the continuity at knots is determined by the knot multiplicity, and the position of its control points. These relationships are illustrated in Fig. 5. Note that the interpolatory B-spline ${B_{4,2}}$ of Fig. 5a corresponds to the kink at ${{\varvec{c}}_{4}}$ in Fig. 5b and that the second polynomial segment lies within the convex hull of ${{\varvec{c}}_{1}}$ to ${{\varvec{c}}_{3}}.$ If the curve consist of a single polynomial segment, i.e., the associated $\varXi$ is of form (3), the curve is referred to as *Bézier* curve. A polynomial segment of a B-spline curve is termed a Bézier segment, if it can be represented by a Bézier curve. In Fig. 5b, this is the case for the segment $u\in [3,\,4]$ defined by the control points ${{\varvec{c}}_{4}}$ to ${{\varvec{c}}_{6}}.$

B-spline curves can be generalized to represent rational functions such as conic sections. For this purpose, *weights* ${w_{i}}$ are associated with the control points such that

11 $$\begin{aligned} {{\varvec{c}}^{h}_{i}}&=\left( {w_{i}} {{\varvec{c}}_{i}},\, {w_{i}}\right) ^{\intercal } = \left( {{\varvec{c}}^{w}_{i}},\, {w_{i}}\right) ^{\intercal }\in {{\mathbb {R}}^{d+1}}, \end{aligned}$$

where $d$ denotes the spatial dimension of the model space. The *homogeneous coordinates* ${{\varvec{c}}^h_{i}}$ specify a B-spline curve ${{\varvec{C}}^{h}}(u)$ in a projective space ${{\mathbb {R}}^{d+1}}.$ In order to obtain a curve in ${{\mathbb {R}}^{d}},$ the geometrical mapping (9) is extended by a perspective mapping ${\mathcal {P}}$ with the center at the origin of ${{\mathbb {R}}^{d+1}}.$ This projection is given by

12 $$\begin{aligned} {\varvec{C}}(u)&= {\mathcal {P}}({{\varvec{C}}^{h}}(u)) = \frac{{{\varvec{C}}^{w}}(u) }{w(u)}, \end{aligned}$$

where ${{\varvec{C}}^{w}} = ({{\varvec{C}}^{h}_{1}},\ldots ,{{\varvec{C}}^{h}_{d}})^{\intercal }$ are the homogeneous vector components of the curve and the weighting function is determined by

13 $$\begin{aligned} w(u) = {\sum _{i=0}^{I-1}} {B_{i,p}} (u) {w_{i}}. \end{aligned}$$

**Fig. 6:**
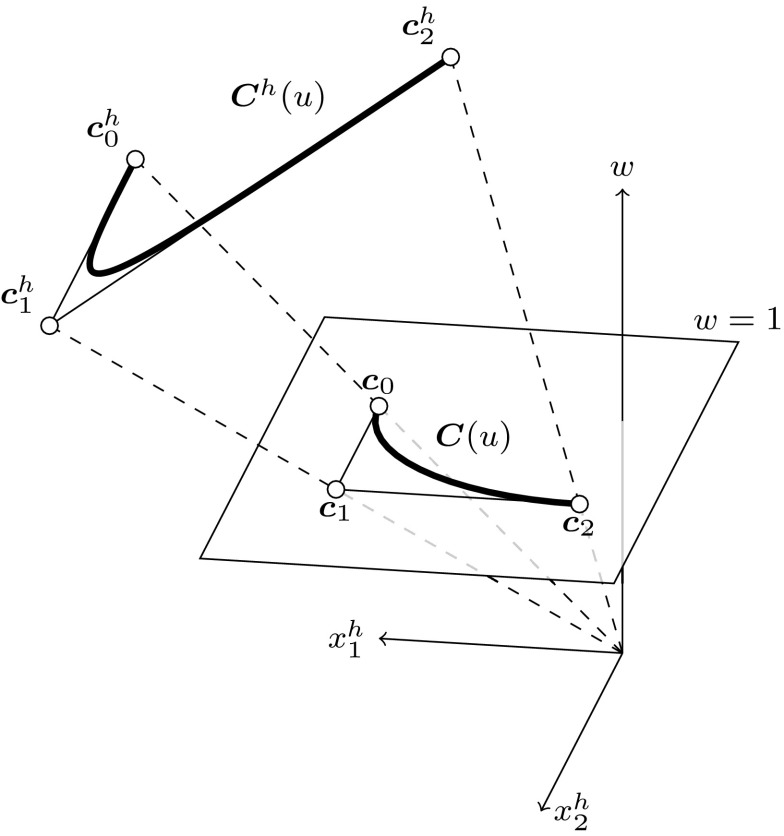
Perspective mapping ${\mathcal {P}}$ of a quadratic B-spline curve ${{\varvec{C}}^{h}}(u)$ in homogeneous form ${{\mathbb {R}}^{3}}$ to a circular arc ${\varvec{C}}(u)$ in model space ${{\mathbb {R}}^{2}}.$ The mapping is indicated by *dashed lines*

The application of Eq. (12) is illustrated in Fig. 6. The projection ${\varvec{C}}(u)$ is denoted as a non-uniform rational B-spline (NURBS) curve. The term rational indicates that the resulting curves are piecewise rational polynomials, whereas the term non-uniform emphasizes that the knot values can be distributed arbitrarily.

The derivative of the NURBS geometrical mapping is defined by

14 $$\begin{aligned} {{\mathbf {J}}_{{\mathcal {X}}}} {(u)}&:= \frac{ w(u)\frac{\partial {{{\varvec{C}}}^{w}}{(u)} }{ \partial {u}} - \frac{\partial {w(u)} }{ \partial {u}} {{\varvec{C}}^{w}}(u) }{(w(u))^{2}}, \end{aligned}$$

with

15 $$\begin{aligned} \frac{\partial {w(u)} }{ \partial {u}}&= {\sum _{i=0}^{I-1}} {B^{\prime }_{i,p}} (u) {w_{i}}, \end{aligned}$$

16 $$\begin{aligned} \frac{\partial {{{\varvec{C}}}^{w}}{(u)} }{ \partial {u}}&= {\sum _{i=0}^{I-1}} {B^{\prime }_{i,p}} (u) {{\varvec{c}}^{w}_{i}}. \end{aligned}$$

Another way to represent NURBS curves is

17 $$\begin{aligned} {\varvec{C}}{(u)} = {\sum _{i=0}^{I-1}} {R_{i,p}}{(u)} {{\varvec{c}}_{i}}, \end{aligned}$$

with

18 $$\begin{aligned} {R_{i,p}}{(u)} = \frac{ {w_{i}} {B_{i,p}}{(u)}}{ w{(u)} }. \end{aligned}$$

The weighting function $w{(u)}$ is the same as in Eq. (13) and ${R_{i,p}}$ denotes a NURBS basis function. Since the weights ${w_{i}}$ are now associated with B-splines ${B_{i,p}}$ the mapping (17) employs control points ${{\varvec{c}}_{i}}$ of the model space. In general, NURBS curves degenerate to B-spline curves, if all weights are equal. Hence, they are a generalization of them. The properties of B-spline curves apply to their rational counterpart as well, if the weights are non-negative, which is usually the case.

### Spline Interpolation

In case of a spline interpolation problem, a given function *f* shall be approximated by a B-spline patch $I_{h} f := {\sum _{i=0}^{I-1}} { B_{i,p} } {c_{i}}.$ They agree at $I$ data sites ${\bar{u}}$ if and only if

19 $$\begin{aligned} f\left( {\bar{u}}_{j}\right) = {\sum _{i=0}^{I-1}} { {B_{i,p}}\left( {\bar{u}}_{j}\right) } {c_{i}}, \quad j= 0,\ldots ,I-1. \end{aligned}$$

The corresponding system of equations consists of the unknown coefficients ${c_{i}}$ and the *spline collocation matrix* ${{\mathbf {A}}_{u}}$ which is defined by

20 $$\begin{aligned} {{\mathbf {A}}_{u}} [j,\,i] = {B_{i,p}}\left( {\bar{u}}_{j}\right) , \quad i,\,j= 0,\ldots ,I-1. \end{aligned}$$

The *Schoenberg*–*Whitney theorem* [34, 83] states that the matrix ${{\mathbf {A}}_{u}}$ is invertible if and only if

21 $$\begin{aligned} {B_{i,p}}\left( {\bar{u}}_{i}\right) \ne 0, \quad i= 0,\ldots ,I-1. \end{aligned}$$

Since condition (21) guarantees that ${{\mathbf {A}}_{u}}$ does not become singular, it is expected that the corresponding condition number gets large if ${\bar{u}}$ approaches the limits of its allowed range. Non-uniformity of ${\bar{u}}$ is another reason for an increasing condition number. In fact, it gets arbitrary large if two interpolation sites approach each other, while the others are fixed. Several authors [11, 34, 184] recommend to interpolate at the *Greville abscissae* $u^{g}$ which are obtained by the following knot average

22 $$\begin{aligned} {u^{g}_{i}}&= \frac{{u_{i+1}}+{u_{i+2}} + \cdots +{u_{i+p}}}{p}. \end{aligned}$$

These abscissae are well known in CAGD and used for different purposes, e.g., to generate a linear geometrical mapping [83]. The most important feature of this approach is that it induces a stable interpolation scheme for moderate degrees $p.$

### Tensor Product Surfaces

**Fig. 7:**
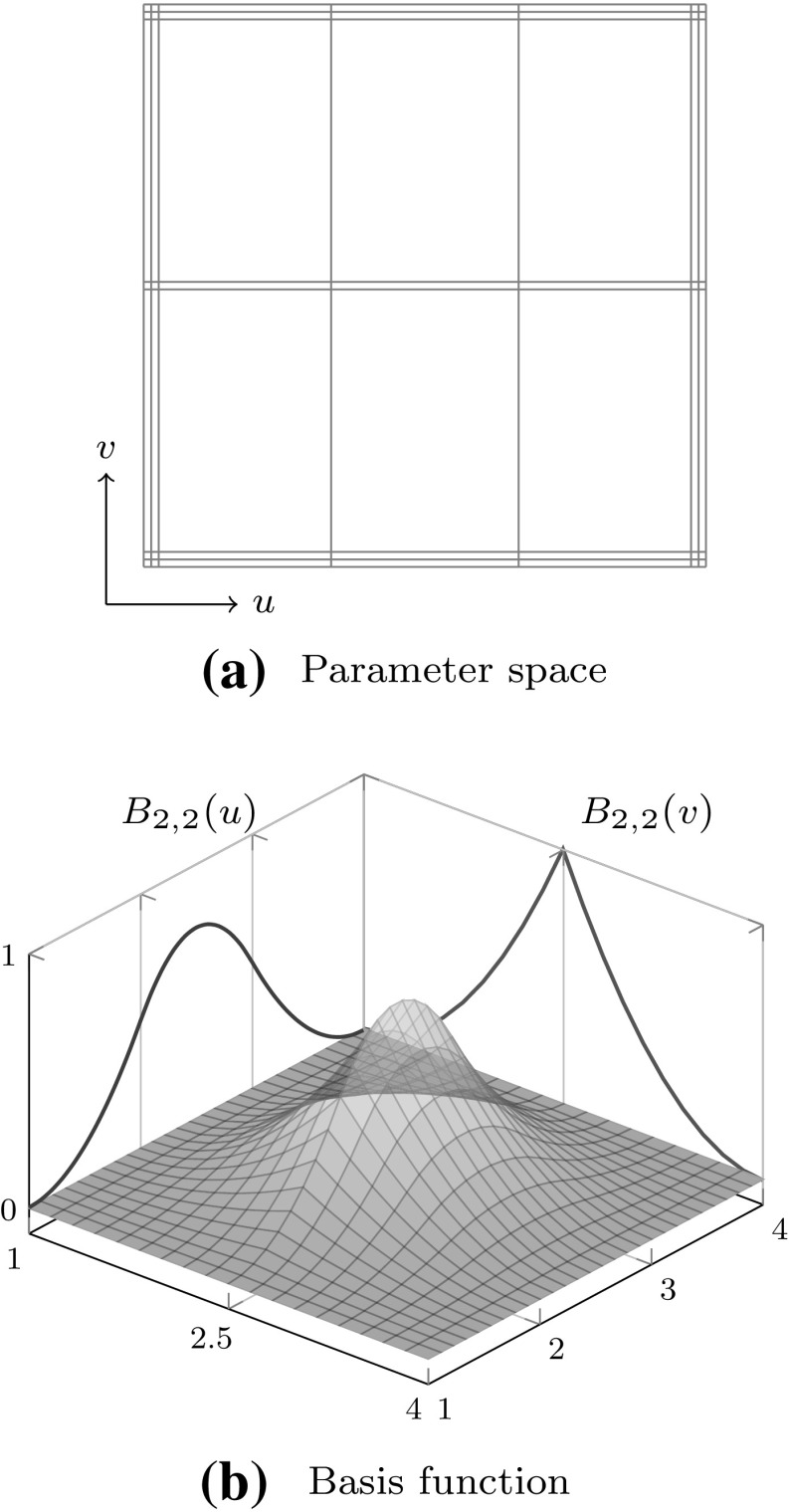
A bivariate basis determined by ${\varXi _{I}}=\{1,\,1,\,1,\,2,\,3,\,4,\,4,\,4\}$ and ${\varXi _{J}} = \{1,\,1,\,1,\,2.5,\,2.5,\,4,\,4,\,4\}$ for $u$ and $v,$ respectively: **a** shows the bivariate basis spanned by ${\varXi _{I}}$ and ${\varXi _{J}},$ whereas **b** illustrates the construction of a corresponding bivariate B-spline

Tensor product surfaces allow an extremely efficient evaluation of patches. They play an important role in CAGD. In particular, B-spline and NURBS patches are very common. Bivariate basis functions for B-spline patches are obtained by the tensor product of univariate B-splines which are defined by separate knot vectors ${\varXi _{I}}$ and ${\varXi _{J}}.$ These knot vectors determine the parameterization in the directions $u$ and $v,$ respectively. Moreover, they span the bivariate basis of a patch. Combined with a bidirectional grid of control points ${{\varvec{c}}_{i,j}}$ the geometrical mapping ${\mathcal {X}}$ is determined by

23 $$\begin{aligned} {\mathcal {X}}{(u,\,v)} := {\varvec{S}}{(u,\,v)} = {\sum _{i=0}^{I-1}} {\sum _{j=0}^{J-1}} {B_{i,p}} (u) {B_{j,q}}{(v)} {{\varvec{c}}_{i,j}}. \end{aligned}$$

The polynomial degrees are denoted by $p$ and $q,$ respectively for each parametric direction. The Jacobian of the mapping (23) is computed by substituting the occurring univariate B-splines by their first derivatives, alternately for each direction. In general, derivatives of B-spline patches are specified by

24 $$\begin{aligned} \frac{\partial ^{k+l} }{ \partial ^{k} u\partial ^{l} v} {\varvec{S}}(u,v)&= {\sum _{i=0}^{I-1}} {\sum _{j=0}^{J-1}} {B^{(k)}_{i,p}} (u) {B^{(l)}_{j,q}}(v) {{\varvec{c}}_{i,j}}. \end{aligned}$$

The efficiency of tensor product surfaces stems from the fact that their evaluation can be performed by a successive evaluation of curves [62]. Suppose the parametric value ${v^{iso}}$ is fixed, the surface equation yields

25 $$\begin{aligned} {\varvec{S}}(u,\,{v^{iso}})&= {\sum _{i=0}^{I-1}} {\sum _{j=0}^{J-1}} {B_{i,p}} (u) {B_{j,q}}({v^{iso}}) {{\varvec{c}}_{i,j}} \nonumber \\&= {\sum _{i=0}^{I-1}} {B_{i,p}}(u) \left( {\sum _{j=0}^{J-1}} {B_{j,q}}({v^{iso}}) {{\varvec{c}}_{i,j}}\right) \nonumber \\&= {\sum _{i=0}^{I-1}} {B_{i,p}}{(u)} {\tilde{{{\varvec{c}}}}_{i}} = {{\varvec{C}}^{iso}}{(u)}, \end{aligned}$$

with ${{\varvec{C}}^{iso}}{(u)}$ denoting an *isocurve* of the surface defined by new control points ${\tilde{{\varvec{c}}}_{i}}.$ Hence, a surface can be evaluated by $I+1$ or $J+1$ curve evaluations, depending which parametric direction is evaluated first.

The tensor product nature of the patches is illustrated in Fig. 7 by means of a bivariate basis. Note that the univariate knot values propagate through the whole parameter space. If both knot vectors of the resulting patch are of form (3), it is referred to as Bézier surface. NURBS surfaces are derived analogous to curves by the introduction of weights.

### Constructing Patches by Boundary Curves

The most basic surface construction scheme is to connect two curves ${{\varvec{C}}_{i}}$ with $i=1,\,2$ by a linear interpolation. The resulting surfaces are termed *ruled surfaces* and they are defined as

26 $$\begin{aligned} {{\varvec{S}}^{r}}(u,\,v)&= (1-v){{\varvec{C}}_{1}}(u) + v{{\varvec{C}}_{2}} (u) \nonumber \\&= (1-v){{\varvec{S}}^{r}}(u,\,0) + v{{\varvec{S}}^{r}}(u,\,1), \end{aligned}$$

where $u,\,v\in [0,\,1].$ If ${{\varvec{C}}_{i}}$ have the same degree and knot vector, it is straightforward to represent ${{\varvec{S}}^{r}}$ as a single tensor product surface. In this case the connection lines on ${{\varvec{S}}^{r}}$ associate points of equal parameter value. Alternatively, the rulling (26) could also be performed according to relative arc length. This yields a different geometry which cannot be converted to a NURBS patch [230].

**Fig. 8:**
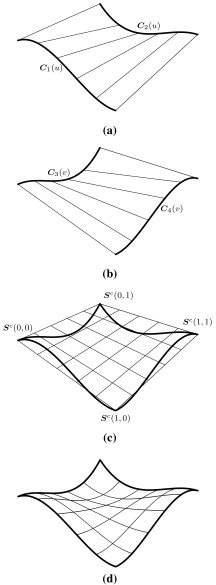
Components of a bilinear Coons patch due to the boundary curves ${{\varvec{C}}_{i}}(u)$ and ${{\varvec{C}}_{j}}(v)$ highlighted by *thick lines*

The construction of *Coons patches* is another very common procedure. Thereby, a surface ${{\varvec{S}}^{c}}$ is sought to fit four boundary curves ${{\varvec{C}}_{i}}(u)$ and ${{\varvec{C}}_{j}}(v)$ with $i=1,\,2$ and $j=3,\,4.$ The parameter range is again $u,\,v\in [0,\,1].$ The curves have to satisfy the following compatibility conditions at the corners of the surface

27 $$\begin{aligned} {{\varvec{S}}^{c}}(0,\,0)&={{\varvec{C}}_{1}}(u=0)={{\varvec{C}}_{3}}(v=0),\end{aligned}$$

28 $$\begin{aligned} {{\varvec{S}}^{c}}(1,\,0)&={{\varvec{C}}_{1}}(u=1)={{\varvec{C}}_{4}}(v=0),\end{aligned}$$

29 $$\begin{aligned} {{\varvec{S}}^{c}}(0,\,1)&={{\varvec{C}}_{2}}(u=0)={{\varvec{C}}_{3}}(v=1),\end{aligned}$$

30 $$\begin{aligned} {{\varvec{S}}^{c}}(1,\,1)&={{\varvec{C}}_{2}}(u=1)={{\varvec{C}}_{4}}(v=1). \end{aligned}$$

Using a bilinear interpolation a Coons patch is given by

31 $$\begin{aligned} {{\varvec{S}}^{c}}(u,\,v) = {{\varvec{S}}^{r}_{u}}(u,\,v) + {{\varvec{S}}^{r}_{v}}(u,\,v) - {{\varvec{S}}^{r}_{c}}(u,\,v), \end{aligned}$$

where ${{\varvec{S}}^{r}_{u}}$ and ${{\varvec{S}}^{r}_{v}}$ are ruled surfaces based on ${{\varvec{C}}_{i}}(u)$ and ${{\varvec{C}}_{j}}(v),$ respectively, and ${{\varvec{S}}^{r}_{c}}$ is the bilinear interpolant to the four corner points

32 $${\varvec{S}}_{c}^{r}(u,\;v) = {\left[ {\begin{array}{{c}} 1\\ u \end{array}} \right]^{\intercal}}\left[ {\begin{array}{{cc}} {{{\varvec{S}}^{c}}(0,\;0)}&{{{\varvec{S}}^{c}}(0,\;1)}\\ {{{\varvec{S}}^{c}}(1,\;0)}&{{{\varvec{S}}^{c}}(1,\;1)} \end{array}} \right]\left[ {\begin{array}{{c}} 1\\ v \end{array}} \right].$$

These various parts of a Coons patch are visualized in Fig. 8. Equation (31) can be generalized by using two arbitrary smooth interpolation functions ${f_{0}}(s)$ and ${f_{1}}(s)$ fulfilling

33 $$\begin{aligned} {f_{k}}(\ell )={\delta _{k\ell} }, \quad k,\,\ell =0,\,1, \end{aligned}$$

and

34 $$\begin{aligned} {f_{0}}(s)+{f_{1}}(s)=1, \quad s \in [0,\,1], \quad s=u,\,v. \end{aligned}$$

The corresponding Coons patch can be expressed in matrix form as

35 $$\begin{array}{ccccc} & {{\varvec{S}}^{c}}(u,\;v) = \\ & \quad - {\left[ {\begin{array}{{c}} { - 1}\\ {{f_{0}}(u)}\\ {{f_{1}}(u)} \end{array}} \right]^{\intercal}}\left[ {\begin{array}{{ccc}} \varvec{0}&{{{\varvec{S}}^{c}}(u,\;0)}&{{{\varvec{S}}^{c}}(u,\;1)}\\ {{{\varvec{S}}^{c}}(0,\;v)}&{{{\varvec{S}}^{c}}(0,\;0)}&{{{\varvec{S}}^{c}}(0,\;1)}\\ {{{\varvec{S}}^{c}}(1,\;v)}&{{{\varvec{S}}^{c}}(1,\;0)}&{{{\varvec{S}}^{c}}(1,\;1)} \end{array}} \right] \left[ {\begin{array}{{c}} { - 1}\\ {{f_{0}}(v)}\\ {{f_{1}}(v)} \end{array}} \right], \end{array}$$

with ${\varvec{0}}\in {{\mathbb {R}}^{d}}$ denoting the zero vector. Various functions may be used to specify ${f_{k}}$ such as Hermite polynomials or trigonometric functions. In case of Bernstein polynomials, the surfaces ${{\varvec{S}}^{r}_{u}},$ ${{\varvec{S}}^{r}_{v}},$ and ${{\varvec{S}}^{r}_{c}}$ are in Bézier or B-spline form and the resulting Coons patch can be represented as a single NURBS surface.

Finally, *Gordon surfaces* are a further generalization of Coons patches, where the surface ${{\varvec{S}}^{r}_{u}}$ and ${{\varvec{S}}^{r}_{v}}$ interpolate sets of isocurves rather than boundary curves. Gordon surfaces are also referred to as *transfinite interpolation* [103]. The term indicates that these surfaces interpolate an infinite number of points, i.e., the boundary curves and isocurves. Based on this definition, ruled surfaces, Coons patches, and Gordon surfaces may be generally referred to as transfinite interpolations.

### Representation of Triangles

**Fig. 9:**
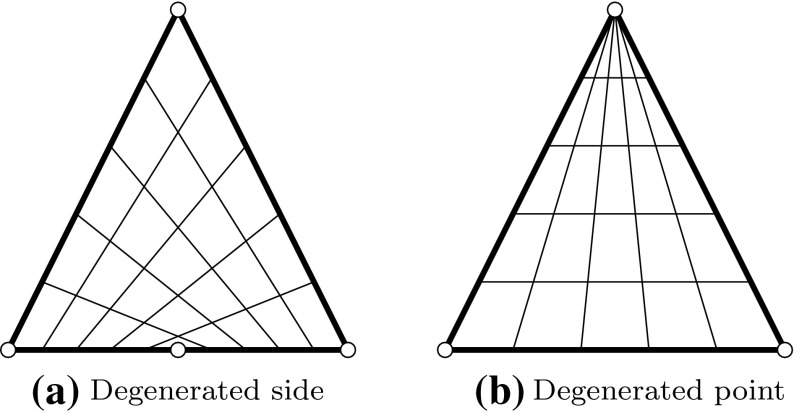
Tensor product representation of triangular patches: **a** an angle between adjacent edges has 180$^{\circ }$ and **b** a side shrinks to a point. *Circles* mark the corner points of the resulting patch

**Fig. 10:**
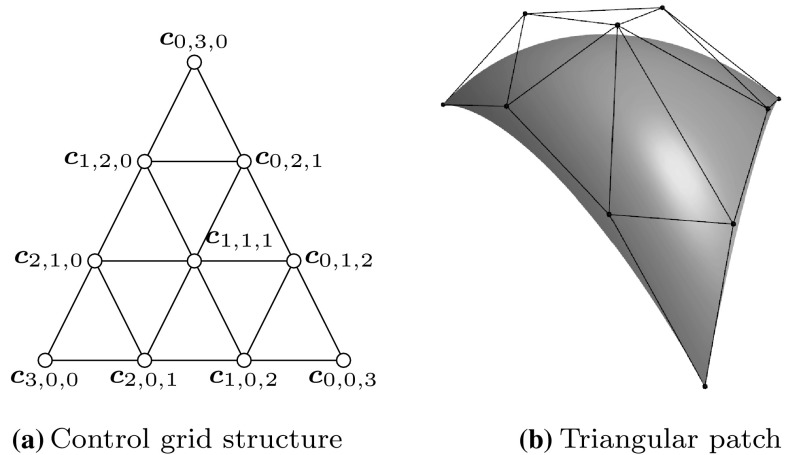
Triangular Bézier patch of degree $p=3{\text {:}}$ **a** the general structure of the control grid and **b** a corresponding surface

Triangular patches may be represented by tensor product surfaces despite their four-sided nature. Therefore, either a side or a point is degenerated as shown in Fig. 9. Such degenerated patches are often used since it is convenient to use only one surface representation. However, it is apparent that this can lead to a distorted parameterization. In addition, the enforcement of continuity between adjacent surfaces is difficult in this case.

An alternative is to use *triangular patches*. A point on such surfaces is defined by barycentric coordinates, i.e., $(r,\,s,\,t)$ with $r+s+t=1.$ We will focus on triangular Bézier patches ${{\varvec{S}}^{\triangle }}$ which are specified as

36 $$\begin{aligned}{ {\varvec{S}}^{\triangle} }(r,\,s,\,t) = \sum _{\begin{array}{c} i+j+k=p\\ i,\,j,\,k\geqslant 0 \end{array}} {B_{i,j,k,p}}(r,\,s,\,t) {{\varvec{c}}_{i,j,k}}, \end{aligned}$$

with

37 $$\begin{aligned} {B_{i,j,k,p}}(r,\,s,\,t) = \frac{p!}{i!j!k!} {r^{i}} {s^{j}} {t^{k}}, \end{aligned}$$

representing linearly independent bivariate Bernstein polynomials of degree $p.$ The related control points ${{\varvec{c}}_{i,j,k}}$ form a triangular array as shown in Fig. 10 for the cubic case. The resulting patch fulfills the convex hull property and its boundaries are Bézier curves. Rational triangular Bézier patches may be defined again by the introduction of weights. Despite the potential of triangular patches, there are currently no commercial CAD applications that admit the use of splines on triangulations.

### Trimmed Surfaces

**Fig. 11:**
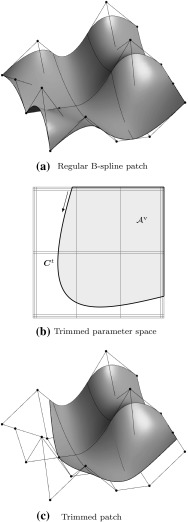
Trimmed tensor product surface: **a** regular surface defined by a tensor product basis, **b** trimmed parameter space where a loop of trimming curves (*thick line*) specifies the visible part ${{\mathcal{A}}^{\text{v}}}$ of **c** the resulting trimmed surface as displayed by a CAD system’s graphics display. The *arrow* in **b** denotes the direction of the trimming curves

In order to represent arbitrary surface boundaries when using tensor product surfaces, patches can be modified by trimming procedures. For this purpose, curves are defined within the parameter space of a surface ${\varvec{S}}(u,\,v).$ These *trimming curves* ${{\varvec{C}}^{t}}({\tilde{u}})$ are usually B-spline or NURBS curves. They are given by

38 $$\begin{aligned} {{\varvec{C}}^{t}}({\tilde{u}}) = \left[ \begin{array}{c} u({\tilde{u}}) \\ v({\tilde{u}}) \end{array}\right] = {\sum _{i=0}^{I-1}} {R_{i,p}}({\tilde{u}}) {{\varvec{c}}^{t}_{i}}, \end{aligned}$$

where ${{\varvec{c}}^{t}_{i}} \in {{\mathbb {R}}^{2}}$ are the control points of the trimming curve given in the parameter space of the trimmed surface. Connected trimming curves are ordered such that they form a closed directed *loop*. Loops also include the boundary of the original patch if it is intersected by trimming curves. These loops divide the resulting *trimmed patch* into distinct parts where the curves’ directions determine which parts are visible. In other words, trimming procedures are used to define visible areas ${{\mathcal {A}}^{\text{v}}}$ over surfaces independent of the underlying parameter space.

As a result, surfaces with non-rectangular topologies can be represented in a very simple way. An example of a trimmed patch is shown in Fig. 11. It is emphasized that the mathematical description, i.e., the tensor product basis and the related control grid, of the original patch does *not* change and is *never* updated to reflect the trimmed boundary represented by the independent trimming curves. Trimmed surfaces should be considered as an “engineering” extension of tensor product patches [83]. On the one hand, they permit a convenient way to define arbitrary surface topologies and provide a means for visually displaying them in graphics systems. On the other hand, they do not offer a canonical solution to related problems such as a smooth connection of two adjacent patches along a trimming curve, although the graphics system leads the user to incorrectly believe so. In fact, enormous effort has been and is still devoted to resolve the shortcomings of trimming procedures as discussed later on in Sect. 3.

### Solid Models

Most CAGD objects are geometrically represented by their boundary only. In other words, these models consist of several boundary patches $\gamma$

39 $$\begin{aligned} \Gamma = {\bigcup _{i=1}^{I}} {\gamma _{i}}, \end{aligned}$$

where $\Gamma$ denotes the entire boundary of the object. If $\Gamma$ is a curve, several patches may be needed to represent distinct sections with different polynomial degrees. This is not a critical issue since curves can be joined rather easily, even with a certain continuity. However, a problem arises as soon as surfaces are considered, because tensor product surfaces are four-sided by definition. A single regular NURBS surface may be closed equivalently to a cylinder or a torus. Spherical objects may be represented as well, if degenerated edges are introduced. Yet, more complicated objects such as a double torus require a partition into *multiple* NURBS patches. The connection of two adjacent surfaces is complicated, especially if a certain continuity is desired. In general, *non-conforming* parameterizations along surface boundaries need to be expected.

In addition to the geometric representation of the boundary patches, the *topology* of the object has to be described. It addresses the connectivity of the various components, and the corresponding entities are termed

- *vertices* relating to points,
- *edges* relating to curves,
- *faces* relating to surface.

**Fig. 12:**
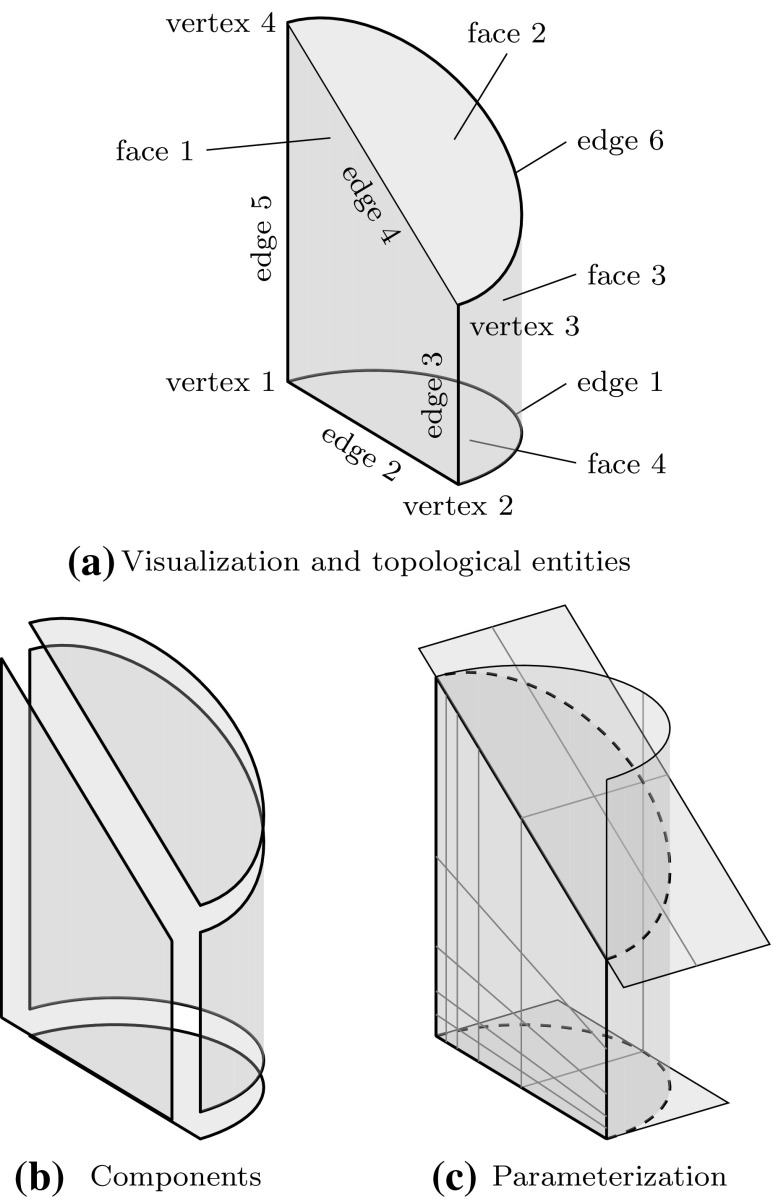
Different perspectives of a CAGD solid model: **a** visible part of the object and its topological entities (to be precise, the related geometric objects, i.e., points, curves, and surfaces, are displayed), **b** the geometric segments of the B-Rep and **c** the underlying mathematical parameterization of each surface. In **c**, *dashed lines* mark the boundary of the visible area and *gray lines* indicate the underlying tensor product basis. Note that the parameterization along common edges does not match

It should be noted that the descriptions of an object’s shape, i.e., geometry, and its structure, i.e., topology, are separated [290]. By definition, a B-Rep model always consists of a data structure of both topological and geometric objects. Regarding isogeometric analysis, the parameterization of the geometry is a further important issue that should be taken into account. Figure 12 summarizes these various perspectives of a CAGD model representing a simple solid. The corresponding object consists of three trimmed surfaces and an untrimmed, or regular one. It is apparent that even simple CAGD models rely on multiple non-conforming surfaces.

Finally, it should be mentioned that B-Reps are also used to describe dimensionally reduced objects, i.e., shell structures. In this case, the patches $\gamma$ specify the object itself rather than its boundary. It is important to note that the terms *surface* model and *solid* model do not refer to the dimension of an object. In CAGD, they rather indicate if a model contains topology information (solid model) or not (surface model). Based on the brief outline given here, the discussion on the representation of CAGD models will be continued in Sect. 3.2.

## Trimming in Computer Aided Geometric Design

There is a large body of literature on trimmed B-spline and NURBS geometries in CAGD. Trimming is addressed in the context of surface intersection, the development for appropriate data structures for solid modeling, the visualization of objects which is referred to as rendering, and remodeling approaches. The following outline of these topics is meant to be comprehensive, but it is by no means complete. Further, some auxiliary techniques are presented.

The motivation for this section is twofold: first of all, it provides an overview of the historical development of trimming approaches in the field of CAGD. Apart from being interesting in its own right, this insight exposes a number of general challenges, techniques, and ideas regarding trimmed geometries. Hence, it is hoped that the subsequent sections also give insight to further strategies dealing with trimming in the context of isogeometric analysis.

### Surface Intersection

Trimming is closely related to the problem of surface-to-surface intersection. In general, the intersection of two parametric surfaces

40 $$\begin{aligned} {{\varvec{S}}_{1}}(u,\,v)&= \left( {x_{1}}(u,\,v),\, {y_{1}}(u,\,v),\, {z_{1}}(u,\,v) \right) , \end{aligned}$$

41 $$\begin{aligned} {{\varvec{S}}_{2}}(s,\,t)&= \left( {x_{2}}(s,\,t),\, {y_{2}}(s,\,t),\, {z_{2}}(s,\,t) \right) , \end{aligned}$$

leads to a system of three nonlinear equations, i.e., the three coordinate differences of ${{\varvec{S}}_{1}}$ and ${{\varvec{S}}_{2}}$, with four unknowns $u,\,v,\,s,\,t$ [62]. If surfaces intersect, the solution usually yields curves, but also subsurfaces or points may occur. The computation of intersections is one of many “geometric interrogation” techniques, or processes, employed in all types of modeling. The development of a good surface intersection scheme is far from trivial since the method has to balance three contradictory goals: *accuracy*, *efficiency*, and *robustness*. The surveys [219, 220] and the textbooks [4, 130, 221] provide detailed information on various approaches. Surface intersection algorithms can be broadly classified into four main categories: (i) analytic methods, (ii) lattice evaluation, (iii) subdivision methods, and (iv) marching methods.

#### Analytic Methods

**Fig. 13:**
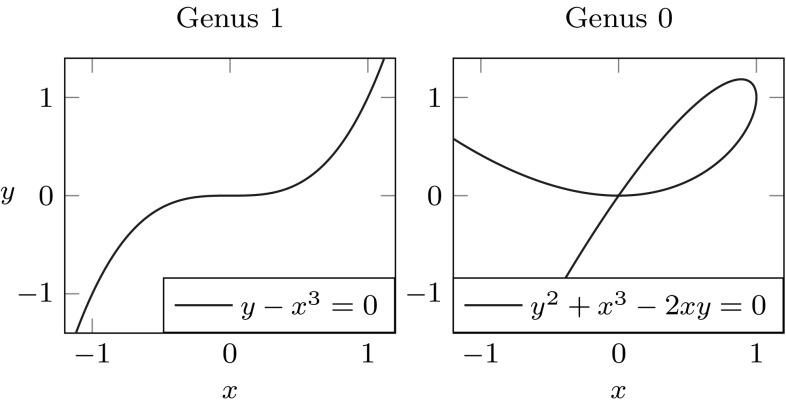
Cubic curves with different genus. Note the double point in case of the genus 0 curve

The intersection of two surfaces may be solved analytically, i.e., an explicit representation of the intersection curve is obtained. Early solid modeling systems used analytic methods to obtain exact parametric representations of the intersection of quadratic surfaces [40]. The intersection problem always has a simple solution when both surfaces are given as functions in implicit form [130]. The good news is that parametric surfaces can always be represented implicitly [265], but the main problem is that the algebraic complexity of the intersection increases rapidly with the degree of the surfaces. This is often illustrated by a popular example of the intersection of two bicubic patches which has an algebraic degree of 324 as shown by Sederberg [265, 266]. In addition, the intersection of two bicubic patches has a genus^2^ of 433 and only curves of genus 0, i.e., all degree two curves, cubic curves with one double point, quartic curves with three double points or one triple point, etc., can be expressed parametrically using rational polynomials [152]. Figure 13 illustrates two examples of implicit cubic curves with different genus. The complexity of surface intersection curves has also been discussed in the study of Farouki and Hinds [88]. It is argued that the derivation of an implicit representation is not practical and an approximation scheme may be preferred. In general, analytic methods have been restricted to low degree intersections, which yield exact results very fast.

#### Lattice Evaluation

**Fig. 14:**
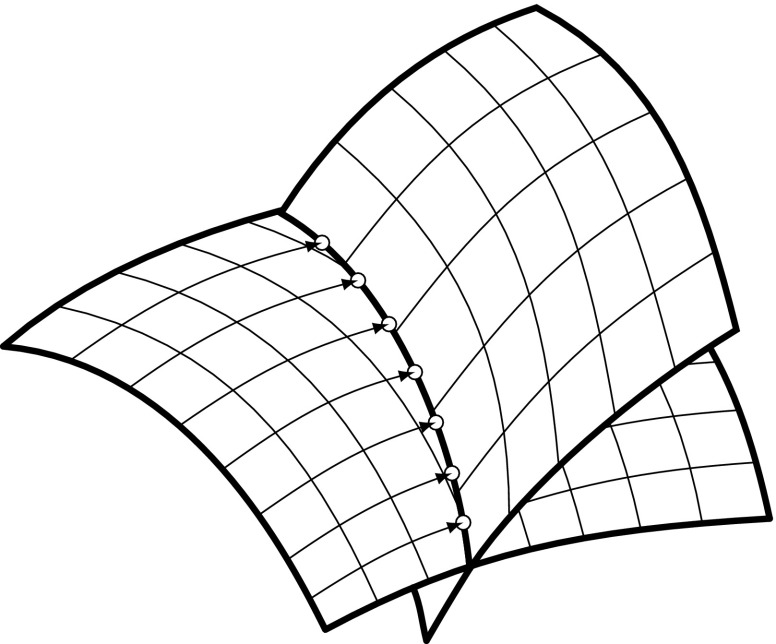
Intersection points based on line-to-surface computations

The basic idea of this technique is to reduce the dimensionality of surface intersections by computing intersections of a number of isoparametric curves [186, 250] (see Fig. 14). Once the discrete intersection points are obtained, they are sorted and connected by an interpolation scheme. In order to define an intersection curve, lattice evaluation involves an initial choice of a proper grid resolution. This is crucial for both the robustness and efficiency of the method. Unfortunately, determination of an appropriate discrete step size is not straightforward and, if too coarse, may lead to a failure in identifying critical features [170].

Curve intersection schemes are also useful in the context of ray tracing for visualization and point classification in solid modeling [220]. In these applications a patch is intersected by a straight line, as discussed later on in Sects. 3.3.2 and 3.5.2.

#### Subdivision Methods

The key idea of subdivision approaches is to compute an intersection using approximations of the patches involved, rather than the actual objects themselves. These approximations are often defined by piecewise linear elements. Consequently, the intersection problem is subdivided into many, but significantly simpler, problems. The final intersection curve is obtained by merging the individual intersection results.

**Fig. 15:**
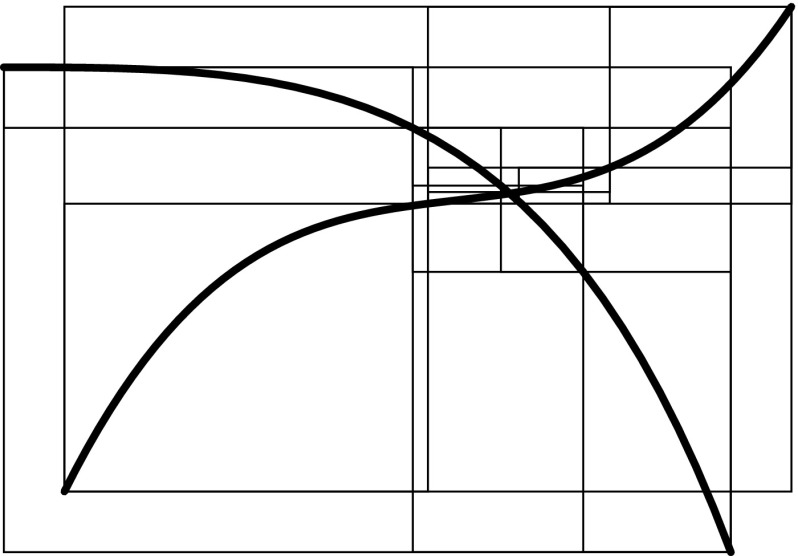
Determination of an intersection region of two curves by means of a divide-and-conquer scheme that uses axis-aligned bounding boxes

A good approximation of the original objects is of course essential for the accuracy of such techniques. However, subdivision algorithms become inefficient for high-precision evaluation, especially if the decomposition is performed uniformly. A considerable improvement can be achieved if the region of the intersection, i.e., the affected elements, is estimated in a preprocessing step. This can be carried out by *bounding boxes* that completely enclose the corresponding element. Various construction schemes of such bounding boxes are outlined in Sect. 3.5.1. Their common aim is to allow an efficient determination if two objects are clearly separated or not. In particular, elements are recursively refined if their bounding boxes overlap, which leads to a non-uniform, adaptive subdivision algorithm as shown in Fig. 15. This process of successive refinement and removing of separable boxes is referred to as a *divide-and-conquer* principle [130].

An important advantage of subdivision methods is that they do not require starting points. On the other hand, the drawbacks can be summarized according to Patrikalakis and Maekawa [221] as follows: (i) they are only able to isolate zero-dimensional solutions, (ii) there is no certainty that each root has been extracted, (iii) the number of roots in the remaining subdomains is typically not provided, and (iv) there is no explicit information about root multiplicities without additional computations. Last but not least, (v) the method is not efficient in case of high-precision or higher order evaluations [181, 219].

#### Marching Methods

Marching methods^3^ derive an intersection curve by stepping piecewise along the curve, e.g., [14, 20, 84]. Such methods usually consist of a *search*, a *marching*, and a *sorting* phase. The first phase detects an appropriate starting point on the intersecting curve. Often, this is performed by a subdivision or lattice approach. In the marching phase, a point sequence along the intersection curve is developed starting from the points determined in the previous phase. The direction and the length of the next step are defined by the local differential geometry. Finally, the individual points and segments of the intersection curve are sorted and merged to disjoint pieces and curve loops.

According to Hoschek and Lasser [130], all marching methods share some common problems: (i) determination of good starting points, (ii) detection of all branches of the intersecting curve, (iii) avoiding of multiple detections of a intersection segment, (iv) correct behavior at self-intersections and singularities, (v) proper choice of the direction and length of the subsequent step, and (vi) a robust automatic stopping criterion.

Despite all these issues, marching methods are by far the most widely used approaches due to their generality and ease of implementation [170]. In addition, accuracy improvement can be easily achieved by decreasing the step size, and they are also very efficient, especially in combination with subdivision methods.

At this point, it should be emphasized that the problems related to topology detection of the intersection curve, i.e., finding all its branches and singular points, apply to all intersection methods and several authors have addressed them, e.g., [6, 105, 168, 222, 267, 283].

#### Hybrid Methods

Every intersection method type has its benefits and drawbacks, hence a number of authors have established hybrid methods that combine features of the different categories.

One of the elementary surface intersection schemes has been proposed by Houghton et al. [133]. The algorithm combines a divide-and-conquer approach with a Newton–Raphson procedure: firstly, the surface is subdivided into flat sub-pieces. Then, each sub-piece is approximated by two triangles and the intersection of these triangles is computed. In the next step, the resulting linear segments are sorted and connected using the information provided by the subdivision tree. Finally, the intersection points are refined by the Newton-Raphson scheme. The main advantage of this method is that it is very general and can be applied to any surface representation, in contrast to earlier techniques that utilize properties of certain surface types, e.g., [46, 111, 165, 180, 224, 256].

Barnhill et al. [19] presented another general procedure to compute the intersection of two rectangular ${C^{1}}$ patches. It relies on a combination of subdivision and a marching scheme. It does not assume a special structure of the intersecting surfaces and special cases are considered, e.g., infinite plane intersections, creases, and self-intersection. The algorithm has been enhanced in [20], including the utilization of the divide-and-conquer concept presented by Houghton et al. [133].

Another combination of a divide-and-conquer subdivision with an iterative marching approach has been developed by Kriezis et al. [169]. The method enables intersecting algebraic surfaces of any degree with rational biquadratic and bicubic patches.

Krishnan and Manocha [170] developed an approach for NURBS surfaces that combines marching methods with the algebraic formulation. The starting points on the intersection curve are computed by Bézier curve–surface intersections that are obtained by eigenvalue computations. Moreover, they introduced a technique that allows detection of singularities during the tracing process.

#### Representation of the Intersection Curve

Various techniques for the computation of approximate solutions to the surface-to-surface intersection problem have been outlined so far. It remains to discuss the actual representation of the result.

In general, three *distinct* representations of an intersection are obtained. On the one hand, the intersection curve in model space is computed. This may seem to be the main objective of the whole procedure at first glance, yet it is just a part of the overall solution process. The intersection curve has to be represented in each parameter space of the trimmed patches. These curves are referred to as trimming curves in the following and are needed to determine which surface points are visible.

Intersection and trimming curves can be defined by any kind of representation, but usually low-degree B-splines are used. They are constructed based on a set of sampling points that result from the surface-to-surface intersection algorithm applied [207]. Subsequently, an interpolation scheme or another curve-fitting technique is used to generate a continuous approximation of the intersection in model space ${\hat{\varvec{C}}}.$ In general, this curve does not lie on either of the intersecting surfaces. A trimming curve ${{\varvec{C}}^{t}}$ is obtained based on the sampling points given in the corresponding parameter space [240]. The related curve ${\tilde{\varvec{C}}}^{t}$ in the model space is obtained by evaluating the equation of the surface ${\varvec{S}}$ along its ${{\varvec{C}}^{t}}.$ Alternatively, ${\tilde{\varvec{C}}}^{t}$ may be represented explicitly. DeRose et al. [71] presented an efficient and stable algorithm based on blossoming^4^ that can be use to exactly compute the control points of ${\tilde{\varvec{C}}}^{t}.$ Such an expansion of ${\varvec{S}}\circ {{\varvec{C}}^{t}}$ into an explicit representation ${\tilde{\varvec{C}}}^{t}$ may be used to join another patch to a trimmed surface, but there is no computational benefit [189]. Curves on surfaces have a degree of $p(m+n)$ with $p$ denoting the degree of the trimming curve and *m* and *n* correspond to the degrees of the trimmed surface [83]. Renner and Weiß [240] compared exact and approximate representations of ${\tilde{\varvec{C}}}^{t}$ and concluded that high degree is the main reason for preferring an approximation scheme. Furthermore, they formulated the following requirements for such a scheme: (i) low degree, (ii) fast and stable generation, (iii) full control over deviation between exact curve and approximation, and (iv) consideration of the specific surface geometry. According to them, these requirements are often not satisfied in current CAD systems. Besides the schemes presented in [240], several other approaches have been proposed to compute good approximate curves on surfaces, see e.g., [90, 119, 317].

**Fig. 16:**
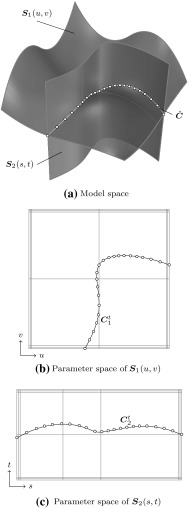
Independent curve interpolation of an ordered point set to obtain approximations of the intersection of two patches ${{\varvec{S}}_{1}}(u,\,v)$ and ${{\varvec{S}}_{2}}(s,\,t).$ The set of sampling points depends on the surface-to-surface intersection algorithm applied. The subsequent interpolation of these points is performed in **a** the model space and the parameter space of **b** ${{\varvec{S}}_{1}}(u,\,v)$ and **c** ${{\varvec{S}}_{2}}(s,\,t)$ leading to the curves ${\hat{\varvec{C}}},\,{{\varvec{C}}^{t}_{1}},$ and ${{\varvec{C}}^{t}_{2}},$ respectively. The point data is usually discarded once the curves are constructed

It is emphasized that ${\tilde{\varvec{C}}}^{t}$ does *not* coincide with the intersection curve in model space ${\hat{\varvec{C}}},$ regardless of its representation. In addition, all procedures related to trimming curves are performed for each patch separately. Hence, the images of these curves ${\tilde{\varvec{C}}}^{t}_{i}$ do not coincide, neither with each other, nor with ${\hat{\varvec{C}}}.$ As a consequence, gaps and overlaps may occur between intersecting patches. There is *no connection* between these three representations of the intersection; although the sample points provide some information during the construction, this data is only stored temporarily during the approximation procedure and never retained in memory for further use. These various approximations of a surface-to-surface intersection are summarized in Fig. 16.

Currently, the most common geometric modeling kernels are ACIS, C3D, and Parasolid. They provide software components for the representation and manipulation of objects, and form the geometric core of many CAD applications. All of them use splines for the description of trimming curves [43, 65, 282]. Yet, the representation of the intersection curve in model space varies: ACIS defines it by a three-dimensional B-spline curve, Parasolid uses a set of sorted intersection points that can be interpreted as a linear approximation, and in C3D the intersection curve is not stored at all. In C3D, trimming curves are computed such that they have the same radius and derivatives at the same parametric values. However, this is only satisfied at the intersection point used for the construction, for more details see [102].

### Solid Modeling

**Fig. 17:**
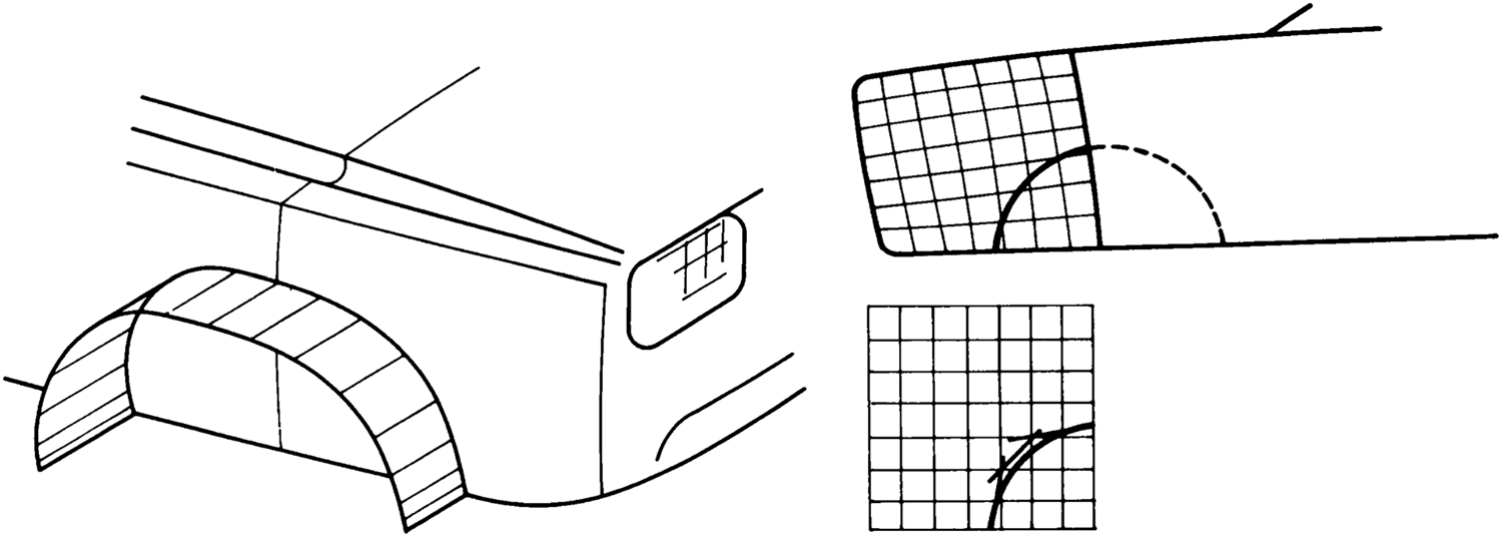
An early sketch of a trimmed surface (reprinted from [29], with permission from Elsevier)

Solid modeling is concerned with the use of unambiguous representations of three-dimensional objects. It is based on a consistent set of principles for mathematical and computer modeling, and focuses on informational completeness, physical fidelity, and universality [276]. An essential aspect is the *topology* of complex models. The consideration of topology is indeed the fundamental difference between solid models and surface models, because the latter describes only the geometry of an object [5]. Topological properties are not metrical, but address connectivity and dimensional continuity of a model [207]. There are several textbooks on solid modeling [120, 194, 207, 290] and for an elaboration of the historical development of this field of research the interested reader is referred to the landmark paper of Requicha [242] and the subsequent surveys by him and his co-authors [241, 243, 244, 251]. In the following, the progress towards a trimmed solid model as well as the related challenges are outlined.

#### Formulation of Trimmed Solid Models

Pierre Bézier outlined the idea of trimming already in the 1970s. In his paper [29], it is proposed to perform the segmenting of a model by curves defined on a square patch as illustrated in Fig. 17. These curves are termed “transposant”, the present participle of the French word for transpose. The concept reduces the amount of data and enables an easier blend with other patches. However, this idea was presented with little theoretical support and solid modeling requires an adequate mathematical theory as emphasized in a survey by Requicha and Voelcker [243].

**Fig. 18:**
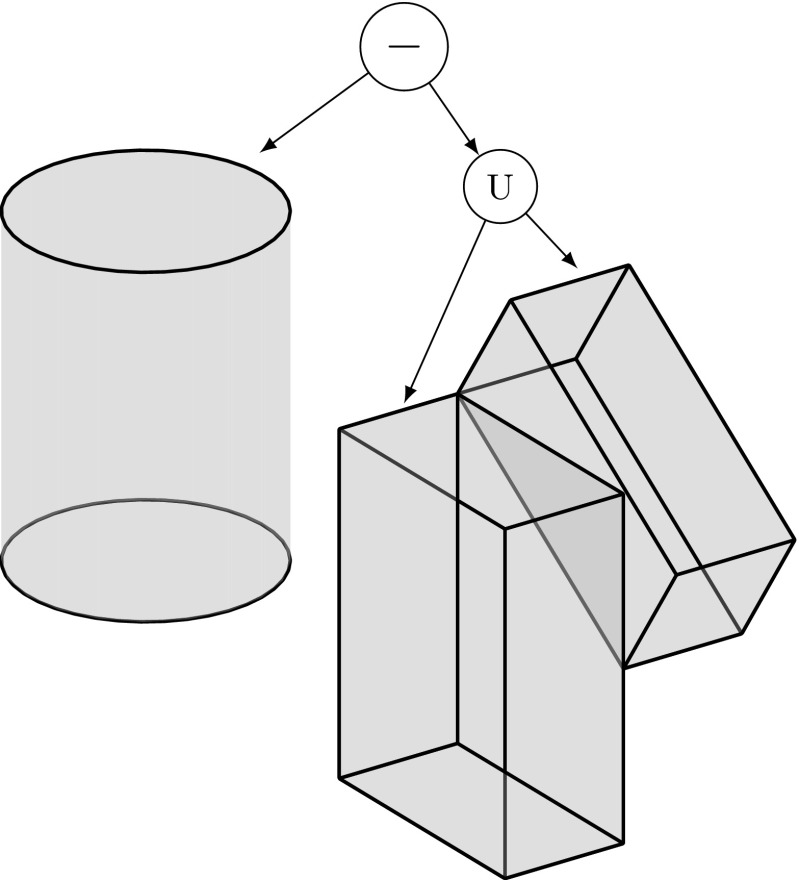
Representation of the object shown in Fig. 12a by means of a CSG tree: the object is specified by a composition of simple solids using Boolean operations, i.e., union (U) and difference (−)

It took some time to develop a rigorous way to represent trimmed free-form models. There are three broad categories for representing geometric objects: (i) decomposition, (ii) boundary, and (iii) constructive representations. Popular examples of decomposition representations are voxel models where a solid is approximated by identical cubic cells. Advantages and limitations of this approach are discussed in [153]. A B-Rep^5^ (B-Rep) defines an object by its bounded geometry, along with an associated topological structure of corresponding entities, such as faces, edges, and vertices. The benefits of storing an object’s shape by means of its boundary were already elaborated in the seminal work of Braid [36]. Most B-Reps consist of several surface patches and additional information is stored to efficiently identify the various components and their relation to each other [241]. Various data structures for B-Reps have been used, e.g., [21, 73, 106], to find a compromise between storage requirements and response to topological questions. The best known constructive representation is so-called constructive solid geometry (CSG) [241]. Simple primitives are combined by means of rigid motions and regularized Boolean operations—union, intersection, and difference. The resulting object is represented by a binary tree where the internal nodes correspond to the Boolean operations and the primitive solids (or half spaces) are given in the leaves. An example of such a tree is given in Fig. 18.

##### *Remark 2*

The developments of isogeometric analysis and additive manufacturing using heterogeneous materials yield to a growing interest in another representation of three-dimensional geometric models, namely *volumetric representations* (V-Reps). As a matter of fact, several researchers in the CAGD community have started addressing this issue, see e.g., [31, 197, 321], including the definition of trimmed V-Reps [203].

Trimmed solid models combine concepts of B-Rep and constructive representation, i.e., they consist of free-form B-Reps merged by Boolean operations [245]. A unification of CSG and free-form surfaces was presented in the early 1980s [56], perhaps for the first time. It was proposed to use models with straight edges but free-form surface interpolation in between. In the context of Boolean operations, however, the merging of B-Reps and CSG is more involved. Gossard et al. [104] developed a polyhedral modeler which combines the two representations by means of a graph structure. In particular, two relative position operators have been implemented. For manifold polyhedral objects, the implementation of Boolean operations is well understood [194]. However, the definition of a convenient representation for trimmed NURBS is a major challenge since the topology of patches becomes quite complicated if Boolean operations are performed [120]. B-Rep solid modeling utilizes surface-to-surface intersection schemes to create arbitrarily defined free-form geometric entities, but the corresponding algorithms require more than just computing the intersection curve. Weiler’s thesis [310] provides a study on topological data structures. He summarized the essential attributes of geometric modeling operators as follows:

(i) Determination of the topological descriptions,
(ii) Determination of the geometric surface descriptions,
(iii) Guarantee that the geometry corresponds unambiguously to the topology.

Setting up a topology requires the classification of the neighborhood of various entities (faces, edges, and vertices) involved in the intersections [120]. The correlation of topology and geometry becomes particularly complicated if intersection curves have singularities or self-intersections. In addition, various forms of set membership classification, i.e., the determination if parts are inside, outside, or on the boundary of a domain, are used to compute B-Reps through Boolean operations. In order to determine if a surface point is inside or outside of the surface, the trimming curve must be defined in the parameter space as noted before. If the trimming curve would only be defined in the model space, the problem would be in fact ill-defined [205].

The first formulation of a trimmed patch representation that supports Boolean operations and free-form geometry was presented in the late 1980s by Casale and Bobrow [47, 49]. The domain of trimmed patches is specified by the two-dimensional equivalent of a CSG tree. Hence, a B-Rep is obtained that contains topology information of its trimmed components. Patches are intersected similar to the divide-and-conquer procedure of [133], but the trimming curve is then also transformed into the parameter space in order to perform Boolean operations and set membership classifications. At the same time, a rigorous trimmed surface definition has been formulated by Farouki [85]. The formulation is based on Boolean operation definitions. In particular, a trimmed patch is given by its parametric and implicit surface equations together with a *trimming boundary* that is defined as a tree structure of non-intersecting and nested piecewise-algebraic loops. These loops consist of monotonic branches. The integral over the trimmed surface is determined by a proper tessellation of the patch. It should, however, be pointed out that all these approaches fail to guaranty exact topological consistency since the images of the trimming curves do not match in general, as noted by Farouki et al. [87]. This leads to gaps and overlaps of the solid model, which can introduce failure of downstream applications such as numerical simulations.

#### Robustness Issues

Several robustness issues arise in case of imprecise geometric operations. As a matter of fact, the numerical output from simple geometric operations can already be quite inaccurate. Complications may occur even for linear elements as discussed by Hoffmann [120–123] or the computation of the convex hull of a set of points [16], for instance. These problems are induced by propagation of numerical conversion, roundoff, and digit-cancellation errors of floating point representation. The issue of rounding errors of numerical computations is known for a long time, at least since an early study by Forsythe [92]. Investigating the effects of floating point arithmetic on intersection algorithms is an important area of research [220]. Since intersection problems can be expressed as a nonlinear polynomial system of equations, the robustness issue maybe addressed from a computational point of view. Troubles arise if the problem is ill-conditioned which is for example the case for tangential intersections and surface overlaps [135, 195].

**Fig. 19:**
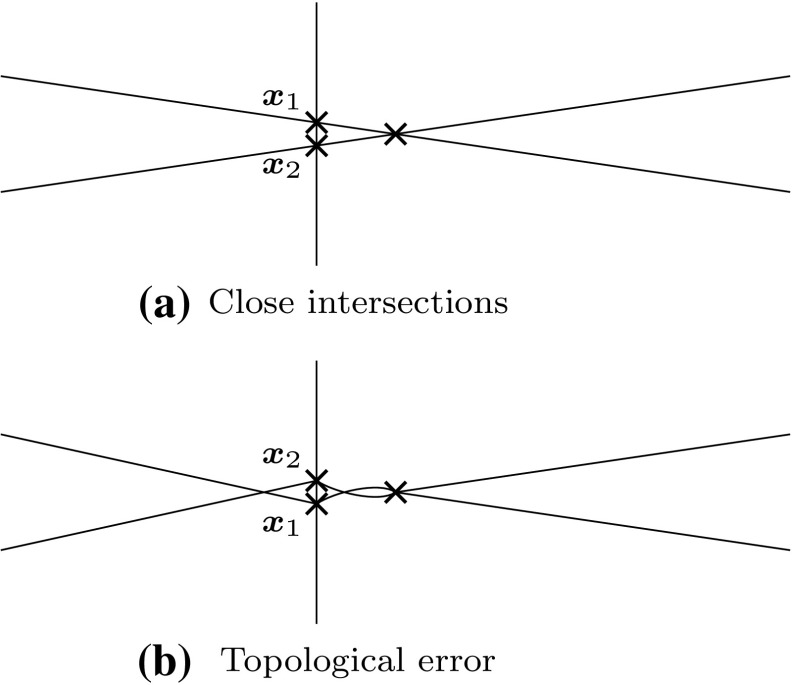
Example of an incorrect topology: **a** two intersection points ${\varvec{x}}_{i}$ are close together which may lead to **b** an incorrect topological placement along the *vertical line* due to numerical approximation errors (re-execution of the original example [204])

The key issue is that numerical errors may cause misjudgment as pointed out in [291]. Since the geometrical decisions are based on *approximate* data and arithmetic operations of *limited* precision, there is an interval of uncertainty in which the numerical data cannot yield further information [122]. Of course, the situation gets even more delicate if trimmed free-form surfaces are involved where approximation errors are quite apparent due to the gaps and overlaps between intersecting patches. In case of topological decisions, the accumulation of approximation errors is especially crucial since inaccuracy leads to inconsistency of the output as indicated in Fig. 19.

There is a large amount of research that addresses the issue of accurate and robust solid modeling. The various concepts are outlined in the following subsections. The approaches are based on tolerances, interval arithmetic, and exact arithmetic.

##### Tolerances

Often, tolerances are used to assess the quality of operations like the computation of an intersection [19, 130]. Several authors have suggested to use adaptive tolerances where each element of the model is associated with its own tolerance, e.g., [143, 271]. In addition, tolerances may be dynamically updated [82]. Robustness of topology decisions may be improved by choosing the related precision higher than the one for the input data [291]. Another strategy is to adjust the data in order to obtain topologically consistent functions [204]. There are various other approaches that improve the application of tolerance and the interested reader is referred to the review of Hong and Chang [128] for a comprehensive discussion. In fact, all common CAD software tools are based on a user-defined tolerance that determines the accuracy of the geometrical operations performed. For example, the default tolerance values of ACIS are ${10^{-6}}$ for the comparison of points and ${10^{-3}}$ for the difference of an approximate curve or surface to its exact counterpart [65]. Unfortunately, tolerances cannot guarantee robust algorithms since they do not deal with the inherent problem of limited-precision arithmetic.

##### Interval Arithmetic

In case of interval arithmetic, e.g., [76, 208], numerical errors are taken into account by associating an interval of possible values to a variable. This approach is correct in the sense that result intervals are guaranteed to contain the real number that is the value of the expression. The interval size indicates the reliability of floating point computations. In particular, a narrow interval is obtained in case of a successful operation whereas a wide interval reveals a risk [117]. This concept may be modified to rounded intervals to assure that the computed endpoints always contain the exact interval, see e.g., [3, 57, 193]. Interval arithmetic may also be combined with backward error analysis [255].

**Fig. 20:**
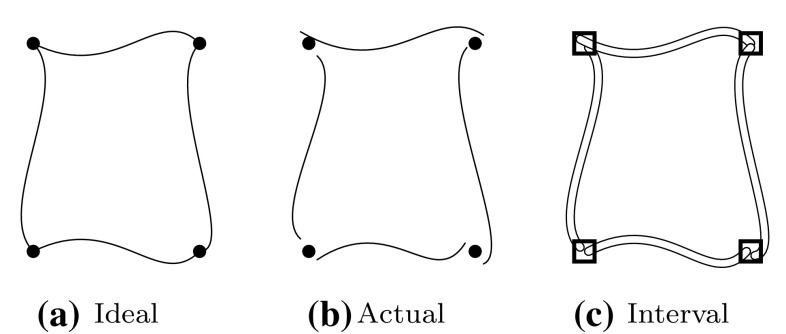
Four splines representing a quadrilateral: **a** ideal mathematical object, **b** floating point model with approximation errors, and **c** interval arithmetic based representation

In the context of solid modeling, Hu et al. [134–136] suggested to use *interval NURBS*, i.e., NURBS patches with interval arithmetic. The control points are described by interval numbers rather than real numbers. Consequently, they are replaced by control boxes and thus, curves and surfaces are represented by slender tubes and thin shells, respectively. A conceptional sketch is illustrated in Fig. 20. The object is defined by a graph with nodes representing the topological entities. Each node has two lists: one for higher dimensional nodes and another for lower dimensional nodes that are arranged in counterclockwise order. This data structure has been applied to Boolean operations [136] and various intersection problems including ill-conditioned cases [134]. Gaps between actual intersecting objects are avoided and no intersection point is missed. However, objects that do not intersect each other originally, may do after several geometric processing steps using rounded interval arithmetic. Furthermore, interval arithmetic approaches cannot achieve very high precision in reasonable computation time [220].

##### Exact Arithmetic

In order to achieve robustness, algorithms have been developed that are based on exact arithmetic, which is the standard in symbolic computation [318]. Most of these approaches focus on polyhedral objects, see e.g., [26, 93, 291]. For linear geometries like planes and their intersections, exact rational arithmetic is enough to handle all necessary numbers. However, more involved objects rely on real algebraic numbers and therefore, they require more complicated data structures and algorithms. Keyser et al. [157–159] presented such a scheme for curve-to-curve intersection in a plane and the theoretical framework for exact computation based on algebraic numbers has been discussed by Yap [318]. A combination of exact approach and floating point calculation may also be used as suggested by Hoffmann et al. [123]. In their paper, symbolic reasoning is used when floating point calculation yields ambiguous results. Krishnan et al. [171] demonstrated that exact arithmetic can be applied to large industrial models. They presented a B-Rep modeling system dealing with models using over 50,000 trimmed Bézier patches.

Still, the main drawback of such approaches is their efficiency. Exact computation can be several orders of magnitude slower than a corresponding floating point implementation [156]. According to Patrikalakis and Maekawa [220], much research remains to be done in bringing such methods to practice. In particular, more efficient algorithms should be explored that are generally applicable in low and high degree problems.

##### Concluding Remarks

Overall, the formulation of *robust* solid models with trimmed patches is still an open issue. Tolerance based approaches are usually preferred since they are faster than the more precise ones. Hence, there is again a tradeoff between efficiency, accuracy, and robustness as discussed in the context of intersection schemes. Of course, the importance of these properties to the object representation strategy depends on the application context [45].

In fact, the problem of topologically correct merging of trimmed surfaces is such a challenge that more recent research in CAGD tries to circumvent this issue by employing other surface descriptions like T-splines and subdivision surfaces. These representations inherently possess a consistent topology and corresponding models are *watertight*, i.e., they do not have unwanted gaps or holes. However, they also have some drawbacks and the transformation of the original object usually leads to approximations, at least in the vicinity of the intersection curve as discussed later on in Sect. 3.4.

### Rendering

In computer graphics rendering refers to the process of generating images of a CAGD model. There are two different ways to approach this goal. On the one hand, indirect schemes first tessellate the surfaces of the object and the actual visualization is based on this render-mesh. On the other hand, rendering may be performed directly on free-form surfaces by ray tracing. For a general introduction to the creation of realistic images, the interested reader is referred to the textbook of Glassner [99].

#### Tessellation

All commercial rendering systems tessellate free-form surfaces before rendering, because it is more efficient to optimize the code for a single type of primitive [27].

Early on, trimmed surfaces had been rendered using the de Boor [33], Oslo [60], or Boehm’s knot insertion [32] algorithm. In addition to the subdivision, the regions must be sorted to find out which ones are hidden and have to be removed for rendering, see e.g., [52, 179, 292]. In general, subdivision approaches are expensive if they are performed to pixel level.

The rendering of trimmed NURBS surfaces can also be carried out using a combination of subdivision and adaptive forward differencing [185, 275]. This method allows fast sampling of a large number of points, but suffers from error propagation. The main drawback in rendering transparent objects is the redundant pixel painting in adaptive forward differencing. Furthermore, the overall performance of the algorithm obtained is rather slow [191].

Rockwood et al. [249] presented a scheme enabling rendering of trimmed surfaces in real-time. Firstly, the surface is tessellated, i.e., approximated by linear triangles or other polygons. Therefore, all surfaces are subdivided into individual Bézier patches. A trimmed Bézier patch may be subdivided further to obtain monotone regions that have convex boundaries in the parameter space [165]. Each patch is tessellated into a grid of rectangles which are connected to the region boundaries by triangles. The actual rendering is performed on the approximate mesh. This idea has been adapted and enhanced by several other authors, e.g., [2, 176, 191].

The triangulation of trimmed surfaces by a restricted Delaunay triangulation has been proposed by Sheng and Hirsch [279]. The basic idea of this technique is to compute the approximation mesh in the parameter space. Although it has been developed for stereolithography^6^ applications, the suitability for rendering is emphasized. Stereolithography was also the motivation in [74] where trimmed surfaces are triangulated by an adaptive subdivision scheme. In contrast to the approach by Rockwood et al. [249], both algorithms contain strategies to avoid cracks between patches. A general discussion on how to avoid edge gaps in case of an adaptive subdivision is given by Dehaemer and Zyda [69].

According to Vigo and Brunet [300], the main drawback of the approaches previously mentioned [249, 279] is that the resulting elements may be odd-shaped, especially near the boundary. They suggested to overcome this issues by a piecewise linear approximation of trimmed surfaces using a triangular mesh that is based on a max-min angle criterion. The algorithm is designed so that the resulting mesh can be used for stereolithography, FEA, and rendering. The mesh obtained consists of shape-regular elements and has no cracks between patches.

The determination of a proper step size of a tessellation is of course an important issue. The elements should not be too small in order to avoid oversampling of the surface, nor too big, since this would decrease the quality of the rendering [1]. Lane and Carpenter [178] presented a formula for calculating the upper bound of the distance between a right triangle interpolating a surface. Later, the bound was improved by Filip et al. [89]. Based on this work, Sheng and Hirsch [279] derived the following formula for arbitrary triangles: the approximation error can be estimated by the difference of a parametric surface ${\varvec{S}}(u,\,v)$ to a linear triangle ${\varvec{T}}(u,\,v)$

42 $$\begin{aligned}&\underset{(u,\,v) \in T}{{{\mathrm{sup}}}} \Vert \varvec{S}(u,\,v) - \varvec{T}(u,\,v) \Vert \le \frac{2}{9} \lambda ^{2} \left( M_1+2M_{2}+M_{3}\right) , \end{aligned}$$

where *T* is the correspond region in the parameter space, $\lambda$ denotes the maximal edge length of ${\varvec{T}}(u,\,v),$ and ${M_{i}}$ are specified by

43 $$\begin{aligned} {M_{1}}&= \underset{(u,\,v) \in T}{{{\text{sup}}}} \left\| \frac{\partial ^{2} {\varvec{S}}(u,\,v)}{\partial ^{2}u} \right\| , \end{aligned}$$

44 $$\begin{aligned} {M_{2}}&= \underset{(u,\,v) \in T}{{{\text{sup}}}} \left\| \frac{\partial ^{2} {\varvec{S}}(u,\,v)}{\partial u\partial v} \right\| , \end{aligned}$$

45 $$\begin{aligned} {M_{3}}&= \underset{(u,\,v) \in T}{{{\text{sup}}}} \left\| \frac{\partial ^{2} {\varvec{S}}(u,\,v)}{\partial ^{2}v} \right\| . \end{aligned}$$

Hence, the upper bounds of second derivatives of the surface are required. Once these bounds are determined, $\lambda$ can be computed for a given tolerance $\varepsilon$ by

46 $$\begin{aligned} \lambda = 3 \left( \frac{\varepsilon }{2({M_{1}}+2{M_{2}}+{M_{3}})} \right) ^{1/2}. \end{aligned}$$

The bounds on the second derivatives for a B-spline surface (43)–(45) can be computed by constrained optimization [89] or conversion to a Chebyshev basis [279]. Piegl and Richard [229] use the fact that the derivative of a B-spline is again a B-spline to define the upper approximation bounds by computing the maxima of the control points of the differentiated surfaces. They address the treatment of rational surfaces by means of homogeneous coordinates and adjustment of the tolerance due to the perspective mapping (12).

A few years later, Piegl and Tiller [231] proposed a triangulation scheme which is geometry-based, i.e., the procedure is based on the geometry rather than the parameterization. The trimming curves are polygonized in the model space by cubic Bézier curves and the surface itself is subdivided by its control net. The main advantage of this approach is that the trimmed NURBS surface is not required to have more than ${C^{0}}$-continuity, in contrast to the previous methods that assumed that the surfaces are ${C^{2}}$-continuous in order to estimate a step length in the parameter space [89]. Elber [79] proposed two alternative approaches that are also independent of the parameterization: one based on an intermediate linear surface fit and another based on global normal curvature. In general, tessellations do not require an element connectivity or shape regular elements. However, several authors have presented the construction of conforming meshes for trimmed patches that yield triangles with good aspect ratios [55, 57, 58].

Irregular meshes are also an issue regarding hardware implementation on the graphical processing unit (GPU). Moreton [206] presented tessellation of polynomial surfaces for hardware rendering using forward differences and dividing the work of tessellation between CPU and GPU. To avoid gaps along shared boundaries of patches due to the different floating point engines, all boundary curves of the patches are calculated on the GPU. In order to enable GPU based tessellation, Guthe et al. [108] presented a *trim texture* scheme which can be parallelized. In this approach, the visible domain is specified based on a texture-map of black and white pixels, hence the trimming task is performed on pixel-level.

#### Ray Tracing

**Fig. 21:**
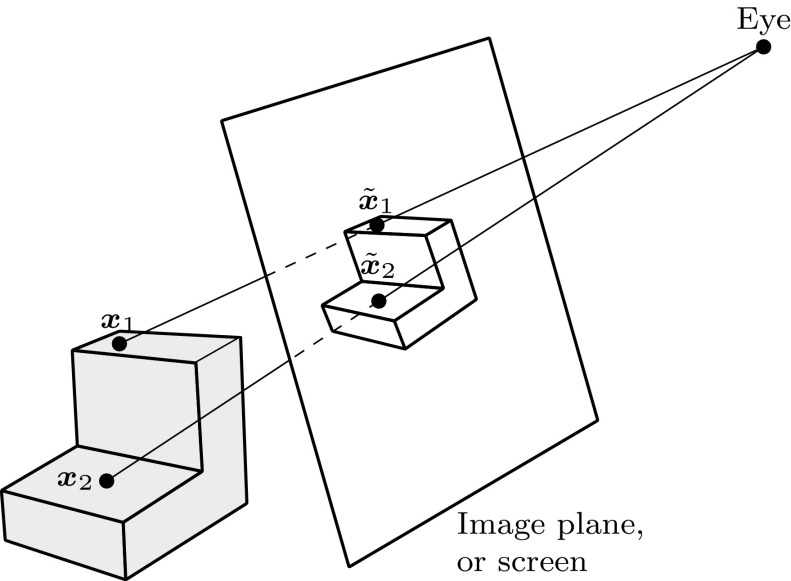
Rays spawned from an eye-point in order to get a pixel-wise image of an object

In contrast to tessellation, ray tracing tries to compute an image one pixel at a time [76]. Every object in a scene is tested if it intersects with rays spawned from an eye-point as indicated in Fig. 21. The result must return at least the closest intersection point and the corresponding normal of the surface for each ray. Hence, the heart of any ray tracing package is the set of ray intersection routines [99].

Ray tracing is a powerful, yet simple approach to image generation [144]. Already early attempts of this technique have been successfully applied for automatic shading of objects [8], modeling of global lightning effects [311], and the visualization of fuzzy reflections and blurred phenomena [64]. In the context of parametric surfaces, the pioneering works focus on different ray-surface intersection methods [144, 146, 297]. Various intersection algorithms have been developed. One of the first algorithms used a lattice approach where the problem is reduced to finding the root of univariate polynomials [146]. Several numerical methods based on Newton schemes have been employed, e.g., [198, 293, 297]. Nishita et al. [214] introduced a ray tracing technique for trimmed patches based on Bézier clipping. This concept has been adapted and enhance by a large number of researchers, e.g., [44, 78, 97, 217, 303]. Bézier clipping is discussed in more detail in Sect. 3.5.2.

Ray tracing of trimmed patches has also been addressed. Usually, the untrimmed surface is intersected first and the determination if the intersection point lies inside or outside of the trimmed domain is performed in a subsequent step. This point classification task can be employed by ray-tests, e.g., [198, 214, 261]. The regions that require trimming may be identified in a preprocessing step in order to improve the performance [97]. Section 3.5.2 provides more information on the ray-test concept. An alternative way of point classification is to generate a trim texture that returns whether a point is inside or not [108]. This approach is very efficient since it requires only a single texture look-up to classify a domain point. However, the trim texture has to be updated every time the view changes [313].

One of the greatest challenges of ray tracing is efficient execution [99]. Hence, many researchers have focused on this issue. Early attempts improved the performance by means of bounding box trees, e.g., [198, 316]. Havran [116] compared a number of such schemes and concluded that the kd-tree is the best general-purpose acceleration structures for CPU. Kd-trees define a binary space partition that always employs axis-aligned splitting planes. Pharr et al. [226] have shown that coherence can be exploited to improve ray tracing. Their rendering algorithms improve locality of data storage and data reference. Further improvement can be obtained by simplifying and streamlining the basic algorithms in order to exploit performance features of processors like single instruction, multiple data extensions [27, 97, 302]. Purcell et al. [235] demonstrated that the entire ray tracing process can be performed on the GPU. Since then, several other GPU based approaches emerged, e.g., [91, 172, 217, 261].

Despite the efficiency deficit compared to tessellation schemes, direct rendering of surfaces has several advantages. The memory requirements and preprocessing costs are reduced since fewer primitives are used and geometric precision and image quality are improved by eliminating artifacts [27].

### Remodeling of Trimmed Models

Solid models with trimmed surfaces suffer from robustness issues that may lead to inconsistencies as previously discussed in Sect. 3.2.2. In order to obtain an unambiguous and watertight description of a solid model, several authors considered replacing trimmed objects by other surface representations. In particular, it has been suggested to remodel trimmed surfaces by means of a set of regular patches, subdivision surfaces, or T-splines.

#### Regular Patches

The treatment of trimmed surfaces in the early automotive industry was discussed by Sarraga and Waters [257], in which a *repatching* method is proposed. To be precise, the intersection curves are used as edges of new regular patches approximating the original surface. As pointed out by Sarraga and Waters, repatching has several distinct disadvantages for modeling, but it is applied as a compromise between the complexity of free-form surfaces and the requirements of solid modeling. The common aim of the subsequent approaches is to improve this compromise. Besides the desire for an unambiguous and robust solid model, exchange of geometric data between dissimilar CAD software has been a motivation for this remodeling concept. Various constructions for the repatching procedure have been proposed. Hoschek and Schneider [131] convert trimmed rational Bézier patches into a set of bicubic and biquintic Bézier patches. The segmentation is based on arguments related to the curvature of the surface and conditions on the geometrical continuity. The procedure combines some of Hoschek’s previous works, i.e., [129, 132], and consists of four steps: (i) determination of new geometrically oriented boundary curves, (ii) approximation of these curves, (iii) fitting of the interior of each patch using geometric continuity conditions for the boundary and corner points, and (iv) approximation of the intersection curves of trimmed surfaces. The use of ruled surfaces [110], Coons patches [41, 301], and Clough–Tocher splines^7^ [167] have also been suggested to remodel trimmed surfaces. Another concept is based on clipping isoparametric curves of a B-spline surface [9]. Later, this approach has been adapted for the design of aircraft fuselages and wings [304, 320].

**Fig. 22:**
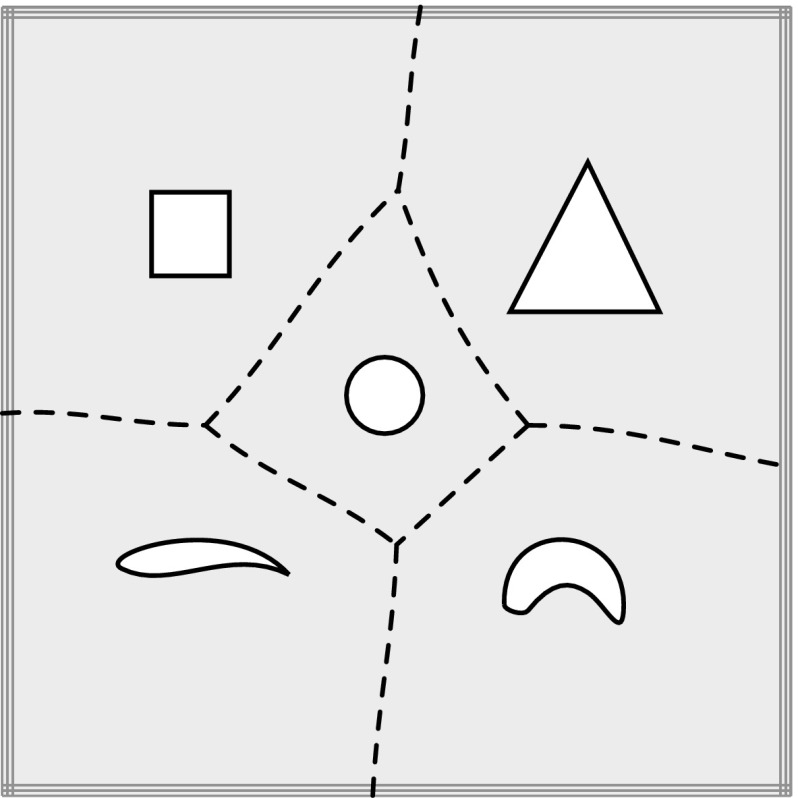
Generalized Voronoi diagram for five trimming curves (re-execution of the original figure of [110])

Generalized Voronoi diagrams may be used to obtain a proper decomposition of the trimmed domain with multiple trimming curves [110, 142]. Thereby, the parameter space is partitioned into convex polygons such that each polygon contains exactly one trimming curve as illustrated in Fig. 22. Details on Voronoi diagrams can be found in the survey of Aurenhammer [10].

Another strategy to remodel trimmed models is local *perturbation*. In contrast to repatching, the control points of the original surfaces are modified in order to obtain an unambiguous configurations along the intersection curves. Hu and Sun [137] proposed to close gaps between trimmed B-spline surface by an algorithm that moves one of the patches towards the trimming curve defined by the other one. This approach modifies the control point of the patch near the trimming curve using singular value decomposition. It can be used to improve the accuracy of *small* gaps, but yields bad-shaped surfaces if the gaps are too large. Moreover, this approach does not produce an exact topological consistency. Song et al. [285] defines the differences of corresponding trimming curves by means of a so-called error curve in model space. It is specified so that its coefficients depend linearly upon the control points of the intersecting surfaces. The perturbation is carried out by setting all coefficients of this curve to zero. This is found by solving a linear system of equations and results in an adaptation of the control points. A complement to this work was presented by Farouki et al. [87]. They propose to remodel trimmed surface by a hybrid collection of tensor product patches and triangular patches. In particular, they demonstrated the approximation of trimmed bicubic patches by quintic triangular patches such that the intersection curves are explicitly defined by one side of a triangular Bézier patch. The approach considers pairs of rectangular patches that intersect along a single diagonal arc. This can be achieved by a preprocessing step as described in the follow-up paper [115].

#### Subdivision Surfaces

**Fig. 23:**
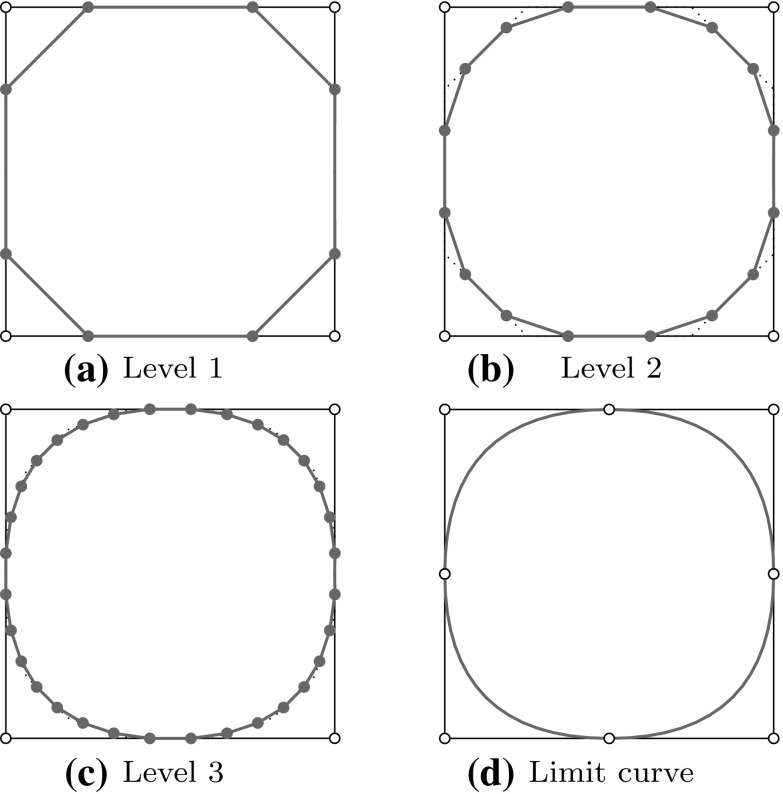
Chaikin’s corner-cutting algorithm: construction of a quadratic B-spline curve by a subdivision of the control polygon

The basic concept of subdivision approaches goes back to the 1970s. Chaikin developed an elegant algorithm to draw a curve by cutting the corners of a linear polygon [54]. The basic steps of the procedure are shown in Fig. 23. Later, it was shown that this cutting algorithm converges to a quadratic B-spline curve and the initial polygon is equivalent to its control polygon [246]. This idea of sequential subdivision of a control polygon was generalized by Doo and Sabin [75] as well as Catmull and Clark [53] to compute bi-quadratic and bi-cubic B-spline surfaces, respectively. Since then, a vast number of different subdivision schemes emerged for various surface types, such as triangular splines [190] and NURBS patches [51], for instance. The final objects of subdivision schemes are referred to as *limit* curves or surfaces. The distinguishing feature of these approaches is that they can be applied to arbitrary control polygons which are not restricted to a regular grid structure. The smoothness between the resulting surfaces is controlled by the subdivision scheme.

In 2001, the issue of Boolean operation for subdivision surfaces was addressed. Litke et al. [188] presented a trim operator, but do not address surface-to-surface intersections. An algorithm for approximate intersections was developed by Biermann et al. [30]. High accurate results can be achieved at additional computational expense. Both approaches employ subdivision based on triangular splines. Shen et al. [278] convert trimmed NURBS surfaces to untrimmed subdivision surfaces using Bézier edge conditions. The limit surface fits the original object to a specified tolerance. The resulting Catmull–Clark models are watertight and smooth along the intersection. Recently, Shen et al. [277] presented a generalization of the approach that converts B-Rep models of regular and trimmed bicubic NURBS patches to a single NURBS-compatible subdivision surface. During this process, a quadrilateral mesh topology is constructed in the parameter space of each patch and the corresponding control points are computed by solving a fitting problem. Finally, the individual parts are merged into a single subdivision mesh. In order to obtain gap-free joints, the preserved boundary curves in model space are used as target curves of the subdivision surface.

Subdivision models possess a greater flexibility due to their inherent topological consistence while conventional NURBS models have greater control of an objects shape. This attribute of subdivision attracted considerable attention, especially in the field of computer animation [70]. For a detailed discussion on subdivision schemes the interested reader is referred to the textbook [308].

#### T-splines

**Fig. 24:**
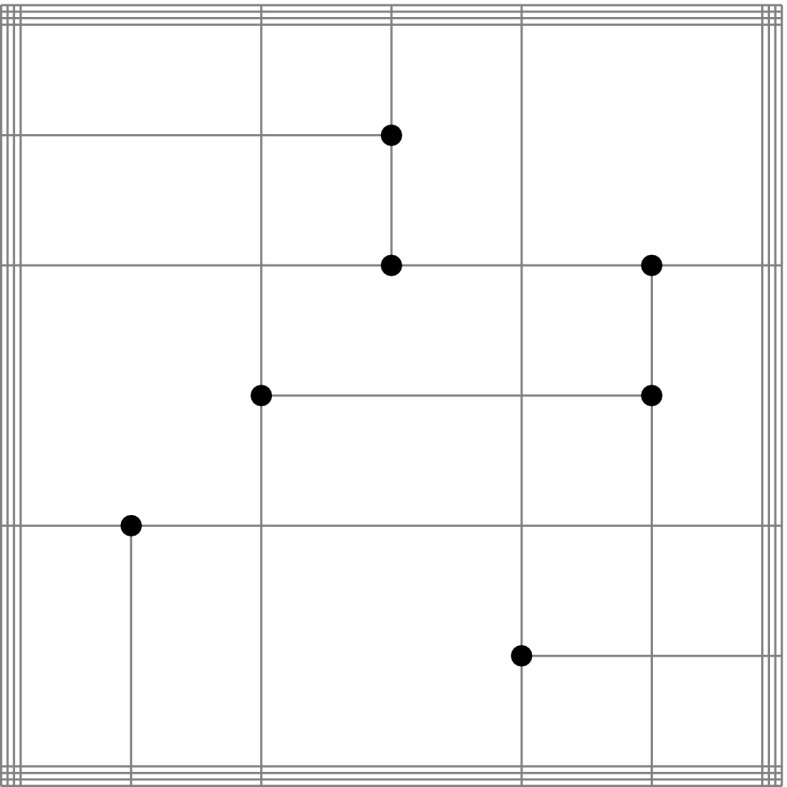
An example of a parameter space with T-junctions which are highlighted by *circles*

T-splines were introduced by Sederberg et al. [270] in 2003. They are generalizations of B-splines that allow T-junctions in the parameter space and the control net of a surface as illustrated in Fig. 24. In a subsequent paper [268], the related ability of local refinement is used to close gaps between trimmed surfaces by converting them to a single watertight T-spline model. The resulting T-spline representation can be converted to a collection of NURBS surfaces again, without introducing an approximation error. On the other hand, conversion to the T-spline representation includes some perturbation in the vicinity of the intersection. It is argued that the approximation error can be made arbitrarily small, and the perturbation can be confined to an arbitrarily narrow neighborhood of the trimming curve. The conversion is performed such that ${C^{2}}$-continuity is obtained between the intersecting surfaces. These papers are focused on cubic splines since they are the most important ones in CAGD. However, the T-spline concept is not restricted to the cubic case.

### Auxiliary Techniques

Techniques and strategies frequently used in the context of trimming are outlined in this section. They may be useful for researchers dealing with trimmed models in isogeometric analysis.

#### Bounding Boxes

Bounding boxes are often applied to significantly accelerate geometrical computations. The basic idea is to use rough approximations of objects in order to get a fast indicator if two regions are well separated or not. Hence, involved operations have to be carried out only if necessary. These approximations may be refined adaptively as in divide-and-conquer based surface intersection approaches introduced in Sect. 3.1.3.

**Fig. 25:**
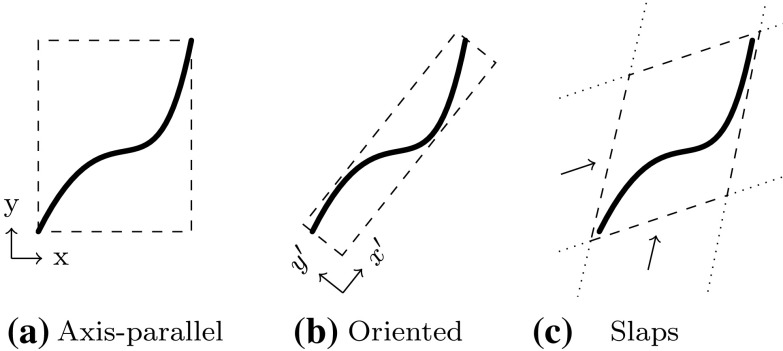
Various types of bounding boxes for the same curve. The orientation of the enclosing region is indicated by *arrows*

The simplest and perhaps most common approach is to embed objects into *min-max* boxes where the corner points of the object define an axis-parallel box. The axis aligned setting is not mandatory but allows the most efficient evaluation of the distance between two boxes [116]. Some authors suggest to use oriented bounding boxes to improve the geometry approximation, e.g., [15, 20, 133]. In this case, the bounding box is rotated such that it is aligned with the connection of the corner points of the surface it encloses. An object can also be bounded by a combination of *slaps*, also known as *fat lines*, with different orientations [154]. Slaps denote regions between two parallel planes which are specified by their normal vector. This concept includes conventional bounding boxes, simply by using two orthogonal slaps. Figure 25 summarizes these various bounding box types.

**Fig. 26:**
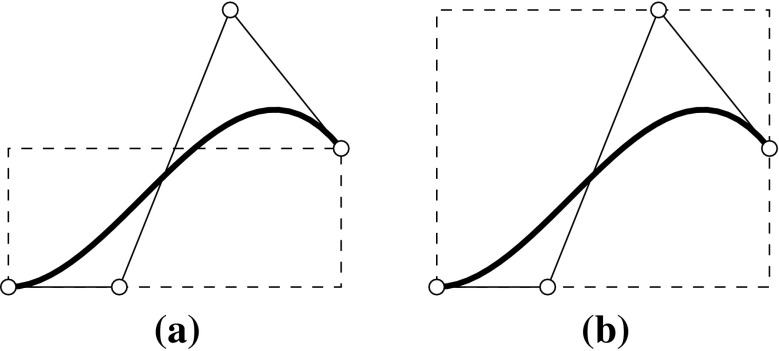
Construction of axis-parallel bounding boxes by **a** the endpoints and **b** the control points of a spline

Bounding boxes constructed by corner points do not guarantee the enclosing of the whole spline, especially if a spline is highly curved. The *convex hull* property of the control points can be used in order to get a proper approximation. Consequently, the area of the bounding box increases since it is computed based on the control polygon rather than the actual geometry, as illustrated in Fig. 26. Sederberg and Nishita [269] proposed an optimized bound for planar quadratic and cubic Bézier curves. They suggested defining the bounding region by lines parallel to the connection $\ell$ of the first and last control point. They are determined by the minimal and maximal distance ${d_{i}}$ of the other control points ${{\varvec{c}}_{i}}$ perpendicular to $\ell .$ The tighter bound is determined in the quadratic case by

47 $$\begin{aligned} {d_{min}} = \min \left\{ 0,\,\frac{{d_{1}}}{2}\right\} \quad \,\text {and}\,\quad {d_{max}} = \max \left\{ 0,\,\frac{{d_{1}}}{2}\right\} , \end{aligned}$$

while for the cubic splines it is

48 $$\begin{aligned} {d_{min}}&= \alpha \cdot \min \left\{ 0,\,{d_{1}},\,{d_{2}}\right\} ,\end{aligned}$$

49 $$\begin{aligned} {d_{max}}&= \alpha \cdot \max \left\{ 0,\,{d_{1}},\,{d_{2}}\right\} , \end{aligned}$$

with the scaling factor $\alpha$ given by

50 $$\begin{aligned} \alpha&=\left\{ \begin{array}{ll} \frac{3}{4} & { if }\,{d_{1}} {d_{2}}> 0,\\ \frac{4}{9} & { otherwise. } \end{array} \right.\end{aligned}$$

**Fig. 27:**
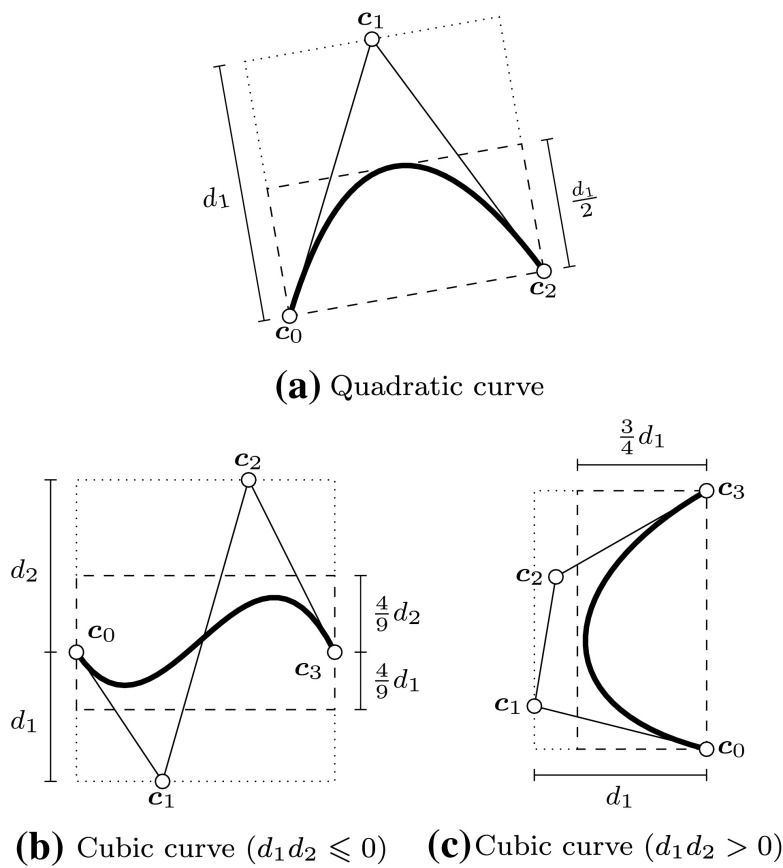
Tighter bounds for bounding boxes for quadratic and cubic B-spline curves. The original bounding boxes are shown by *dotted lines* whereas *dashed lines* mark the improved ones

Figure 27 illustrates the improvement of the bounding boxes due to these bounds. Note that the bounding boxes are oriented according to the locations of the first and last control point.

**Fig. 28:**
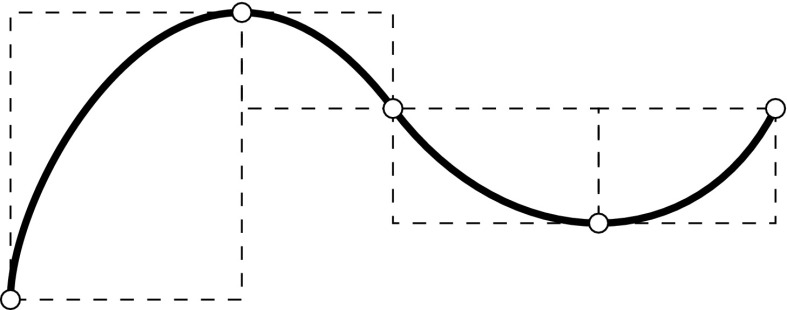
Definition of axis-parallel bounding boxes based on monotonic regions. The *white points* mark the characteristic points considered

Another way to assure that a curve lies within its bounding box is to subdivide it into *monotonic regions*. The essential idea is that if a domain of any continuously differentiable function *f* is subdivided at its characteristic values, the range of *f* on each of the subintervals can be simply found by evaluating *f* at the endpoints of that subinterval [165, 208]. The set of characteristic points may include zeros of the first or second derivatives of *f*,  start and end points of open curves, and singular points such as cusps or self-intersections. Figure 28 shows an example of a B-spline curve that has been divided into monotonic regions and the corresponding bounding boxes. In order to detect these points, a preprocessing step is required. Despite this additional effort, monotonic regions have been used in several application like intersecting planer curves [156, 157] and surfaces [84], tessellation of trimmed NURBS [249], and ray tracing [261].

#### Point Classification

**Fig. 29:**
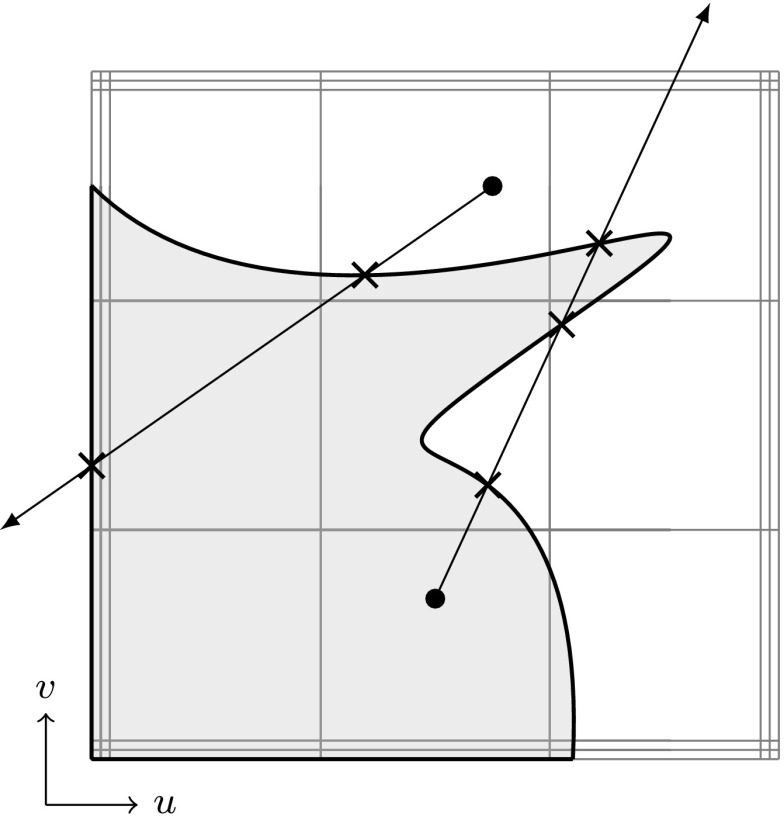
Classification of interior and exterior points by counting intersections of the trimming curve with a ray

One of the most fundamental operations in the context of trimmed surfaces is the determination if a point ${\varvec{x}}$ of a patch is inside *or* outside the visible domain. This can be done by counting the number of intersections of a ray emanating from ${\varvec{x}}$ with the trimming curves and the boundary of the patch. If the number is odd ${\varvec{x}}$ is inside and otherwise it is outside of the visible area. The direction of the ray can be chosen arbitrary. This rule is based on the Jordan curve theorem, that is, every simple *closed planar curve* separates the plane into a bounded interior and an unbounded exterior region [109]. Hence, the intersection is determined in the parameter space of the patch, in contrast to the ray tracing approach for rendering outlined in Sect. 3.3.2. Furthermore, if a trimming curve is not closed, it is associate to the visible part of the patch boundary to obtain a closed loop as illustrated in Fig. 29. Another possibility is to connect open trimming curves with the non-visible boundary of the patch and intersect only with the trimming curves. It should be noted that in the latter case, the even-odd rule turns upside down, i.e., ${\varvec{x}}$ is inside the visible domain if the number of intersections is even.

**Fig. 30:**
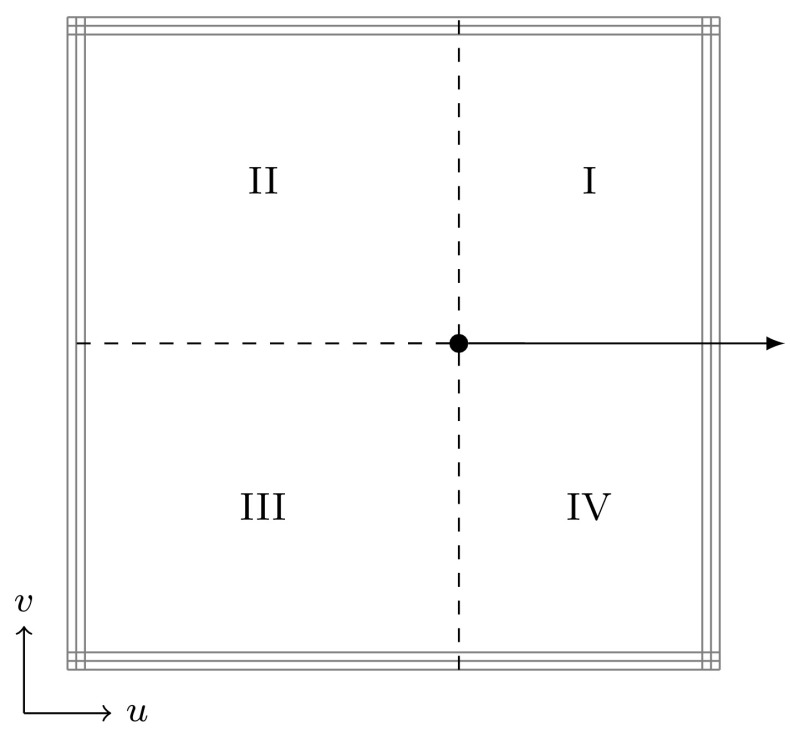
Specification of quadrants for the point classification procedure of Nishita et al. [214]

Despite its conceptional simplicity, the implementation of the corresponding algorithm is not trivial [77]. For example, ambiguous cases may occur like tangency between the ray and the curve. Nishita et al. [214] proposed the following procedure: the ray is chosen such that it intersects perpendicularly with the closest boundary of the patch. As a consequence, the parameter space is divided into four quadrants which meet at the origin of the ray as shown in Fig. 30. They are labeled counter-clockwise such that the quadrants I and IV are adjacent to the ray. If the trimming curve is specified as a set of Bézier curves the following cases may be considered:

(i) There are no intersections with the ray if *all* control points of the trimming curve are within the quadrants I and II, or II and III, or III and IV.
(ii) All control points of a trimming curve are within the quadrants I and IV. The number of intersections is even if the endpoints of curve are in the *same* quadrant; otherwise it is odd.

Tangency between the ray and the trimming curve do not pose any problem for these exclusion criteria. However, it should be pointed out that an intersection may be counted twice if the ray goes through an endpoint which is shared by two trimming curves. For the other cases where the intersection has to be computed, Nishita et al. [214] suggested to employ *Bézier clipping*. This concept has been introduced by Sederberg and Nishita [269] in the context of curve-to-curve intersection and locating points of tangency between two planar Bézier curves. The basic idea is to use the convex hull property of Bézier curves to identify regions of the curves which do not include the solution. The bounding regions are defined by fat lines parallel and perpendicular to the line through the endpoints of the Bézier curve. By iteratively clipping away such regions, the algorithm converges to the solution at a quadratic rate and with a guarantee of robustness.

In particular, the ray is defined implicitly by

51 $$\begin{aligned} a x + b y + c = 0 \quad \,\text {with}\,\quad {a^{2}}+{b^{2}}=1. \end{aligned}$$

The coordinates are denoted by *x* and *y* in order to emphasize that this approach is applicable for any plane coordinate system. The distance $d{(u)}$ of a point on the Bézier curve ${\varvec{C}}{(u)}$ to the ray $\ell$ is given by

52 $$\begin{aligned} d{(u)} = {\sum _{i=0}^{p}} {d_{i}} {B_{i,p}} \quad \,\text {with}\,\quad {d_{i}} = a {x_{i}} + b {y_{i}} + c. \end{aligned}$$

**Fig. 31:**
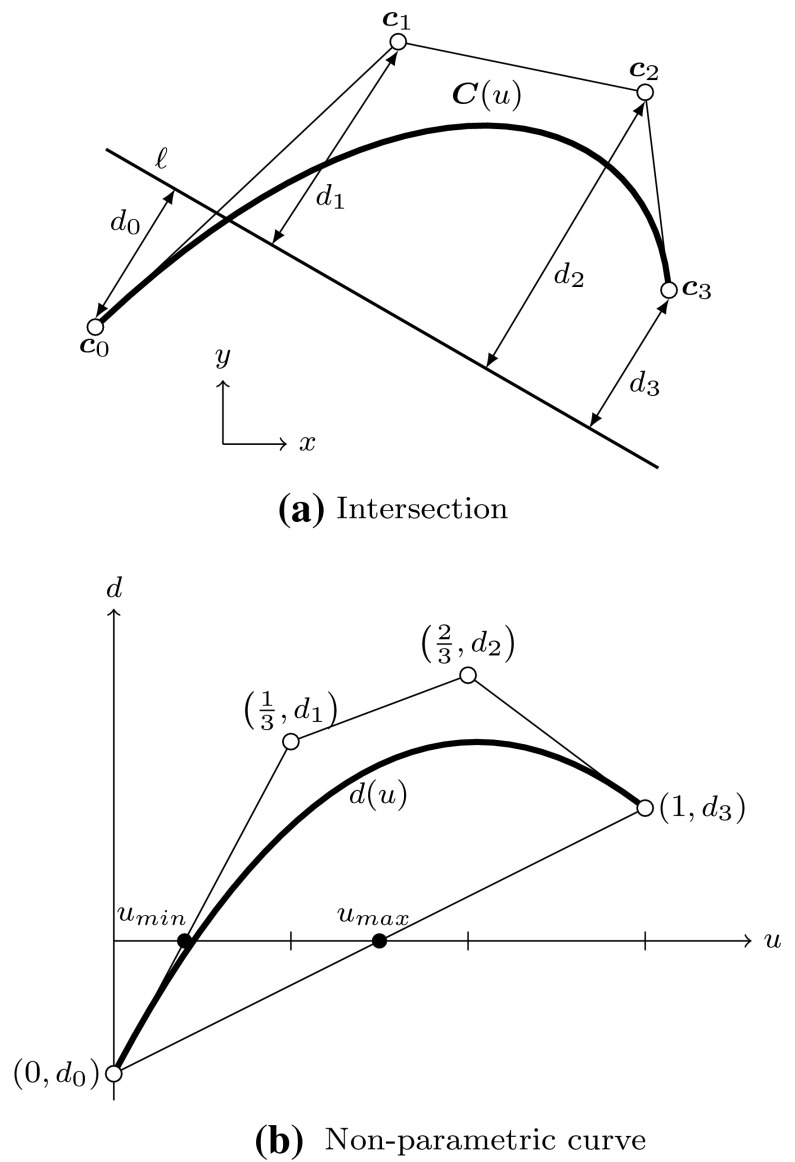
Bézier clipping: **a** intersection of a ray $\ell$ with a Bézier curve ${\varvec{C}}{(u)}$ and **b** the corresponding non-parametric Bézier curve which is used to determine the parameter range $[{u_{max}},\,{u_{min}}]$ that contains the intersection of ${\varvec{C}}{(u)}$ and $\ell$

The coefficients ${d_{i}}$ are the distances of the control points ${{\varvec{c}}_{i}}$ of the Bézier curve to the ray and ${B_{i,p}}$ are Bernstein polynomials of degree $p.$ Equation (52) can be represented as a non-parametric Bézier curve ${\tilde{{\varvec{C}}}}(u,\,d{(u)})$ where the values ${d_{i}}$ are related to their corresponding Greville abscissae, i.e., ${u_{i}}=\frac{i}{p}.$ The relationship of the original and non-parametric Bézier curve are depicted in Fig. 31.

The roots of ${\tilde{{\varvec{C}}}}(u,\,d{(u)})$ are equivalent to the parametric values $u$ at which $\ell$ intersects ${\varvec{C}}{(u)}.$ Hence, the convex hull of ${\tilde{{\varvec{C}}}}(u,\,d{(u)})$ can be used to identify regions where the objects do not intersect. To be precise, the minimal and maximal parametric values, i.e., ${u_{min}}$ and ${u_{max}},$ of the intersections of the convex hull with the $u$-axis splits the parameter space into three regions of which only one, i.e., ${u_{min}}\leqslant u\leqslant {u_{max}},$ has to be considered for the intersection with the ray. This region is extracted as a Bézier curve by means of knot insertion and the procedure is repeated until a certain tolerance is reached.

This technique can also be utilized to determine the intersection of two Bézier curves by iteratively clipping both objects [269]. Sherbrooke and Patrikalakis [280] developed a generalization of Bézier clipping that allows computing the roots of an *n*-dimensional system. The so-called *Projected-Polyhedron* method subdivides an object into Bézier segments and generates each side of its bounding boxes by projecting the control points onto different planes. Thus, only the convex hull of two-dimensional point sets has to be computed.

### Summary and Discussion

The previous parts of this section shed light on various aspects of trimmed NURBS in the context of CAD. On the basis of the discussion of surface intersection in Sect. 3.1, it can be concluded that there is no canonical way to derive trimming curves, but a wide range of different techniques to address this problem. Further, an exact representation of an intersection of two patches is not feasible in most cases. In fact, several distinct curves are usually used to specify a single intersection: a curve in model space and trimming curves in the parameter spaces of all patches involved. These curves are *independent* approximations of the actual intersection and there is no connection between them. This missing link makes it very difficult to transfer information from a trimmed patch to an adjacent one.

The necessity of approximation yields gaps and overlaps between intersecting patches. As a result, robustness problems arise for solid modeling as outlined in Sect. 3.2. Considerable effort has been devoted to derive consistent trimmed models. Still, the problem is unresolved and the absence of truly robust representations poses a demanding challenge, especially for downstream applications. Even within the field of CAGD, the replacement of trimmed surfaces by other representations may be needed. Tessellations are used in the context of rendering, for instance. In this particular case, the main reason is efficiency as discussed in Sect. 3.3. However, all other remodeling approaches presented in Sect. 3.4 are motivated by the flaws of trimmed models and the limitation of tensor product patches due to their four-sided nature. It should be emphasized that these schemes may yield watertight models, but there are certain tradeoffs. First of all, approximations are introduced at least in the vicinity of trimming curves. The number of control variables increases particularly if regular tensor product patches are used for the remodeling. Subdivision surfaces and T-splines are promising techniques, but may induce new problems like extraordinary vertices^8^ and linear dependence of the basis functions. In addition, they are designed for a specific surface type which may be an issue if a model consist of parts with different polynomial degree. Overall, it is apparent that there is no simple solution to the trimming problem.

Despite their difficulties, trimmed NURBS are *the* standard in engineering design and for the exchange of geometrical information in general. On the one hand, trimmed tensor product surfaces persist for historical reasons since they are a well-established technology, integrated in current CAD software. On the other hand, this representation distinguishes itself by its efficiency, precision, and simplicity. Trimming problems are hidden from the user who usually designs a model with the help of black box algorithms. Isogeometric analysis and adaptive manufacturing may lead to new developments in CAGD, but trimmed models are the state of the art and changes will certainly take time. It is not clear to the authors whether trimmed NURBS or other techniques like T-splines and subdivision surfaces will triumph in the future, but it is good to see the competition. At this juncture, however, trimmed NURBS seem to be the dominant technology of engineering design.

## Exchange Standards

At the beginning of this section, general considerations for exchanging data between different computer software systems is discussed. Next, the most popular neutral exchange standards, i.e., the Initial Graphics Exchange Specification (IGES) and the Standard for the Exchange of Product Model Data (STEP), are briefly introduced and compared. Finally, this section closes with some concluding remarks.

### General Considerations

In modern CAD systems, parameters and constraints govern the design of a model, rather than the definition of specific control points. Further essential components are local features and the construction history. All these various factors are referred to as *design intent* [162]. Each software has its own native data structure to keep track of the geometry, the topology, and the design intent of its models. Thus, a translation process is required when information is exchanged between systems with different native structures. The conversion of formats may seem like an easy task, but it is in fact very complicated. Usually, there is no direct mapping from one format to another. This holds true in particular for information related to the design intent since there are no canonical guidelines for its representation. Consequently, the exchange of the complete data of a CAD model between different systems is scarcely possible, especially when the systems are designed for different purposes. In most cases, only the geometric information of the final object is transferred.

This interoperability issue has been investigated in a study focusing on the US automotive supply chain [294]. The following possible solutions have been discussed: (i) standardization on a single system, (ii) point-to-point translation, and (iii) neutral format translation.

In case of a single system standardization the same native format is used for all processes, e.g., design and analysis. The main advantage is that the compatibility of the model data is assured since no translation is required. However, this approach implies the restriction to a single system. Consequently, every part has to be adjusted to the developments of the dominant application of the software. Most importantly, translation problems can arise even within one system due to different software versions—just imagine you would like to open a PowerPoint presentation created 10 years ago.

The basic idea of point-to-point translation is to convert a native format of a system directly to a native format of another one. This concept works reasonably well for unambiguous data exchange tasks. Unfortunately, it is not always clear how a given information should be translated so that it is properly interpreted in another native format. In addition, a high degree of vendor cooperation is necessary in order to develop a direct translator. Similar to the previous strategy, direct translators have to be rewritten for each new system or perhaps even for new versions of the same software.

Neutral format translation is based on a common neutral format for the exchange of (geometric) data. This approach enables an independent development of various tools working on the same model. The minimization of dependencies simplifies the maintenance of each software and eventually leads to robust implementations since a clean code is designed to do one thing well, as noted by Stroustrup^9^ [196]. Further, vendors are more willing to develop translators for neutral formats since it does not require the disclosure of proprietary code. This is beneficial since interpretation errors of the native format are most likely minimized when the conversion is provided by vendors themselves. An additional advantage of neutral formats is that they are ideally suited for long term storage of data. However, there are also a number of weaknesses. First of all, it is not possible to capture the design intent and thus, translation to a neutral format provides only a snapshot of the current geometric model. In general, every translation leads to loss of information and the quality of an exchanged model depends on the capability of the neutral format used.

In the context of isogeometric analysis, the minimal requirement is the accurate exchange of geometrical information of the final model. Topology is also essential to assess the connectivity between patches. The reconstruction of topological data based on edge comparison or related strategies is very cumbersome and extremely error-prone, especially in cases of trimmed geometries where edges only coincide within a certain tolerance as elaborated on in Sect. 3. The following two approaches are suggested: (i) direct extraction of topological data from CAD software by a point-to-point translation or (ii) using a neutral format that is able to cope with topological data. The former may be preferred if there is a cooperation with a CAD vendor and the developments focus on the specific product. However, neutral exchange formats will be discussed in the following because they are the most general and independent approaches. Despite their deficiencies, native formats seem to be the most sustainable solution.

### Neutral Format Translators

Concepts for a common data exchange format emerged in the 1970s. These attempts were borne by a variety of partners from industry, academia, and government [101]. Based on the initiative of the CAD user community, in particular General Electric and Boeing, vendors agreed to create an American national standard for CAD data exchange. The final result was the first version of IGES [209]. IGES provided the technical groundwork to a more involved exchange format, namely STEP.

#### IGES

The name of this neutral exchange format already reveals its original purpose [101]:

- *Initial* 10 to suggest that it would not replace the work of the American National Standards Institute.
- *Graphics* not geometry, to acknowledge that academics may come up with superior mathematical descriptions.
- *Exchange* to suggest that it would not dictate how vendors must implement their native database.
- *Specification* to indicate that it is *not* imposed to be a standard.

IGES provided an important and very practical first solution to the exchange problem, resulting in a file format that is implemented in almost every CAD system. Regarding current literature on isogeometric analysis, it seems that IGES is still the preferred choice when it comes to the extraction of geometric information. Here, we will try to disprove this notion.

According to Goldstein et al. [101] and the studies cited within, the shortcomings of IGES can by summarized as follows: (i) it contains several ways to capture the same information leading to ambiguous interpretation, (ii) loss of information during exchange, (iii) development without rigorous technical discipline, (iv) restriction of exchange capabilities due to the compliance with earlier IGES versions, (v) it was developed as a method to exchange engineering drawings, but not designed for more sophisticated product data, (vi) vendors implemented only portions of IGES, and (vii) there is no mechanism for testing the translators. In addition, IGES is a national standard which may lead to translation problems if other than US software is used. Most importantly, IGES is a *stagnant* exchange format. The last official version of IGES, i.e., version 5.3 [155], was published more than 20 years ago in 1996.

Although IGES continues to be deployed in industry, its main legacy is the disclosure of several weaknesses of the neutral exchange concept, thereby enhancing new emerging standards. The most notable one is STEP, which provides a broader, more robust standard for the exchange of data [101].

#### STEP

Since 1984 the International Organization for Standardization (ISO) has been working on a standard for the exchange of *product data* and its first parts were published in 1994 [232]. The objective of this development effort—one of the largest ever undertaken by ISO—is the complete and unambiguous definition of a product throughout its entire life cycle, which is independent of any computer system [264]. Hence, the corresponding standard includes the exchange of CAD data, yet its scope is much broader.

**Fig. 32:**
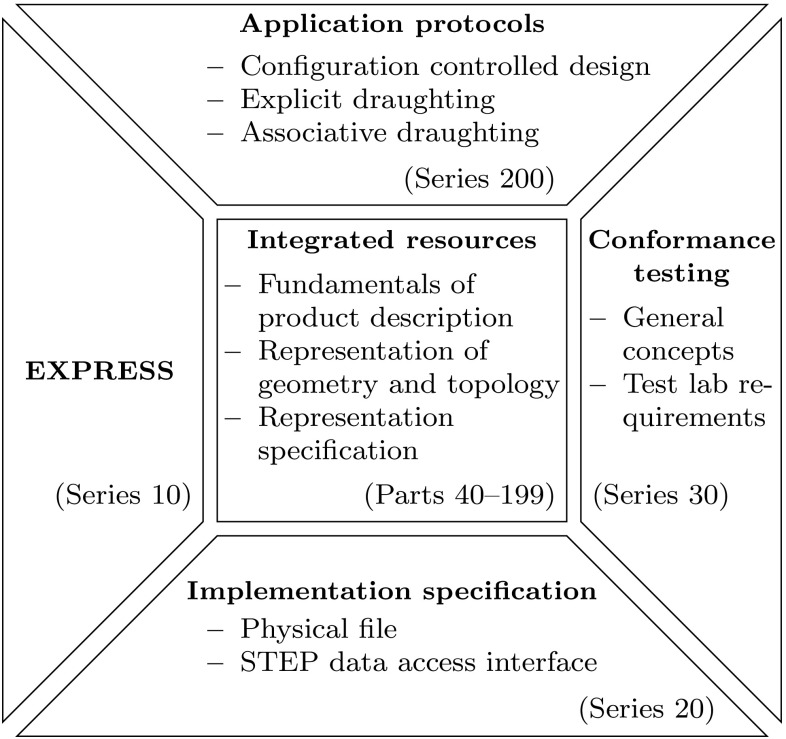
Structure of STEP (re-execution of the original diagram [177])

STEP is the informal term for the standard officially denoted as ISO 10303. It is organized by an accumulation of various *parts* unified by a set of fundamental principals. These parts are referred to as ISO 10303-xxx, where xxx is determined by the part number. Each of them is separately published and has to pass several development phases summarized in Table 1. Each part is associated with one of the following *series*: (i) description methods, (ii) implementation specifications, (iii) conformance testing, (iv) generic integrated resources, (v) application integrated resources, (vi) application protocols. Figure 32 gives an overview of these various components of STEP. The description is given by the common formal specification language EXPRESS (Series 10) defining data types, entities, rules, functions, and so on [274]. It is not a programming language, but has an object-oriented flavor. The transfer of data is defined by the implementation specifications (Series 20). The exchange by a neutral ACSII file is addressed in Part 21, “clear text encoding the exchange structure.” This STEP-file transfer is the most widely used data exchange form of STEP [264]. However, other approaches, like shared memory access, are covered by the series as well, see e.g., Part 22, “standard data access interface.” Conformance tests provide the verification requirements (Series 30).

**Table 1:** STEP stages

Stages	Names
PWI	Preliminary work item
NWI	New work item
AWI	Approved work item
WD	Working draft
CD	Committee draft
FCD	Final committee draft
DIS	Draft international standard
FDIS	Final draft international standard
PRF	Proof of new international standard
IS	International standard

The most fundamental components of STEP are the integrated resources. They contain generic information such as geometric data and display attributes (Series 40) as well as further elementary units that are specialized for certain application areas (Series 100). For example, Part 42, “geometric and topological representations,” focuses on the definition of geometric models in general, while Part 104, “finite element analysis,” is devoted to applications in the context of FEA. These parts provide the entities needed to build application protocols denoted as APs (Series 200). They are the link to the needs of industry and other users. Their purpose is to interpret the STEP data in the context of a specific application which may be part of one or more stages of a life cycle of a particular product. Part 209, “multidisciplinary analysis and design,” addresses engineering analysis. Each STEP application protocol is further subdivided into a set of conformation classes (CCs). These subsets must be completely implemented if a translator claims to be conform with the standard [233]. Hence, it is important to know what conformance classes are supported by a software system. This modular structure, with several APs and their CCs, may seem complex and daunting, but it gives users the necessary transparency of what can be expected of the data exchanged. Moreover, the complexity of the overall concept of STEP does not imply that it is difficult to use.

The downside of the broad scope of STEP is the large amount of detailed information which may seem overwhelming at first glance. In addition, ISO documents are not available for free, but can be purchased through the ISO homepage.^11^ There are, however, helpful resources to start with: the US Consortium called Product Data Exchange using STEP (*PDES, Inc.*) provides several resources^12^ like a handbook for a general introduction to STEP [264]. Further background articles are also released by the developers of *STEP tools, Inc.* 13 ISO permits EXPRESS listings to be distributed without copyright restrictions and several examples are given in the software’s archive.^14^

One of the advantages of STEP is that it is more than just a specification for exchanging geometric information. It provides a complete product data format allowing the integration of business and technical data of an object, from design to analysis, manufacturing, sales, and service [294]. STEP is perfectly aligned with the spirit of isogeometric analysis, i.e., unifying fields. The most important feature of STEP is its extensibility. Efforts have been made to include the design intent into STEP [162, 233, 234]. Particularly interesting for isogeometric analysis is the specification of volumetric NURBS and local refinement in the next versions of Part 42 and other parts [284].

#### Comparative Example

**Fig. 33:**
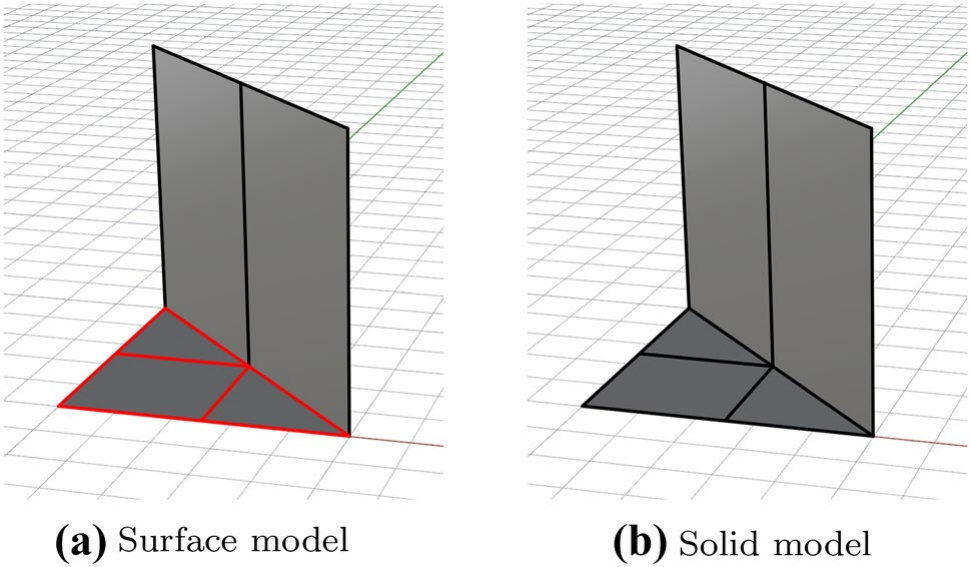
Design model with the same geometry but different topology: **a** two independent trimmed surfaces and **b** connected surfaces by a Boolean operation. In **a**, the isocurves of the separated surfaces are displayed in *black* and *red*, respectively. (Color figure online)

In order to demonstrate the representation of trimmed geometries in IGES and STEP, an example of two intersecting planes is considered. A square $[0,\,5]^{2}$ within the *xy*-plane is perpendicularly intersected along its diagonal by another plane surface as illustrated in Fig. 33. Thereby, the perpendicular patch is also trimmed into two halves by the square.

The model investigated has been constructed using the software Rhinoceros and the intersection has been computed in two different ways: using (i) the trim-command and (ii) the Boolean-command, respectively. Both schemes lead to the same geometry, yet the topology varies as indicated by the different highlighting of Fig. 33a and b. To be precise, the trim-command produces a *surface model* that consists of two independent trimmed surfaces, while the Boolean-command results in a *solid model* where the patches are connected. Both models have been exported to neutral exchange formats. The corresponding IGES and STEP files are provided in the Appendix. In the following, certain aspects of the exported files are discussed.

##### *Remark 3*

The default setting of the Rhinoceros export has been chosen, i.e., AP213AutomotiveDesign, for the STEP examples. However, the elements discussed in this section are not affected by this choice.

##### File Structure

**Fig. 34:**
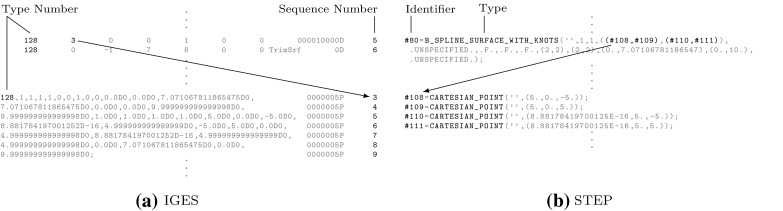
Entity connection of the exchange formats. Pointers are indicated by *arrows*. The examples have been extracted from Files 1 and 3 of the Appendix, respectively

The fixed ASCII file format^15^ of IGES is structured by the subsequent sections: Start (S), Global (G), Directory Entry (D), Parameter Data (P), and Termination (T). The letters in the brackets label these distinct parts and they are shown in column 73 of every file. The Directory Entry and the Parameter Data is specified by *entities* which are associated with a unique type number. Table 2 lists the entities used in this example. The Directory Entries provide attribute information for each entity in an IGES file. Each entry is fixed in size and is specified by 20 fields. The first field contains the entity type and the second one points to the first line of the related Parameter Data record. This connection is shown for a rational B-spline surface in Fig. 34a. The Parameter Data, on the other hand, is free-formatted and it consists of a sequence of integer and real numbers starting with the entity type number.

**Table 2:** Entity types of the IGES example

Numbers	Names	Information
126	Rational B-spline curve	Geometry
128	Rational B-spline surface	Geometry
141	Boundary entity	Topology
143	Bounded surface entity	Topology

**Fig. 35:**
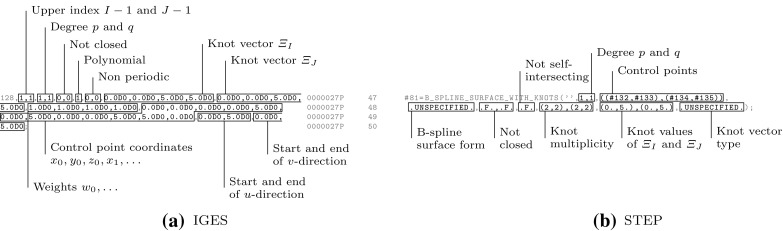
Descriptions of the surface model’s regular square patch. The B-spline surface data has been extracted from Files 1 and 3 of the Appendix, respectively

STEP files are easy to read since the language used is based on an English-like syntax [274]. In general, an accumulation of entities pointing to each other shapes the structure of the exchange data. Lines specifying entities begin with the symbol #, followed by the unique identifier of the corresponding object. This identifier is used to connect various entities with each other as shown in Fig. 34b. Besides pointers, an entity may consists of integers, real numbers, Booleans (.F./.T.), and enumeration flags (e.g., .UNSPECIFIED.).

##### Surfaces Representation

Both exchange formats provide the fundamental informations of B-spline patches, i.e., degree, knot vectors, and control points, together with auxiliary information. In case of IGES, a sequence of numbers separated by commas is used, while STEP additionally groups associated components using brackets. In Fig. 35, the representations of the regular square patch are compared. Note that knot vectors are specified by knot values with their multiplicity and that coordinates of control points are stored within its own entity in case of STEP. Hence, error-prone comparisons of floating point numbers are avoided.

Regarding trimmed surfaces, the following information is provided in both exchange formats: (i) the regular surface, (ii) the related loops of trimming curves, and (iii) their counterparts in model space. All this information can be found in a single IGES entity, i.e., 141. In particular, the fourth and fifth number within the sequence of this entity define the reference to the regular surface and the number of related curves, respectively. These numbers are followed by arrays of the size 4. The first value of an array refers to model space curves while the last value points to trimming curves. The total number of arrays is determined by the number of related curves.

**Fig. 36:**
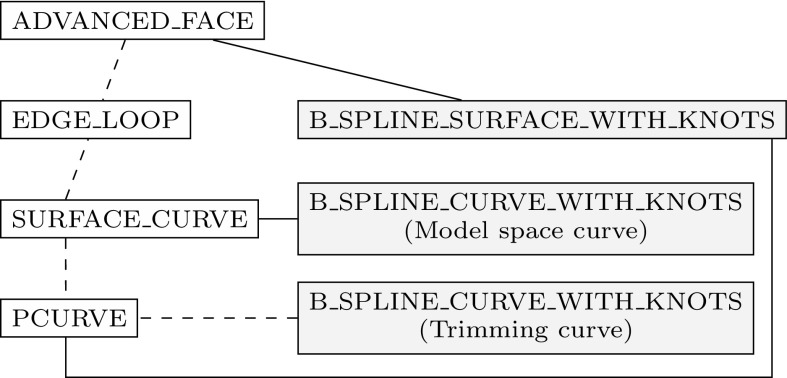
Graph related to a trimmed surface in STEP. Entities that provide geometrical information are highlighted in *gray*. Intermediate nodes may be skipped which is indicated by *dashed lines*

In case of STEP, the trimmed surface data is not coalesced in a single object, but it is embedded in a graph structure. Hence, the information is represented by various different entities which are linked together. The ADVANCED_FACE entity may be viewed as the starting point of the graph structure that specifies the trimmed patch. Figure 36 illustrates such a collection of entities. For the sake of clarity, some intermediate entities have been neglected as indicated by the dashed lines.

##### Topology

So far, the specification of certain parts of a model has been addressed. Here, the differences between the exchange formats regarding an object’s topological information is examined by comparing the output for the surface model and solid model shown in Fig. 33. The former is defined by two independent surfaces, while the latter is a single coherent manifold.

**Fig. 37:**
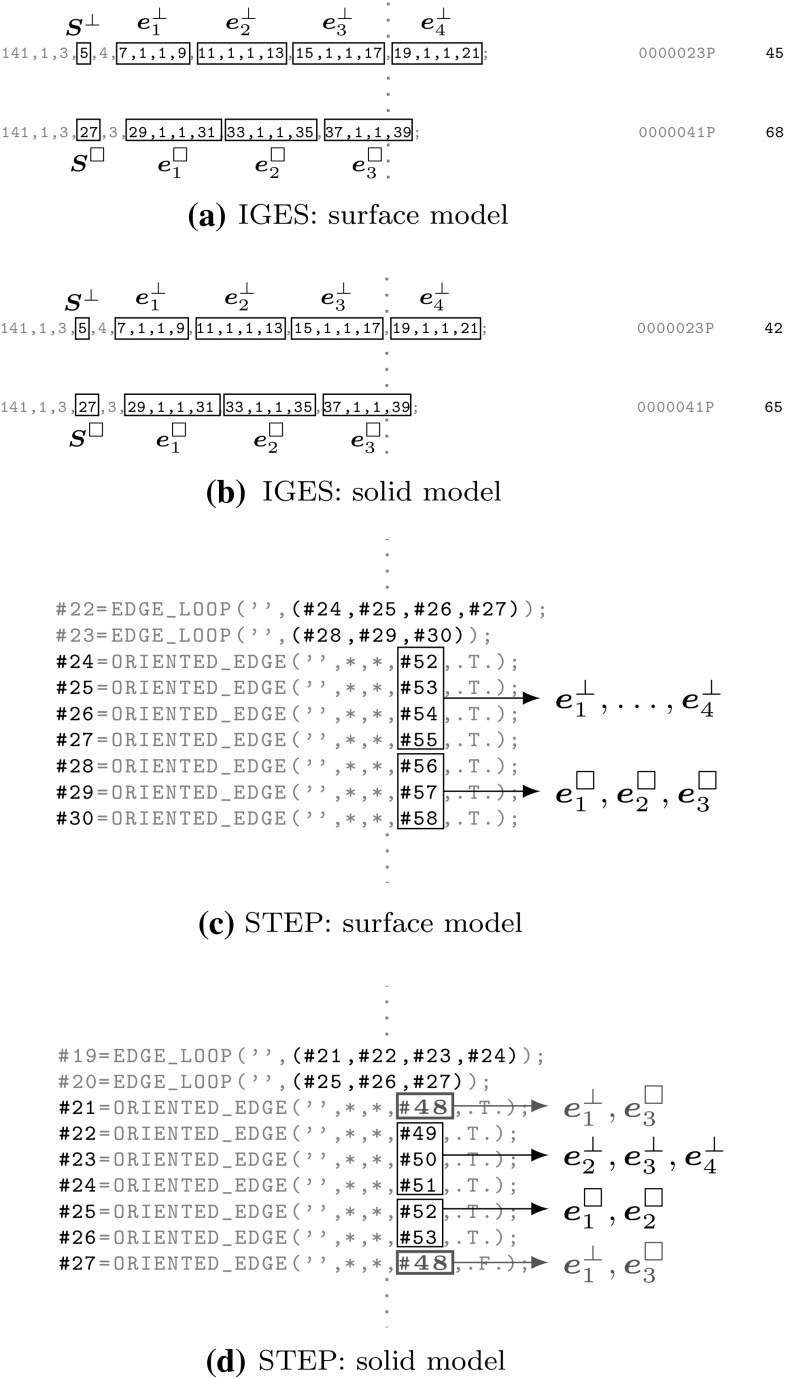
The STEP and IGES loop data of the model illustrated in Fig. 33. Entries referring to edges are labeled by ${{\varvec{e}}^{\square }_{i}}$ and ${{\varvec{e}}^{\perp }_{j}}.$ The index numbers are not consistent and thus, may differ from sub-figure to sub-figure. In **d**, the highlighted pointer, i.e., $\#48,$ refers to the edge where the two surfaces ${{\varvec{S}}^{\square }}$ and ${{\varvec{S}}^{\perp }}$ intersect

In the following the square patch in the *xy*-plane is denoted by ${{\varvec{S}}^{\square} }$ and the perpendicular patch is referred to as ${{\varvec{S}}^{\perp} }.$ The corresponding edges of the model are labeled ${{\varvec{e}}^{\square }_{i}}$ with $i=\{ 1, \ldots , 3 \}$ and ${{\varvec{e}}^{\perp }_{j}}$ with $j=\{ 1, \ldots , 4 \},$ respectively. The topology due to the STEP and IGES formats is compared in Fig. 37. To be precise, the provided edge loop data is shown. Further details are neglected for the sake of brevity, but the entire files can be found in the Appendix.

Comparing Fig. 37a and b shows that IGES yields the *same* output for both models. In other words, the different topologies of them are not recognized. Note that the only values that differ are the sequence numbers of the entities which are completely independent from the actual object. In fact, the topological connection of ${{\varvec{S}}^{\square} }$ and ${{\varvec{S}}^{\perp} }$ is lost in case of the solid model, despite the simplicity of the example. That the solid model has been properly constructed is proven by the STEP output shown in Fig. 37d where the edges ${{\varvec{e}}^{\perp }_{1}}$ and ${{\varvec{e}}^{\square }_{3}}$ are joined in a single reference, i.e., $\#48.$ The STEP data related to the surface model is illustrated in Fig. 37c.

### Summary and Discussion

In Sect. 4.1, the problem of exchanging data between different systems is outlined from a general point of view. It is argued that the use of neutral exchange standards is the most comprehensive way for this task. Nevertheless, every mapping from one system to another may cause problems, especially with respect to the design intent of a model where no canonical representation exists. There is often no one-to-one translation from one format to another, which leaves room for (mis)interpretation. As a result, exchange is usually restricted to snapshots of an object’s geometry. The transfer of topology data is also possible if (i) the design model is properly constructed as a coherent solid model and (ii) the neutral format is able to capture this information. It is apparent that the capability of the neutral standard applied is essential for the quality and success of the exchange. This has been demonstrated by a simple example given in Sect. 4.2.3 where IGES does not export the topology correctly.

STEP should generally be preferred as a neutral exchange format. According to Tassey et al. [294], STEP is superior to other translators because it

- addresses many types of data,
- incorporates a superset of elements common to all systems,
- supports special application needs, and
- provides for international exchanges.

In their paper, several studies are discussed in which STEP excels with respect to the quality of exchanging data of industrial examples. In addition, this standard is constantly developed and improved, e.g., by its enhancements for isogeometric analysis [284].

Theoretically, the broad scope and modular structure of STEP provides coverage of various application domains which are indicated by the application protocols and their conformance classes. However, this functionality has to be supported by the CAD vendors. Most vendors have chosen to implement only certain parts of STEP, i.e., some conformance classes of AP 203 and AP 214 [264]. It is not surprising that vendors seem to show little interest in neutral exchange formats, since their implementation slows down the development of the actual software and users become more independent from their products. Hence, it is likely that neutral file formats will always provide less information than the original model. Translation errors may be avoided if the needed data is extracted directly from the native format, but this requires vendor interaction and the restriction to a single software. This alternative is not very sustainable since a native format may become obsolete after a new software version is released.

## Isogeometric Analysis of Trimmed Geometries

Isogeometric analysis of trimmed NURBS is an important research area, simply due to the omnipresence of such geometry representations. Integration of design and analysis can only be achieved if the simulation is able to cope with CAGD models that are actually used in the design process. Moreover, sound treatment of trimmed solid models is also an essential step for the derivation of volumetric representations.

Current attempts to integrate trimmed geometries into isogeometric analysis may be classified as *global* and *local* approaches. The latter uses the parameter space of the trimmed patch as background parameterization and the trimming curves determine the domain of interest, i.e., ${{\mathcal {A}}^{\text{v}} },$ for the analysis. Knot spans that are cut by trimming curves require special attention during the simulation. In that sense, local approaches are closely related to fictitious domain methods,^16^ see e.g., [124, 239, 252, 259]. Consequently, similar tasks have to be undertaken: (i) detection of trimmed elements, (ii) application of special integration schemes in these elements, and (iii) stabilization of the trimmed basis. CAGD models are not modified but the analysis has to deal with all the related robustness issues pointed out in Sect. 3.2.2. Global reconstruction, on the other hand, substitutes a trimmed surface by one or several regular patches which can be analyzed with regular integration rules. In other words, it is endeavored to fix the design model, before it is used in the downstream application, e.g., the simulation. These approaches are similar to remodeling schemes in CAGD presented in Sect. 3.4.1. Isogeometric analysis of subdivision surfaces, e.g., [59, 248, 309], and T-splines, e.g., [22, 262, 263, 321], may be included into the class of global reconstruction techniques. However, the discussion of the analysis of these representations is beyond the scope of this review.

Coupling of multiple patches is another issue that has to be addressed. Adjacent patches usually have non-matching parameterizations and a robust treatment of tolerances is required to link the degrees of freedom along an intersection due to the gaps between trimmed surfaces and the missing link between their trimming curves. Local approach have to deal with the issue directly during the analysis, while global approach apply this crucial step beforehand during the remodeling phase. The coupling itself is usually performed by a weak coupling technique. Alternatively, some global schemes try to establish matching parameterizations during the reconstruction procedure. This allows an explicit coupling of patches and a better control of the continuity between adjacent patches [149]. It is also noteworthy that the coupling procedure may be neglected in certain simulation methods. For example, the boundary element method and the Nyström method do not require certain continuity between elements or patches, see e.g., [218, 223, 225, 319].

The following approaches for analyzing trimmed geometries have been applied to finite element and boundary element methods. The former focuses on shell analysis while the latter is used for volumetric B-Rep models. However, the basic concepts are not restricted to a specific simulation type since in both cases the treatment of trimmed surfaces is in the focus. It will be highlighted if a certain part explicitly applies for a specific simulation method.

The overview begins with a short historical note, which, to the best of the authors’ knowledge is the first direct simulation with trimmed patches. Afterwards, the current state of research is reviewed in the Sects. 5.2 and 5.3 addressing global and local approaches, respectively.

### The First Analysis of Trimmed Models

It is fascinating that the analysis of trimmed patches goes back to the genesis of trimmed patch formulations. In fact, Casale et al. [48, 50] presented an analysis of such geometries a few years after they had suggested one of the first trimmed solid model formulations [47, 49]. In particular, *trimmed patch boundary elements* had been proposed.

The basic idea of their approach is to employ the trimmed patch for the geometrical representation and to define an independent Lagrange interpolation over the tensor product surface for the description of the physical variables. This additional basis does not take the trimming curves into account. Thus, the nodes of the Lagrange interpolation may lie inside or outside the trimmed domain. This is emphasized by using the term *virtual nodes*. The analysis is performed by means of a collocated boundary element formulation, see e.g., [96], where all Lagrange nodes contribute to the system matrix. If a node is outside of the trimmed domain, the jump term coefficient^17^ of the boundary integral equation is set to 1 since the node is not part of the object’s boundary. Numerical integration is performed over a triangulation of the trimmed domain. These triangles are used to define integration regions only and do not contribute any degrees of freedom.

This concept has various deficiencies, but it consists of features that can be found in current approaches as well. For example, defining the geometrical mapping by the trimmed parameter space, but the physical fields by a different (spline) basis is employed in some global techniques presented later. There are also similarities to local schemes since the trimmed domain is treated like a background parameterization leading to special considerations regarding numerical integration and points that are not within the domain. Furthermore, the motivation for the application of the boundary element method was the same as today in isogeometric analysis, i.e., the potential of a direct analysis of B-Rep models without the need of generating a volumetric discretization.

### Global Approaches

Global reconstruction schemes decompose trimmed surfaces into regular patches. The general concept is the same as presented in Sect. 3.4.1 in the context of CAGD. The distinguishing aspect is that the following strategies are aimed to provide analysis-suitable models.

#### Reconstruction by Ruled Surfaces

Trimming curves ${{\varvec{C}}^{t}}$ may be used to define a mapping ${{\mathcal {X}}_{t}}$ such that a regular tensor product basis specifies the valid area ${{\mathcal {A}}^{\text{v}}}$ of the corresponding trimmed patch, as proposed by Beer et al. [25]. To be precise, ${{\mathcal {X}}_{t}}$ is given by a linear interpolation between two opposing ${{\varvec{C}}^{t}_{i}},\,i=\{1,\,2\}.$ The geometrical mapping to the model space is performed by the original trimmed patch, hence the approach is also referred to as *double mapping method*.

The following assumptions are made for the sake of notational simplicity. Firstly, the regular basis functions are defined over a unit square, i.e., $s,\,t\in [0,\,1 ].$ In addition, it is assumed that both trimming curves are specified within the same parameter range ${\tilde{u}}\in [a,\,b ].$ Based on that, the intrinsic coordinate ${\tilde{u}}$ can be linked to the boundaries of the regular basis at $t=0$ and $t=1$ by the coordinate transformations $f(s)$ and $g(s).$ They are given by

53 $$\begin{aligned} {\tilde{u}}= f(s)&= a + s(b-a), \end{aligned}$$

and

54 $$\begin{aligned} {\tilde{u}}= g(s)&= b + s(a-b). \end{aligned}$$

These equations traverse the interval of ${\tilde{u}}$ in opposite directions, e.g., $f(0)=g(1)=a,$ since one of the trimming curves has to be evaluated in reverse order. Finally, ${{\mathcal {X}}_{t}}$ is determined by

55 $$\begin{aligned} {\varvec{S}}^{{\mathcal {A}}^{\text{v}}}(s,\,t) = (1-t){{\varvec{C}}^{t}_{1}}(f(s)) + t{{\varvec{C}}^{t}_{2}}(g(s)). \end{aligned}$$

**Fig. 38:**
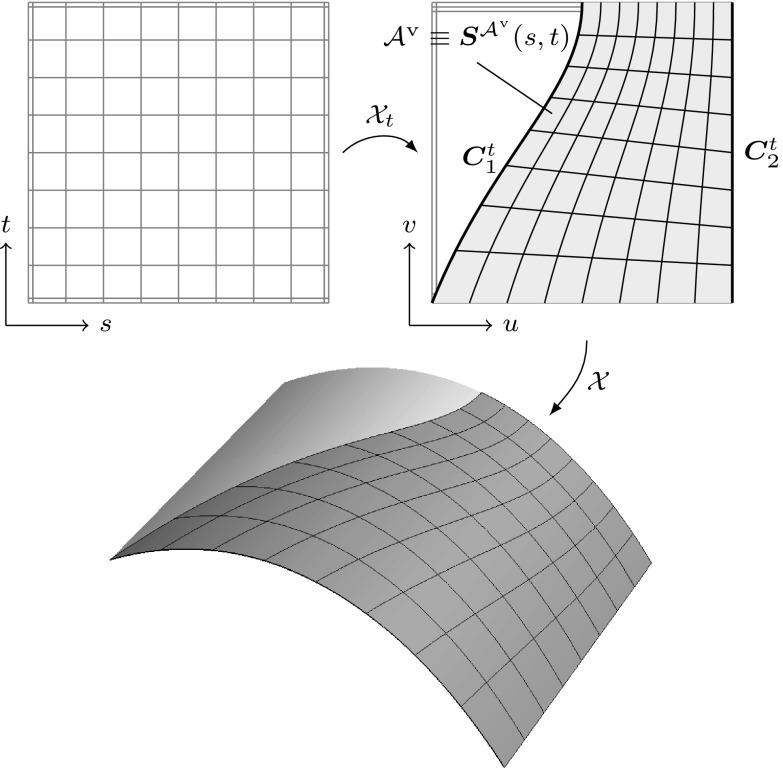
Double mapping scheme to fit a regular tensor product surface to a trimmed patch. The first mapping ${{\mathcal {X}}_{t}}$ specifies the transformation to the valid area ${{\mathcal {A}}^{\text{v}} }$ of the trimmed parameter space, while the geometric mapping is denoted by ${\mathcal {X}}.$ The trimming curves ${{\varvec{C}}^{t}_{i}}({\tilde{u}}),\, i=\{1,\,2\},$ are illustrated by *thick lines*

From a CAGD point of view, the mapping (55) is equivalent to the one of a ruled surface (26). The main difference is that the ruled surface is defined in the parameter space in this case. The geometric mapping ${\mathcal {X}},$ however, is still performed by the trimmed patch. Figure 38 summarizes the concept of the double mapping approach.

**Fig. 39:**
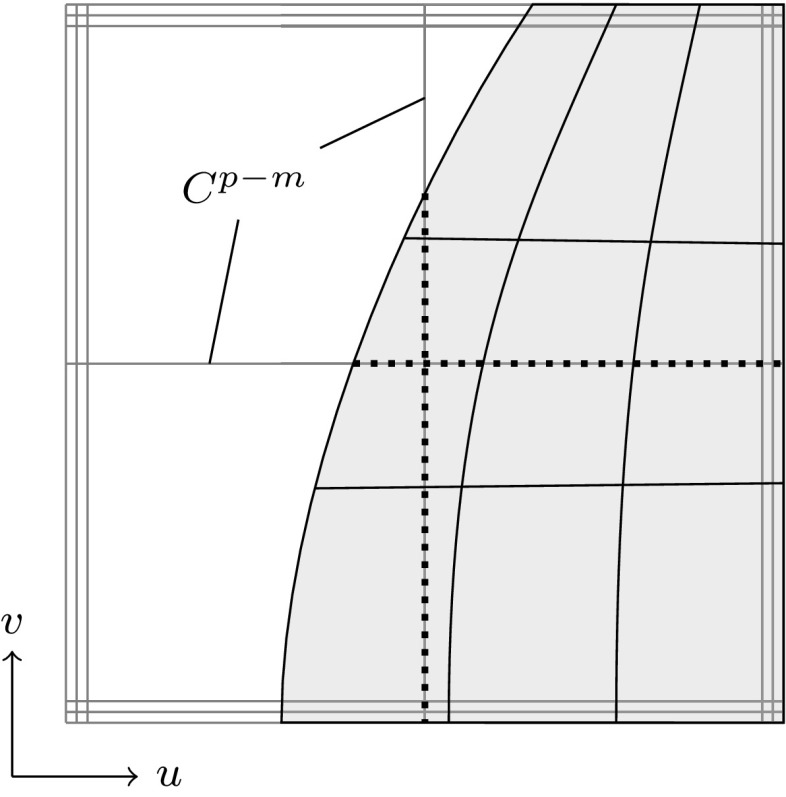
Double mapping method for a B-spline patch. The *dotted lines* indicate parameter curves that are not ${C^{\infty}}$-continuity within ${{\mathcal {A}}^{\text{v}} }$

The main advantage of this approach is its simplicity and ease of implementation. However, there are various restrictions: first of all, the assumption that ${{\mathcal {A}}^{\text{v}} }$ is governed by two opposing trimming curves limits the application to very specific trimming situations. Furthermore, the four-sided nature of ${{\mathcal {A}}^{\text{v}} }$ is implied. Consequently, trimmed patches with more complex topology have to be decomposed by an additional preprocessing step. There is no control over the quality of the parameterization due to the mapping ${{\mathcal {X}}_{t}}.$ Elements may become very distorted depending on the position of the trimming curves ${{\varvec{C}}^{t}_{i}}.$ Such a situation occurs for a triangular-shaped ${{\mathcal {A}}^{\text{v}} },$ see Fig. 9. Since the parameterization is completely independent of the basis functions of the trimmed parameter space, the double mapping method works well for Bézier patches. An integration issue arises as soon as B-spline patches are considered. The problem is depict in Fig. 39. Note that the parameter lines of the geometry representation propagate through the elements defined by the mapped regular parameterization. Thus, integration of the regular elements is not performed over a ${C^{\infty }}$-continuous region. In order to get a proper distribution of quadrature points, the elements must be subdivided along the non-smooth edges. The specification of such regions is not straightforward. To conclude, the double mapping method is a simple solution for (Bézier) patches which had been trimmed during the design process, at least for ones that can be represented by a regular patch.

#### Reconstruction by Coons Patches

A natural extension of the previous method is to define the mapping ${{\mathcal {X}}_{t}}$ to a trimmed parameter space by means of Coons patches. In contrast to the ruled surface interpolation (55), ${{\mathcal {X}}_{t}}$ takes four boundary curves into account. Randrianarivony [237, 238] developed such an approach, which has been applied to wavelet Galerkin BEM in collaboration with Harbrecht [113]. Although they do not focus on isogeometric analysis per se, most of their techniques can be directly utilized: (i) decomposition of ${{\mathcal {A}}^{\text{v}}}$ into several four-sided patches, (ii) identification if ${{\mathcal {X}}_{t}}$ is a diffeomorphism,^18^ and (iii) construction of matching parameterizations of adjacent patches.

**Fig. 40:**
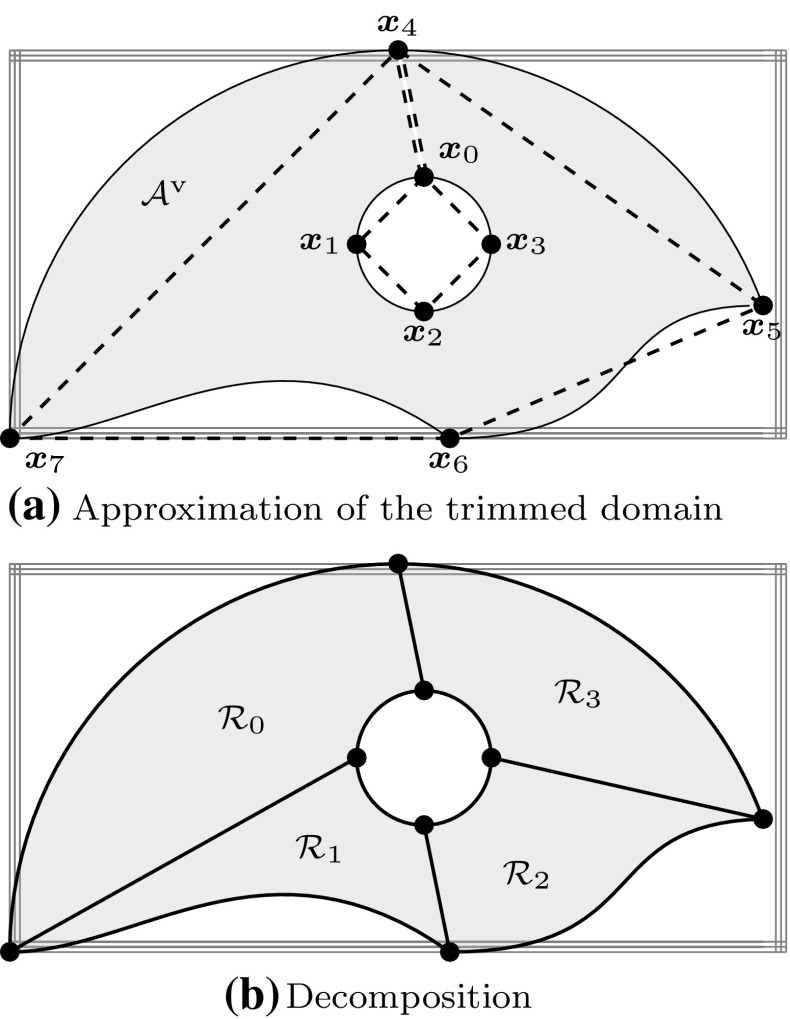
Decomposing of a trimmed domain ${{\mathcal {A}}^{\text{v}} }$ into **b** regular four-sided patches ${{\mathcal {R}}_{i}}.$ In **a**, the trimming curves are continuous whereas the linear approximation is illustrated by *dashed lines*. Further, vertex ${{\varvec{x}}_{0}}$ and ${{\varvec{x}}_{4}}$ are connected by a double edge

**Fig. 41:**
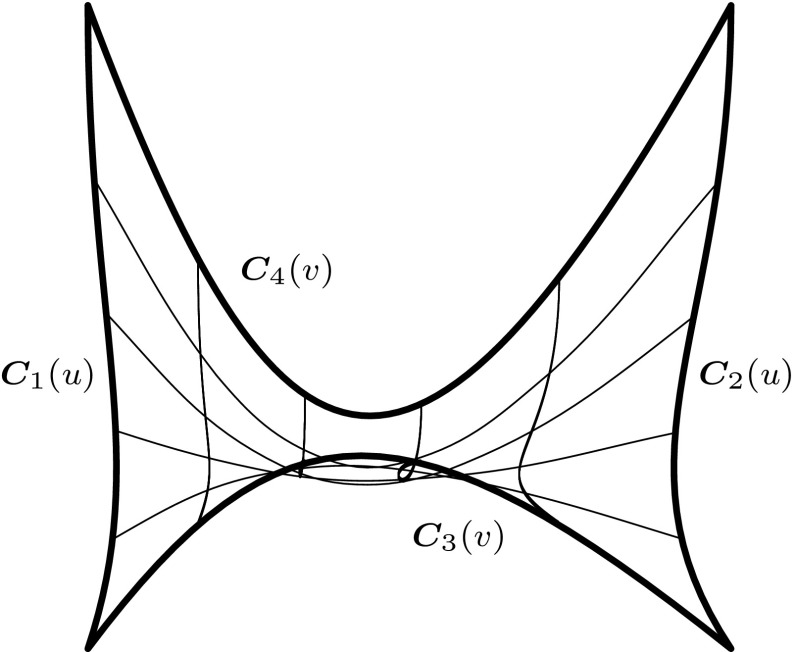
Example of a planar non-regular Coons patch where isocurves overlap

The first step of the decomposition procedure is to substitute the trimming curves ${{\varvec{C}}^{t}}$ of each patch by a linear approximation ${{\varvec{C}}^{l}}.$ The vertices ${\varvec{x}}$ of ${{\varvec{C}}^{l}}$ are located along ${{\varvec{C}}^{t}}$ as illustrated in Fig. 40a. ${{\varvec{C}}^{l}}$ should be as coarse as possible since the number of vertices determines the number of patches that decompose ${{\mathcal {A}}^{\text{v}}}.$ As initial approximation, the endpoints of the trimming curves may be used. However, ${\varvec{C}}^{l}$ has to be fine enough to resolve the topology of the trimmed patch, e.g., lines of exterior loops may not intersect ones of interior loops. In order to get a single polygon representing ${\mathcal {A}}^{\text{v}},$ interior loops are connected to exterior loops by so-called double edges. The vertices of the resulting polygon are basis for the decomposition of ${\mathcal {A}}^{\text{v}}$ into a set of quadrilaterals ${\mathcal {R}}.$ Therefore, it is important that the total number of vertices ${\varvec{x}}$ is even. In the next step, the straight boundary curves of ${\varvec{C}}^{l}$ are replaced by the complementary portions of ${\varvec{C}}^{t}.$ An example of a decomposition is shown in Fig. 40b. Due to this procedure, the following problems may arise. The most obvious one is that the curved boundary may intersect an internal edge. In addition, sharp corners become degenerated points if the corresponding ${\varvec{x}}$ is smoother than $C^{0}.$ As a result, no diffeomorphism for this region can be found [237]. Finally, it is not assured that a Coons patch interpolation is regular. Such problems arise particularly in case of non-convex domains. An example of a non-regular Coons patch where the parametric lines of the surface overspill is shown in Fig. 41. A remedy to the mentioned issues is local refinement of ${\varvec{C}}^{l}$ or the affected ${\mathcal {R}}_{i}.$ The detection of the first two problems is straightforward, but determination of a Coons patch’s regularity requires a more detailed discussion.

The following identification procedure assumes that the Coons patch interpolation (35) is planar and described by boundary curves given in Bézier form, i.e.,

56 $$\begin{aligned} {\varvec{C}}_{i}(u)&= \sum _{k=0}^{p} B_{k,p}(u) {\varvec{c}}^i_{k},\quad i=1,\,2,\quad u\in [0,\,1],\end{aligned}$$

57 $$\begin{aligned} {\varvec{C}}_{j}(v)&= \sum _{k=0}^{p} B_{k,p}(v) {\varvec{c}}^j_{k},\quad j=3,\,4,\quad v\in [0,\,1]. \end{aligned}$$

The interpolation function $f_{1}$ is also described as a Bézier polynomial

58 $$\begin{aligned} f_{1}(s)= \sum _{k=0}^{p}B_{k,p}(s) \psi _{k} =1-f_{0}(s),\quad s\in [0,\,1]. \end{aligned}$$

For the determination of the regularity, the factors $\mu ,\,\tau ,$ and $\alpha$ are specified by

59 $$\begin{aligned} \mu&:=\max \left\{ |f_{1}^\prime (s)|{\text {:}}\,s\in [0,\,1]\right\} ,\end{aligned}$$

60 $$\begin{aligned} \tau&:=\min \left\{ \tau _{k\ell }^{ij}\right\} ,\quad k,\,\ell =0,\ldots ,p, \end{aligned}$$

61 $$\begin{aligned} \alpha&:=\max \left\{ \alpha _{1},\,\alpha _{2}\right\} . \end{aligned}$$

The indices $i$ and $j$ are defined as above and the factors in Eqs. (60) and (61) are determined by

62 $$\begin{aligned} \tau _{k\ell }^{ij}&:= p^{2}\text {det}\left[ {\varvec{c}}^{i}_{k+1}-{\varvec{c}}^{i}_{k},\,{\varvec{c}}^{j}_{\ell +1}-{\varvec{c}}^{j}_{\ell } \right] , \end{aligned}$$

63 $$\begin{aligned} \alpha _{1}&:= \max _{k=0,\ldots ,p}\left\{ \mu ||\left( {\varvec{c}}^4_{k}-{\varvec{c}}^3_{k} \right) + \psi _{k} \hat{c} +\left( {\varvec{c}}^1_{0}-{\varvec{c}}^1_{p} \right) ||\right\} ,\end{aligned}$$

64 $$\begin{aligned} \alpha _{2}&:= \max _{k=0,\ldots ,p}\left\{ \mu ||\left( {\varvec{c}}^2_{k}-{\varvec{c}}^1_{k} \right) + \psi _{k} \hat{c} +\left( {\varvec{c}}^1_{0}-{\varvec{c}}^2_{0} \right) ||\right\} , \end{aligned}$$

with ${\hat{c}}=({\varvec{c}}^2_{0}-{\varvec{c}}^2_{p}+{\varvec{c}}^1_{p}-{\varvec{c}}^1_{0}).$ Finally, the constant $\beta$ is defined such that

65 $$\begin{aligned}&p\left\Vert\psi _{k} \left( {\varvec{c}}^2_{\ell +1}- {\varvec{c}}^2_{\ell } + {\varvec{c}}^1_{\ell }- {\varvec{c}}^1_{\ell +1} \right) + \left( {\varvec{c}}^1_{\ell +1}- {\varvec{c}}^1_{\ell } \right) \right\Vert\le \beta , \end{aligned}$$

66 $$\begin{aligned}&p\left\Vert\psi _{k} \left( {\varvec{c}}^4_{\ell +1}- {\varvec{c}}^4_{\ell } + {\varvec{c}}^3_{\ell }- {\varvec{c}}^3_{\ell +1} \right) + \left( {\varvec{c}}^3_{\ell +1}- {\varvec{c}}^3_{\ell } \right) \right\Vert\le \beta , \end{aligned}$$

for all $\ell =0,\ldots ,p-1$ and $k=0,\ldots ,p.$ Based on these definitions, the Coons patch mapping is regular if

67 $$\begin{aligned} 2\alpha \beta +\alpha ^{2}<\tau&&\,\text {and}\,&&\tau>0. \end{aligned}$$

These are only sufficient conditions. If they are not fulfilled a subdivision procedure is employed. Therefore, another sufficient condition is derived. Assuming that a Coons patch is represented as Bézier surface with control points ${\varvec{c}}^{C}_{i,j},$ the Jacobian can also be described as a Bézier function of degree $2p$ with the control points

68 $$\begin{aligned} {\varvec{c}}^{J}_{m,n} = \sum _{ \begin{array}{c} i+k=m\\ j+\ell =n \end{array} } C(i,\,j,\,k,\,\ell ) \frac{\left( {\begin{array}{c}p\\ i\end{array}}\right) \left( {\begin{array}{c}p\\ k\end{array}}\right) }{\left( {\begin{array}{c}2p\\ i+k\end{array}}\right) } \frac{\left( {\begin{array}{c}p\\ j\end{array}}\right) \left( {\begin{array}{c}p\\ \ell \end{array}}\right) }{\left( {\begin{array}{c}2p\\ j+\ell \end{array}}\right) }, \end{aligned}$$

with $m,\,n=0,\ldots ,2p$ and the coefficients

69 $$\begin{aligned} C(i,\,j,\,k,\,\ell )&:=\frac{\ell }{p} \left[ \frac{i}{p} D(i-1,\,j,\,k,\,\ell -1) \right. \nonumber \\&\quad +\left. \left( 1-\frac{i}{p}\right) D(i,\,j,\,k,\,\ell -1) \right] \nonumber \\&\quad +\left( 1-\frac{\ell }{p}\right) \left[ \frac{i}{p} D(i-1,\,j,\,k,\,\ell ) \right. \nonumber \\&\quad +\left. \left( 1-\frac{i}{p}\right) D(i,\,j,\,k,\,\ell ) \right] , \end{aligned}$$

where

70 $$\begin{aligned} D(i,\,j,\,k,\,\ell )&:= p^{2} \text {det}\left[ {\varvec{c}}^{C}_{i+1,j}-{\varvec{c}}^{C}_{i,j},\,{\varvec{c}}^{C}_{k,\ell +1}-{\varvec{c}}^{C}_{k,\ell } \right] . \end{aligned}$$

If the coefficients ${\varvec{c}}^{J}_{m,n}$ have the same sign the Coons patch mapping is a diffeomorphism. In case of unequal signs, the patch is adaptively subdivided and for each sub-patch the coefficients ${\varvec{c}}^{J_{sub}}_{m,n}$ are computed. The procedure stops as soon as the signs of ${\varvec{c}}^{J_{sub}}_{m,n}$ do not change within every sub-patch. The overall Coons patch is not regular, if it consists of sub-patches with different signs. It is emphasized that the subdivision is only performed to determine the regularity of the mapping. The corresponding proofs and more information can be found in [113, 237].

**Fig. 42:**
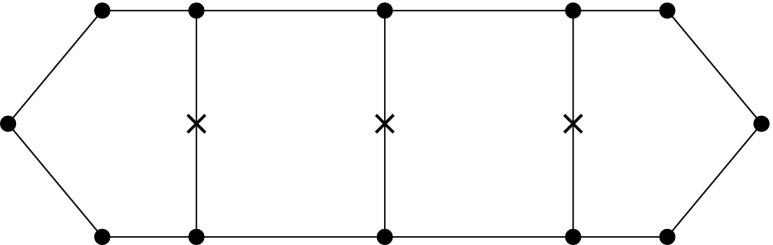
Converting a pair of odd faces to even ones. *Circles* indicate the initial vertices of the faces and *crosses* mark those vertices that had been added to obtain faces with an even number of vertices

In case of multiple patches, a matching parameterization between adjacent surfaces is sought. The connectivity is described by a graph. During the decomposition procedure, the polygon vertices of the adjacent patches ${\varvec{S}}_{i}$ and ${\varvec{S}}_{j}$ are computed so that

71 $$\begin{aligned} {\mathcal {X}}_{i}({\varvec{x}}^{i}_{k})={\mathcal {X}}_{j}({\varvec{x}}^{j}_{\ell }), \end{aligned}$$

where ${\varvec{x}}^{i}_{k}$ and ${\varvec{x}}^{j}_{\ell }$ are the vertices along the common edge and ${\mathcal {X}}$ denotes the geometrical mapping (23). If a vertex ${\varvec{x}}^{i}_{k}$ is added to obtain an even-numbered approximation ${\varvec{C}}^l,$ a corresponding ${\varvec{x}}^{j}_{\ell }$ needs to be added in the adjacent patch. Thus, odd faces can be converted to even ones only in pairs as illustrated in Fig. 42. It should be noted that the inserted vertices propagate through faces which are even already. In order to minimize the affected faces, the shortest path connecting two faces is computed by the application of Dijkstra’s algorithm [72] to the connectivity graph. In addition to matching vertices, trimming curves ${\varvec{C}}^{t}\in [a,\,b]$ are parameterized by means of the chord length of the corresponding intersection curve in model space. The trimming curve segment of quadrilaterals ${\mathcal {R}}_{i}$ is initially defined by ${\bar{\varvec{C}}}^{t}_{i}(t\cdot a_{i}+(1-t)b_{i})\in [a_{i},\,b_{i}]\subset [a,\,b].$ The new representation ${\hat{\varvec{C}}}^{t}_{i}$ is given by

72 $$\begin{aligned} {\hat{\varvec{C}}}^{t}_{i}:={\bar{\varvec{C}}}^{t}_{i} \circ \phi _{i}, \quad \phi _{i}=\left( \lambda _{i}\right) ^{-1}, \end{aligned}$$

with $\phi _{i}$ denoting the inverse of the length function

73 $$\begin{aligned} \lambda _{i} (t):=\int _{a}^{t}\left\Vert \frac{d{({\mathcal {X}}\circ {\bar{\varvec{C}}}^{t}_{i})}}{d{t}} (\theta ) \right\Vert \;\text {d}\theta . \end{aligned}$$

Hence, the images of the trimming curve ${\hat{\varvec{C}}}^{t}$ of adjacent patches match at the same parametric values, i.e., the same chord length. This procedure is applied before the Coons patch construction. For details on the computation of the reparameterization the interested reader is referred to [238].

In contrast to the approach described in the previous subsection, this reconstruction scheme addresses the partitioning of trimmed domains into several four-sided regions, the regularity of these regions, and the connection of adjacent patches. Yet, some aspects are unresolved. For instance, the compatibility condition (71) implies that trimming curves coincide which is usually not the case as discussed in Sect. 3. Since the trimming curves do not describe the same curve in model space, the chord length parameterization may lead to diverse results. Thus, a robust implementation is required that treats the tolerances involved. As pointed out in Randrianarivony’s thesis [237], the reconstruction algorithm might fail if the inaccuracies of CAGD models are too large. User interaction is required to find an adequate tolerance threshold. IGES data has been used as exchange format in the related publications, which makes the tolerance treatment even more delicate since the topology of the objects has to be reconstructed as elaborated in Sect. 4. Finally, the problem of integrating over $C^{\infty }$-continuous regions in case of B-spline patches is not addressed.

#### Reconstruction by Isocurves

Recently, Urick [298] presented a reconstruction approach based on isocurves (25). In contrast to the previous schemes, the trimmed patch is replaced by a new parameterization and a new set of control points. The overall procedure consists of several steps including (i) topology detection, (ii) parameter space analysis and determination of knot vector superset, (iii) reparameterization of trimmed parameter spaces, (iv) computation of corresponding control points, and (v) the treatment of multiple trimming curves.

In order to identify the topology of the trimmed domain ${\mathcal {A}}^{\text{v}},$ characteristic points ${\varvec{x}}$ of the trimming curves ${\varvec{C}}^{t}$ are determined. The points considered are summarized in Table 3.

**Table 3:** Characteristic points ${\varvec{x}}$ of a trimming curve

Types	Description
0	End points, kinks, and cusps
1	Slope relative to $u$ is 1 or −1
2	Slope relative to $u$ is 0
3	Slope relative to $u$ is $\infty$

**Fig. 43:**
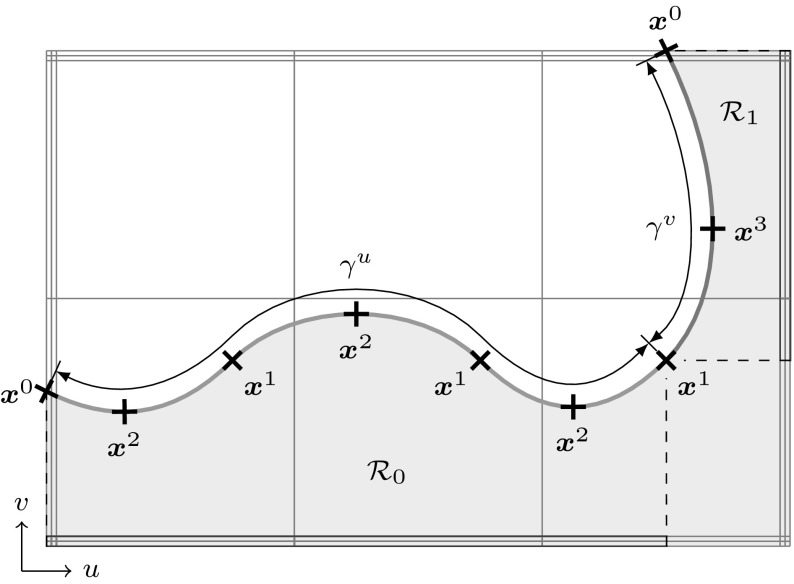
Determination of the trimming curve portions $\gamma ^{u}$ and $\gamma ^{v}$ which are associated to the parametric direction $u$ and $v,$ respectively. The boundary of the related regions ${\mathcal {R}}_{i}$ within the trimmed domain are indicated by *dashed lines*. Characteristic points ${\varvec{x}}$ are marked by *crosses* which correspond to the sloped of the curve. The point type is denoted by the related superscript

They represent characteristic points commonly used in surface-to-surface intersection schemes (i.e., types 0, 2, and 3) [221], along with an additional point previously not utilized (type 1). The main purpose of this classification is to detect portions $\gamma$ of a trimming curve that are associated to either the $u$-direction, i.e., $\gamma ^{u},$ or the $v$-direction, i.e., $\gamma ^{v},$ of the parameter space. With this in mind, the most significant points are those of types 0 and 1, because they indicate a possible transition from $\gamma ^{u}$ to $\gamma ^{v}.$ Each $\gamma$ together with its opposing edge of the parameter space specifies a four-sided regions ${\mathcal {R}}.$ An example of a segmentation of ${\mathcal {A}}^{\text{v}}$ is illustrated in Fig. 43. Note that not every characteristic point of type 1, i.e., ${\varvec{x}}^{1},$ yields a new portion. Hence, the sequence of characteristic points has to be examined rather than the classification of individual points.

Once all reconstruction regions ${\mathcal {R}}$ are detected, the parameterization of adjacent patches is aligned. The following knot cross-seeding procedure establishes a one-to-one relation of points along the intersection curve ${\hat{\varvec{C}}}(s)$ in model space and the related trimming curves of the surfaces ${\varvec{S}}_{i}(u,\,v).$ Firstly, ${\hat{\varvec{C}}}(s)$ is refined so that it is defined by Bézier segments, i.e., the multiplicity of all knots is equal to the curve’s degree. Furthermore, each ${\varvec{S}}_{i}(u,\,v)$ is subjected to knot insertion in the $u$-direction and $v$-direction at their characteristic points ${\varvec{x}}$ of $\gamma ^{u}$ and $\gamma ^{v}$ portions, respectively. Thus, the knot vectors of the surfaces incorporate the locations of all ${\varvec{x}}$. In the next step, the knot information is exchanged across the different objects involved in order to define a knot vector superset. During this process, new Bézier segments are introduced to ${\hat{\varvec{C}}}(s).$ In particular, knots are added at the parametric values $\hat{s}$ that correspond to the knots of ${\varvec{S}}_{i}(u,\,v).$ The values $\hat{s}$ are determined by minimizing the distance of ${\hat{\varvec{C}}}(s)$ to isocurves ${\varvec{C}}^{iso}(u)$ and ${\varvec{C}}^{iso}(v)$ of each ${\varvec{S}}_{i}(u,\,v)$ and the fixed parameters of these isocurves are determined by the knot values of the surfaces. As a result, the refined intersection curve and its superset knot vector reflect the knots and topological characteristics of itself, the related surfaces, and their trimming curves. Finally, this information is passed on to the surfaces, i.e., all ${\varvec{S}}_{i}(u,\,v)$ are refined at the interior knots of ${\hat{\varvec{C}}}(s)$ including the knots of the adjacent ${\varvec{S}}_{j}(u,\,v),\, j\ne i.$ This is done by minimizing the distance between the points of ${\hat{\varvec{C}}}({s})$ and ${\varvec{S}}_{i}(u,\,v)$. The exchange of knot data is necessary in order to guarantee that patches are connected along their intersection after the reconstruction.

Reparameterization is required to obtain a conforming basis for each four-sided region ${\mathcal {R}}.$ Suppose ${\mathcal {R}}$ is related to a $\gamma ^{v}$-portion^19^ of a trimming curve, then it is described by a set of isocurves $\{{\varvec{C}}^{iso}_{k}(u)\}_{k=1}^{K}$ along fixed parameter values $s^{iso}_{k}.$ Note that the values $s^{iso}_{k}$ correspond to the parameterization of the intersection curve ${\hat{\varvec{C}}}(s)$ rather than the *v*-direction of the trimmed surface. Thus, the reparameterized region ${\tilde{\mathcal {R}}}$ will be conformal with ${\hat{\varvec{C}}}(s)$ and the reparameterized counterpart of an adjacent surface will be conformal as well. Due to the cross-seeding process, $\gamma ^{v}$ is linked to at least one Bézier segment of ${\hat{\varvec{C}}}(s).$ The positions $s^{iso}_{k}$ of the isocurves ${\varvec{C}}^{iso}_{k}(u)$ are determined by the endpoints and Greville abscissae of these Bézier segments. The corresponding parametric values in the $u$-direction are labeled $\hat{u}_{k}$ and represent the locations where the distance of ${\varvec{C}}^{iso}_{k}(u)$ is minimal to ${\hat{\varvec{C}}}(s).$ Knot insertion at $\hat{u}_{k}$ is applied to extract the part of ${\varvec{C}}^{iso}_{k}(u)$ that is within the domain of interest, i.e., the current four-sided region ${\mathcal {R}}.$ The values $\hat{u}_{k}$ vary for each ${\varvec{C}}^{iso}_{k}(u)$ since they are distributed close to the trimming curves. In other words, parameter intervals of the isocurves within ${\mathcal {R}}$ do not match in general. To overcome this issue, all ${\varvec{C}}^{iso}_{k}(u)$ are reparameterized to be specified by the *same* basis.

The simplest way to establish the reparameterization is to use a linear coordinate transformation so that all isocurves are defined over a common range, combined with a subsequent accumulation of the shifted interior knots of each knot vector. However, this technique yields a large number of basis function since interior knots of isocurves with different initial intervals do not coincide after the transformation. Furthermore, the size of the resulting knot spans may vary excessively because the alteration of shifted interior knots can be arbitrarily small.

Hence, a nonlinear reparameterization is preferred. A set of functions $\{f_{k}({\tilde{u}})\}_{k=1}^{K}$ is sought that maps the corresponding ${\varvec{C}}^{iso}_{k}(u)\in [a_{k},\,b_{k}]$ to a common range ${\varvec{C}}^{iso}_{k}({\tilde{u}})\in [c,\,d]$ without modifying interior knots. These functions can be represented by univariate splines with scalar coefficients $c^{k}_{i},$ i.e.,

74 $$\begin{aligned} f_{k}({\tilde{u}})&=\sum _{i=0}^{I-1}B_{i,q}({\tilde{u}})c^{k}_{i},\quad q>1,\quad k=1,\ldots ,K. \end{aligned}$$

Note that the linear coordinate transformation is a special case of this formulation where the degree of the function is set to $q=1.$ The composite of an isocurve ${\varvec{C}}^{iso}_{k}(u)$ and its $f_{k}({\tilde{u}})$ determines the reparameterization

75 $$\begin{aligned} {\varvec{C}}^{iso}_{k}({\tilde{u}})={\varvec{C}}^{iso}_{k}(f_{k}({\tilde{u}})),\quad {\tilde{u}}\in [c,\,d]. \end{aligned}$$

The degree of the resulting curve is given by ${\tilde{p}}=pq$ where $p$ refers to the original degree of the isocurve. If the longest isocurve is taken as target parameterization, it can be adjusted by using conventional degree elevation to ${\tilde{p}}.$ The other curves are subjected to a nonlinear reparameterization based on their $f_{k}({\tilde{u}}).$ For technical details on nonlinear reparameterization of curves the interested reader is referred to the textbook [230].

It remains to find a way to coordinate the individual $f_{k}({\tilde{u}})$ to obtain a *global* reparameterization for the whole reconstruction domain ${\mathcal {R}}$ that yields a new valid tensor product parameter space ${\tilde{\mathcal {R}}}.$ The key idea is to represent the global transformation as a spline surface $f({\tilde{u}},\,s).$ This surface includes all $f_{k}({\tilde{u}})$ as isocurves, i.e., $f({\tilde{u}},\,s^{iso}_{k})=f_{k}({\tilde{u}}).$ The bivariate reparameterization is given by

76 $$\begin{aligned} f({\tilde{u}},\,s) = \sum _{i=0}^{I-1} \sum _{j=0}^{J-1} B_{i,{\tilde{p}}} ({\tilde{u}}) B_{j,p_{s}}(s) c_{i,j}, \end{aligned}$$

with the degree $p_{s}$ of the intersection curve segment and a grid of scalar control coefficients $c_{i,j}.$ If the degree in the $v$-direction of the trimmed surface varies from $p_{s},$ the degree of the segment may be adjusted by means of degree elevation. Equation (76) can be represented as a non-parametric surface by linking the coefficients $c_{i,j}$ to their Greville abscissae (22).

**Fig. 44:**
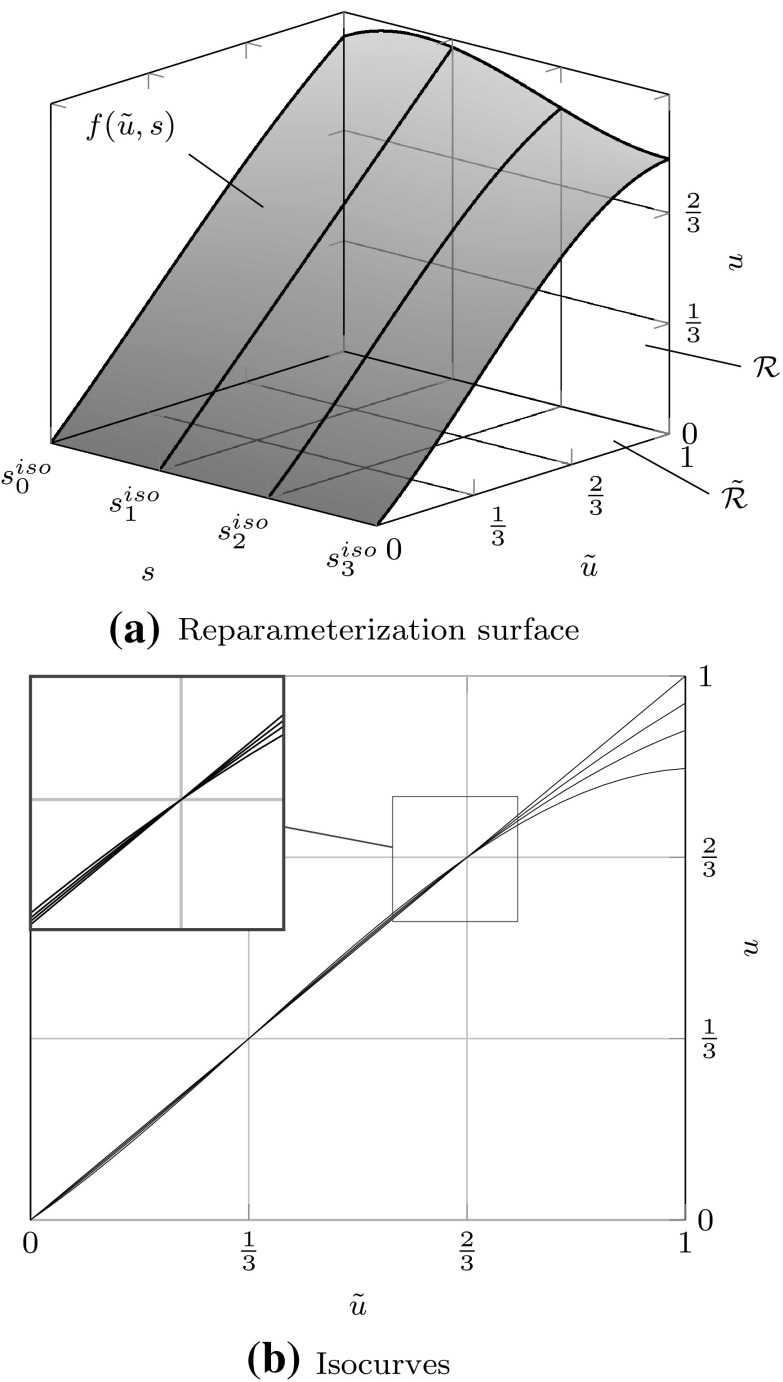
Reparameterization of a trimmed parameter space of a bicubic Bézier patch: **a** surface $f({\tilde{u}},\,s)$ that reparameterizes the trimmed parameter space $u$ to a regular one ${\tilde{u}}$ indicated by the vertical and plane grid, respectively. *Lines* on the surface mark the associated isocurve along $s^{iso}_{k}.$ **b** Profile of the isocurves $f_{k}({\tilde{u}})$

The corresponding control points are defined as

77 $${c_{i,j}} = \left[ {\begin{array}{{c}} {({{\tilde {u}}_{i + 1}} + {{\tilde {u}}_{i + 2}} + \cdots + {{\tilde {u}}_{i + \tilde {p}}})/\tilde {p}}\\ {({s_{j + 1}} + {s_{j + 2}} + \cdots + {s_{j + {p_{s}}}})/{p_{s}}}\\ {{c_{i,j}}} \end{array}} \right].$$

The coefficients $c_{i,j}$ can be defined by the user as long as the following restrictions are met:

(i) The function $f({\tilde{u}},\,s)$ must be *strictly monotonic* in the ${\tilde{u}}$-direction so that intervals do not overlap.
(ii) The spline surface must employ the same target knot vector ${\tilde{\varXi}}$ in the ${\tilde{u}}$-direction.
(iii) Each knot value $u_{i}$ of the initial knot vectors must be mapped to a distinct ${\tilde{u}}_{i}\in {\tilde{\varXi} }$ for all isocurves, i.e., $u_{i}=f({\tilde{u}}_{i},\,s^{iso}_{k}),\, k=1,\ldots ,K.$

An illustration of such a bivariate reparameterization function $f({\tilde{u}},\,s)$ is provided in Fig. 44a and the corresponding isocurves are shown in Fig. 44b. It should be noted that the parameter space spanned by the ${\tilde{u}}$-axis and $s$-axis is defined by straight parameter lines only, in contrast to the original basis spanned by the $u$-axis and $s$-axis. It is emphasized that the graphs in Fig. 44b intersect at the common interior knots ${\tilde{u}}_{i}=u_{i}=\left\{ \frac{1}{3},\,\frac{2}{3}\right\} .$

The final step of the reconstruction scheme is to determine the control points of the reparameterized regions ${\tilde{\mathcal {R}}}.$ Therefore, we recall the specification of the control points ${\tilde{\varvec{c}}}_{i}^{k}$ of isocurves ${\varvec{C}}^{iso}_{k}(u)$ as a weighted combination of surface control points ${\varvec{c}}_{i,j}$

78 $$\begin{aligned} {\tilde{\varvec{c}}}_{i}^{k}&=\sum _{j=0}^{J-1} B_{j,q}(s^{iso}_{k}) {\varvec{c}}_{i,j},&k=1,\ldots ,K. \end{aligned}$$

Isocurves have been introduced at the Greville abscissae of the Bézier segments along the reconstruction boundary $\gamma ^{v}.$ Hence, the number of isocurves is equal to the number of unknowns, i.e., $J=K,$ and the control points ${\varvec{c}}_{i,j}$ can be computed based on the known isocurve control points ${\tilde{\varvec{c}}}_{i}^{k}$ by inverting the system of equations (78). The control points along the boundary $\gamma ^{v}$ are already known beforehand, i.e., the control points of ${\hat{\varvec{C}}}(s),$ and do not need to be computed explicitly.

**Fig. 45:**
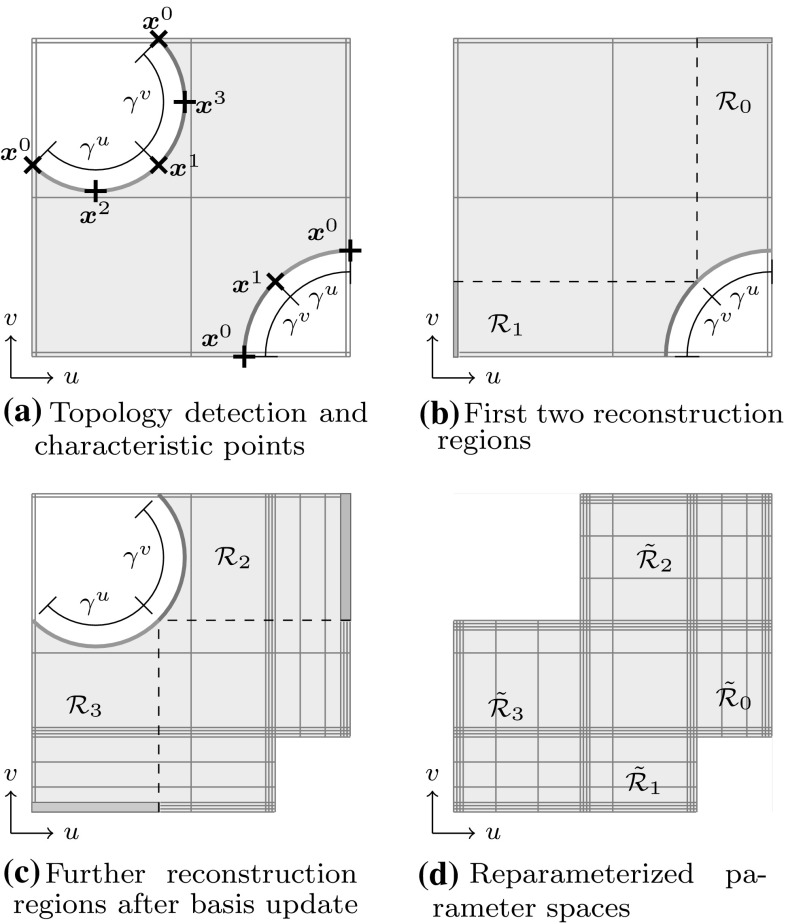
Successive evolution of the isocurve reconstruction of a quadratic trimmed patch using a quadratic reconstruction function

It is quite astonishing that the procedure described remains the same when multiple trimming curves are involved. Instead of assessing the topology of all trimming curves at once, the trimming curve or more precisely each $\gamma$ is processed successively and independently of each other. In fact, it does not matter if the portions $\gamma$ originate from one or several trimming curves. After each reparameterization the parameter space is updated and the next region is addressed. The iterative evolution of the reconstructed regions ${\tilde{\mathcal {R}}}$ is displayed in Fig. 45. To be clear, the regions ${\mathcal {R}}_{0}$ and ${\mathcal {R}}_{1}$ shown in Fig. 45b and the regions ${\mathcal {R}}_{2}$ and ${\mathcal {R}}_{3}$ displayed in Fig. 45c are not constructed at the same time.

The final outcome of the reconstruction is a new set of patches with aligned parameter spaces that share the control point of their intersection curves. Thus, the reconstructed object is watertight. It is emphasized that this holds true even for non-manifolds. These benefits come at the price of an alteration of the initial geometry and an increase of the degrees of freedom. Since the concept has been presented just recently, there are several open research topics to explore. For instance, an estimation of the geometrical error introduced with respect to the degree of the reparameterization function and the number of isocurves would be of great interest. This could be the basis for an optimization procedure for the definition of the reparameterization function. Another topic might be the quality of the resulting elements in model space, especially at the transitions from $\gamma ^{u}$ to $\gamma ^{v}$ regions.

We close the discussion of the isocurve reconstruction approach with some application remarks. Firstly, the concept can also be applied locally. In this paper it is focused on the tensor product case where refinement propagates through the whole domain for the sake of simplicity. A locally reconstructed parameter space may be represented by any local basis like T-splines, hierarchical B-splines, or LR-splines. Secondly, the degree of the resulting patches may become large, depending on the degree of the reparameterization function. It might be beneficial to apply a degree reduction technique after the reconstruction, but this introduces additional approximation errors. Finally, the intersection curves should have a good parameterization since they play an essential role during the reconstruction. Therefore, it might be advisable to reparameterize the intersection curve, e.g., by its chord length, at the beginning of the overall procedure.

#### Reconstruction by Triangular Bézier Splines

Another recent attempt has been proposed by Xia and Qian [314]. They employ triangular Bézier patches (36) to convert trimmed models to watertight representations. The convergence behavior of these splines has been assessed by these authors and co-workers in [315]. The conversion involves the following steps: (i) subdivision of all surfaces into tensor product Bézier patches, (ii) exact representation of non-trimmed patches by two Bézier triangles, (iii) knot cross-seeding between adjacent patches, (iv) approximation of the region along the trimming curve using Bézier triangles, and (v) substitution of the resulting control points of the approximate trimming curve by corresponding control points of the intersection curve in model space.

The first step can be easily accomplished by means of knot insertion. The second one is performed following Goldman and Filip [100]. In particular, a non-trimmed tensor product patch with control points ${\varvec{c}}^{\square }_{m,n}$ can be converted to two triangular Bézier patches by

79 $$\begin{aligned} {\varvec{c}}^{\triangle }_{i,j,k}&= \frac{1}{\left( {\begin{array}{c}p+q\\ q\end{array}}\right) } \sum _{m=0}^{i} \sum _{n=\max \left\{ 0,j-p+m\right\} }^{\min \left\{ j,q-i+m\right\} } {\varvec{c}}^{\square }_{m,n} \left( {\begin{array}{c}i\\ m\end{array}}\right) \left( {\begin{array}{c}j\\ n\end{array}}\right) \nonumber \\&\quad \times \left( {\begin{array}{c}p+q-i-j\\ p+n-m-j\end{array}}\right) , \end{aligned}$$

where $i+j+k= p+ q$ and $$\left( {\begin{array}{c}\alpha \\ \beta \end{array}}\right)$$ are binomial coefficients defined as

80 $$\begin{aligned} \left( {\begin{array}{c}\alpha \\ \beta \end{array}}\right) := \frac{\alpha !}{(\alpha -\beta )!\beta !}. \end{aligned}$$

Equation (79) yields the control points ${\varvec{c}}^{\triangle }_{i,j,k}$ of one triangular patch using ${\varvec{c}}^{\square }_{m,n}{\text {:}}\,0\leqslant m\leqslant p; \,0\leqslant n\leqslant q.$ The control points of the other triangular patch are obtained by reversing the order of the original control points, i.e., ${\varvec{c}}^{\square }_{p-m,q-n}{\text {:}}\,0\leqslant m\leqslant p;\, 0\leqslant n\leqslant q.$ The degree of the resulting patches is determined by $p+ q.$ It is emphasized that this transformation does not introduce an approximation error.

**Fig. 46:**
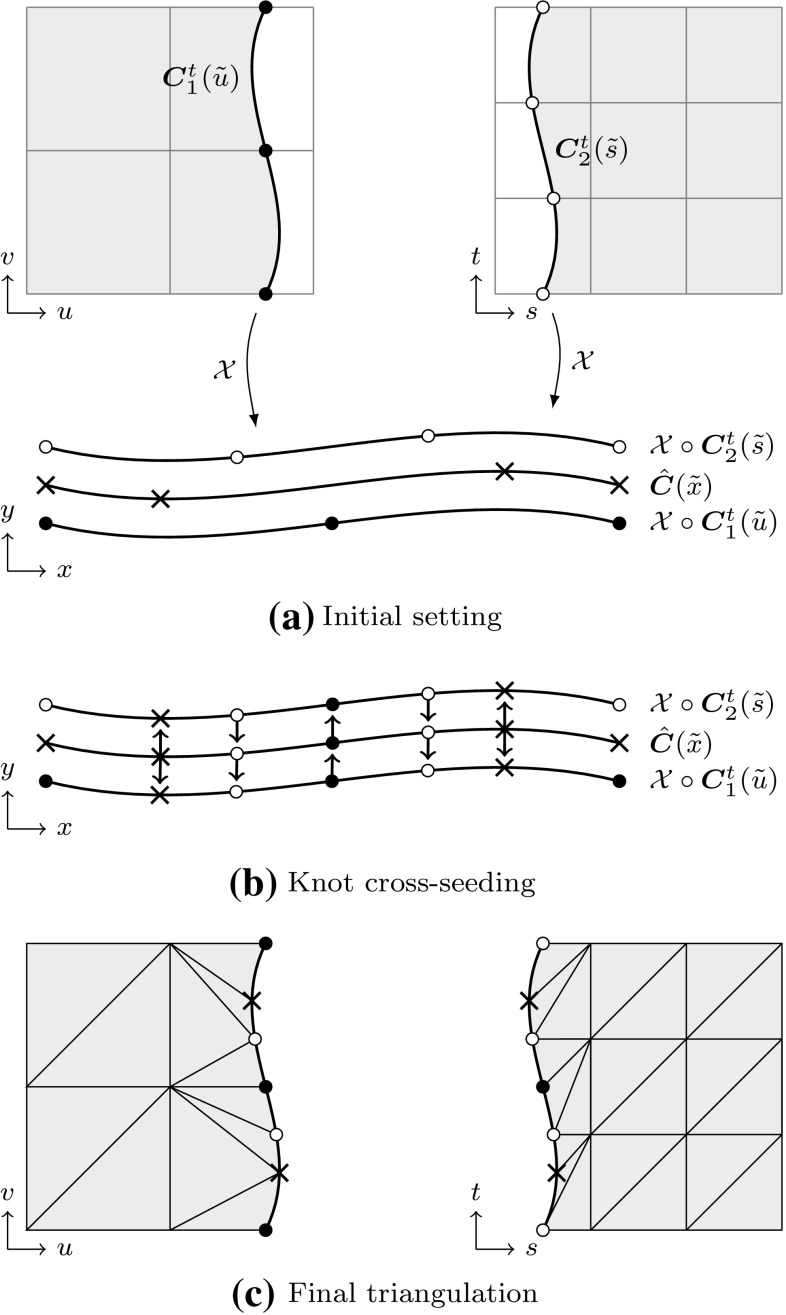
Generation of conforming triangulations along the intersection of two patches: **a** definition of Bézier segments of the intersection curve ${\hat{\varvec{C}}}({\tilde{x}})$ and the trimming curves ${\varvec{C}}^{t}_{1}({\tilde{u}})$ and ${\varvec{C}}^{t}_{2}({\tilde{s}}).$ **b** Closest point projection to find corresponding points on the other curves. **c** Addition of Bézier segments due to the exchanged points and specification of associated triangular regions. Segments are marked by *crosses*, *black points*, and *white points* based on their origin. The offset between ${\hat{\varvec{C}}}({\tilde{x}})$ and the images ${\mathcal {X}}\circ {\varvec{C}}^{t}_{1}({\tilde{u}})$ and ${\mathcal {X}}\circ {\varvec{C}}^{t}_{2}({\tilde{s}})$ shall emphasize that they do not coincide in model space. In **b**, *arrows* indicate the projections performed

Next, the relationship of adjacent patches along the intersection is established. This is done similar to the knot cross-seeding procedure described in the previous subsection. Hence, we adopt this term here as well. Figure 46 summarizes the basic procedure. Firstly, the intersection curve ${\hat{\varvec{C}}}({\tilde{x}})$ in model space and the trimming curves ${\varvec{C}}^{t}_{1}({\tilde{u}})$ and ${\varvec{C}}^{t}_{2}({\tilde{s}})$ are subdivided into Bézier segments at their knot values and intersections with the trimmed parameter space. Then, the endpoints of these segments are projected to the other curves and the corresponding parametric values are computed. In other words, the trimming curve are refined based on the knot information of the other trimming curve and the intersection curve in model space. Consequently, the resulting Bézier segments of a curve have corresponding counterparts in the other curves. However, the distinct segments do not coincide in model space. At this point, the purpose of the knot cross-seeding is to obtain an aligned triangulation along the intersection. Triangular patches are specified within each trimmed surface so that one of their boundaries represents a Bézier segment.

The construction of these triangular Bézier patches which are arbitrarily located within the trimmed surface is performed accordingly to Lasser [182]. In general, a Bézier triangle *T* of degree ${\tilde{p}}$ in a tensor product basis of a surface ${\varvec{R}}(u,\,v)$ of degrees $p$ and $q$ yields a triangular patch ${\varvec{S}}^{\triangle }({r,\,s,\,t})$ of degree ${\tilde{p}} (p+q).$ Xia and Qian [314] focus on the linear case, i.e., ${\tilde{p}}=1,$ meaning that the trimming curve is approximated by linear segments. Thus, the composition ${\varvec{S}}^{\triangle }({r,\,s,\,t})={\varvec{R}}(T(r,\,s,\,t))$ is given by

81 $$\begin{aligned} {\varvec{S}}^{\triangle }({r,\,s,\,t})=\sum _{|{\mathbf {I}}|=p+q} B_{{\mathbf {I}},p+q}({r,\,s,\,t}) {\varvec{c}}^{\triangle }_{{\mathbf {I}}}, \end{aligned}$$

where $B_{{\mathbf {I}},p+q}$ refer to the Bernstein basis (37) of the triangular patch, ${\mathbf {I}}$ is an index triplet $(i,\,j,\,k),$ and $|{\mathbf {I}}|=i+j+k.$ It remains to determine the corresponding control points ${\varvec{c}}^{\triangle }_{{\mathbf {I}}}.$ Therefore, the construction points ${{\varvec{R}}}^{p,q}_{0,0}(u^{p}_{{\mathbf {I}}^u},\,v^{q}_{{\mathbf {I}}^v})$ of the blossom of ${\varvec{R}}(u,\,v)$ are needed. Regarding the $u$-direction, for instance, these points are recursively defined by

82 $$\begin{aligned} {\varvec{R}}^{a,b}_{i,j}(u^{a}_{{\mathbf {I}}^{u}},\,v^{b}_{{\mathbf {I}}^{v}})&= \left( 1-u_{{\mathbf {I}}^u_{a}} \right) {\varvec{R}}^{a-1,b}_{i,j}(u^{a-1}_{{\mathbf {I}}^{u}},\,v^{b}_{{\mathbf {I}}^{v}}) \nonumber \\&\quad + u_{{\mathbf {I}}^{u_a}} {\varvec{R}}^{a-1,b}_{i+1,j}(u^{a-1}_{{\mathbf {I}}^{u}},\,v^{b}_{{\mathbf {I}}^{v}}), \end{aligned}$$

where the control points of the surface ${\varvec{R}}(u,\,v)$ are used as initial values ${\varvec{R}}^{0,0}_{i,j}.$ The construction in the $v$-direction is performed in an analogous manner. The superscripts *a* and *b* denote distinct steps of the recurrence relation in the $u$-direction and $v$-direction, respectively. Note that Eq. (82) is an adaptation of the de Casteljau algorithm that allows employing new parameter values $u_{{\mathbf {I}}^{u}_{a}}$ in every iteration. Likewise, the index tuples are given by ${\mathbf {I}}^{v}={\mathbf {I}}^{v}_{1}+\cdots +{\mathbf {I}}^{v}_{b}$ and ${\mathbf {I}}^{u}={\mathbf {I}}^{u}_{1}+\cdots +{\mathbf {I}}^{u}_{\alpha }$ with $\alpha$ referring to the related superscript, i.e., *a* or $a-1.$ Using this recursion, the control points of the triangular patch ${\varvec{S}}^{\triangle }({r,\,s,\,t})$ are obtained by

83 $$\begin{aligned} {\varvec{c}}^{\triangle }_{{\mathbf {I}}} = \sum _{{\mathbf {I}}^{u}+{\mathbf {I}}^{v}={\mathbf {I}}} \frac{1}{\left( {\begin{array}{c}p+q\\ {\mathbf {I}}\end{array}}\right) } {\varvec{R}}^{p,q}_{0,0}(u^{p}_{{\mathbf {I}}^{u}},\,v^{q}_{{\mathbf {I}}^{v}}), \end{aligned}$$

with

84 $$\begin{aligned} {\mathbf {I}}^{u}={\mathbf {I}}^{u}_{1}+\cdots +{\mathbf {I}}^{u}_{q}&&\,\text {and}\,&&{\mathbf {I}}^{v}={\mathbf {I}}^{v}_{1}+\cdots +{\mathbf {I}}^{v}_{p}, \end{aligned}$$

where each of these index triples consists of

85 $$\begin{aligned} {\mathbf {I}}^{\alpha }_{\beta }&=\left( i^{\alpha }_{\beta },\,j^{\alpha }_{\beta },\,k^{\alpha }_{\beta }\right) \quad \,\text {with}\,\quad i^{\alpha }_{\beta },\,j^{\alpha }_{\beta },\,k^{\alpha }_{\beta }\in \{0,\,1\}, \end{aligned}$$

and further

86 $$\begin{aligned}&\left| {\mathbf {I}}^{u}\right| =\left| {\mathbf {I}}^{u}_{1}\right| +\cdots +\left| {\mathbf {I}}^{u}_{p}\right| =p, \end{aligned}$$

87 $$\begin{aligned}&\left| {\mathbf {I}}^{v}\right| =\left| {\mathbf {I}}^{v}_{1}\right| +\cdots +\left| {\mathbf {I}}^{v}_{q}\right| =q, \end{aligned}$$

88 $$\begin{aligned}&|{\mathbf {I}}|=\left| {\mathbf {I}}^{u}\right| +\left| {\mathbf {I}}^{v}\right| =p+q. \end{aligned}$$

This procedure is applied to cover the valid area of every trimmed Bézier surface by a set of triangular Bézier patches. Each Bézier segment of a trimming curve is represented by an edge of such a triangular patch. Finally, those edges are replaced by the corresponding Bézier segment of the intersection curve in model space. Since this substitution is carried out for all patches, a seamless join between adjacent surfaces is obtained. The approximation error introduced may be controlled by refining the patches along the trimming curves.

It is worth noting that Xia and Qian [314] use their reconstruction procedure as an intermediate step in order to set up a volumetric parameterization of B-Rep models. The watertight triangular Bézier surface representation provides the starting point for a construction of volumetric Bézier tetrahedra.

### Local Approaches

Local techniques employ a completely different philosophy than their global counterparts, that is, the geometry model is not modified but the analysis has to deal with all deficiencies of trimmed solid models. Thereby, the trimmed parameter space is used as background parameterization for the simulation while the trimming curves determine the domain of interest. Hence, the analyzed area is embedded in a regular grid of knot spans which consists of *interior*, *exterior*, and *cut* elements. The following subsections discuss (i) the detection of theses distinct element sets, (ii) the integration of cut elements, (iii) the treatment of multipatch geometries, and (iv) the stability of a trimmed basis.

#### Element Detection

**Fig. 47:**
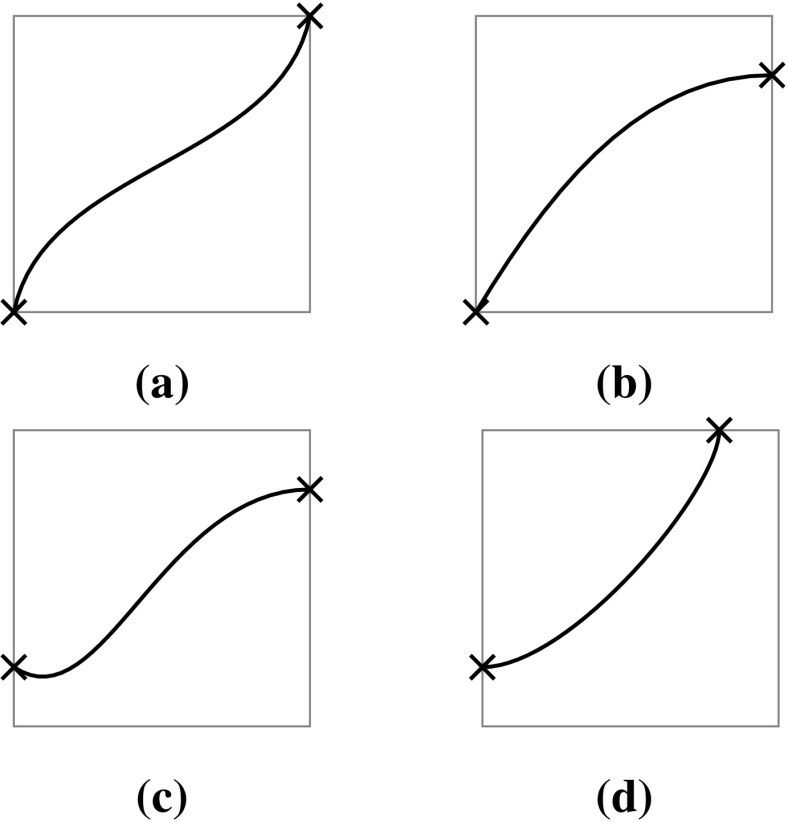
Illustration of the most common valid cutting patterns of a single knot span. The actual element type is determined by the direction of the trimming curve. The *crosses* highlight the intersection points of the trimming curve with the element

Before the actual analysis can be performed, the various element types and their position within the trimmed basis need to be identified. Interior elements are defined by non-zero knot spans that are completely within the valid domain and can be treated as in regular isogeometric analysis. Exterior ones, on the other hand, can be ignored since their entire support is outside of the domain of interest. Cut elements require special attention. One of the advantages of local approaches is that the cutting patterns of these elements are relatively simple compared to the complexity of the overall trimming curve. Figure 47 depicts topological cases of cut elements that are usually considered, e.g., [160, 161, 199, 202, 260]. It should be pointed out that other cases may exits as well, e.g., an element containing more than one trimming curve. These situations occur especially when the basis is very coarse. In general, the complexity of a trimming curve’s topology within an element decreases as the fineness of the parameter space increases. Hence, (local) refinement is a common way to resolve invalid cutting patterns. This refinement may be performed for integration purpose only. Thus, no new knots are introduced, but the invalid element is subdivided in several valid integration regions. An alternative is to extend the valid cutting patterns as suggested by Wang et al. [307] or the construction of tailored integration rules for each cut element as proposed by Nagy and Benson [211]. However, the benefit of a restricted number of trimming cases facilitates the subsequent integration process.

**Fig. 48:**
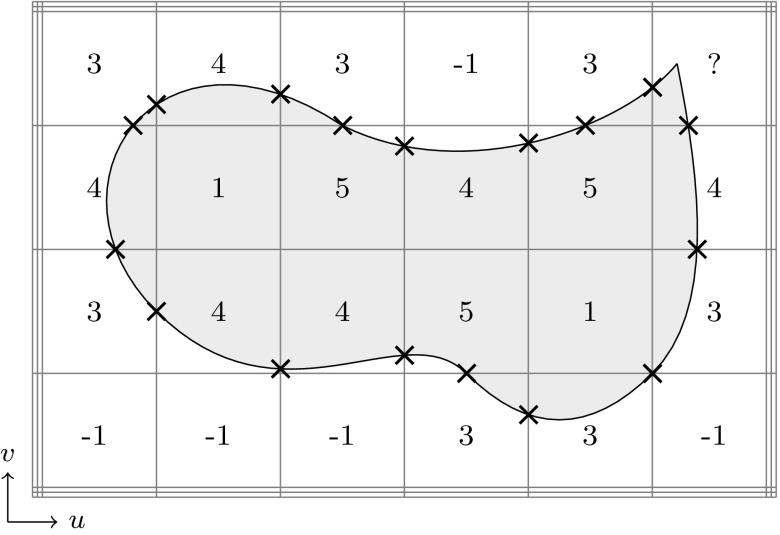
Trimmed parameter space and corresponding element types: *1* labels untrimmed knot spans whereas −*1* denotes knot spans which are outside of the computational domain. In case of trimmed knot spans the element type indicates the number of interior edges, i.e., *3*, *4*, or *5*. The *question mark* indicates a special case. The intersections of the trimming curve with the parameter lines are highlighted by *crosses*

Considering the situations shown in Fig. 47, cut elements have either 3, 4, or 5 edges, where one of them is a portion of the trimming curve. In this paper, we adapt the notation of Schmidt et al. [260] and label the type of cut elements by their number of edges. Interior and exterior elements are referred to as elements of type 1 and −1, respectively. Figure 48 illustrates a trimmed parameter space and the related element types. Note that the knot span in the upper right corner is an example of an invalid case since smooth element edges are usually assumed for the numerical integration. Possible strategies to deal with this element include subdivision into several integration regions, treatment as a type 4 element with two curved edges, or knot refinement through the kink of the curve. In general, kinks and straight trimming curves that are aligned with parameter lines are usual suspects for introducing special cases.

The portions of the trimming curve which are within each element have to be determined in order to get a proper description of cut knot spans. In particular, the intersections of the parameter grid with the trimming curve ${\varvec{C}}^{t}({\tilde{u}})$ are required, together with the corresponding parametric values ${\tilde{u}}^{+}.$ The overall element detection task consists of the classification of knot span with respect to the trimming curves and the determination of trimming curve portions related to cut elements.

Kim et al. [160, 161] and Schmidt et al. [260] presented two different algorithmic solutions for the element detection problem. The procedure suggested by the former can be summarized by:

(i) Compute the minimal signed distance $d_{i,j}$ of the center of each non-zero knot span to all trimming curves to separate *interior* and *exterior* elements.
(ii) Identify *cut* elements by comparing $\left|d_{i,j} \right|$ with the radii $r^{in}_{i,j}$ and $r^{out}_{i,j}$ of the inscribed and circumscribed circles of the element. If $r^{in}_{i,j}\leqslant \left|d_{i,j} \right|<r^{out}_{i,j},$ the signed distance of the element corner nodes to the trimming curve are computed and compared as well.
(iii) Compute *intersection points* for each element cut by the trimming curve.

**Fig. 49:**
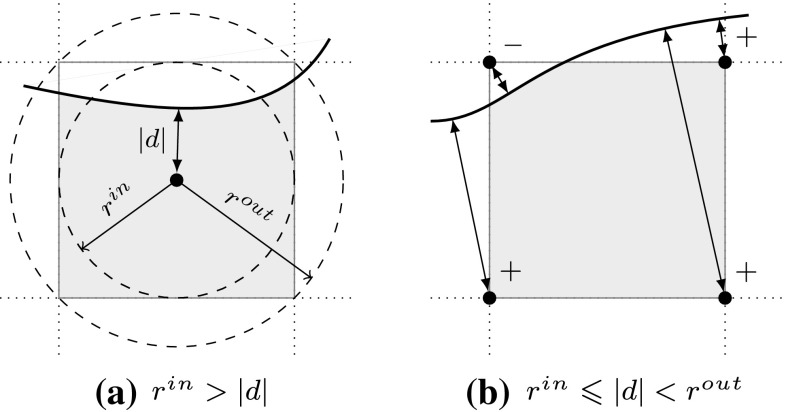
Detection of cut elements according to Kim et al. [160, 161]: **a** first assessment based on the inscribed and circumscribed *circles* of the element and if necessary, **b** further comparison of the signed distance of the element corners

Both cases of the second step which specify cut elements are illustrated in Fig. 49. In Fig. 49a, the distance of the element’s center to the trimming curve is smaller than the radius of the inscribed circle, whereas in Fig. 49b, the cut element is identified since the signed distances of its corner nodes are positive and negative.

On the other hand, Schmidt et al. [260] recommend to label all non-zero knot spans as interior elements as starting point for the following procedure:

(i) Determine all *intersection points* of the trimming curve and the grid produced by the tensor product of the knot vectors and sort them in a nondecreasing order with respect to the related values ${\tilde{u}}^{+}.$
(ii) Assign the element type of *cut* elements based on the position of successive intersection points.
(iii) Detect *exterior* elements based on their position relative to the cut elements.

**Fig. 50:**
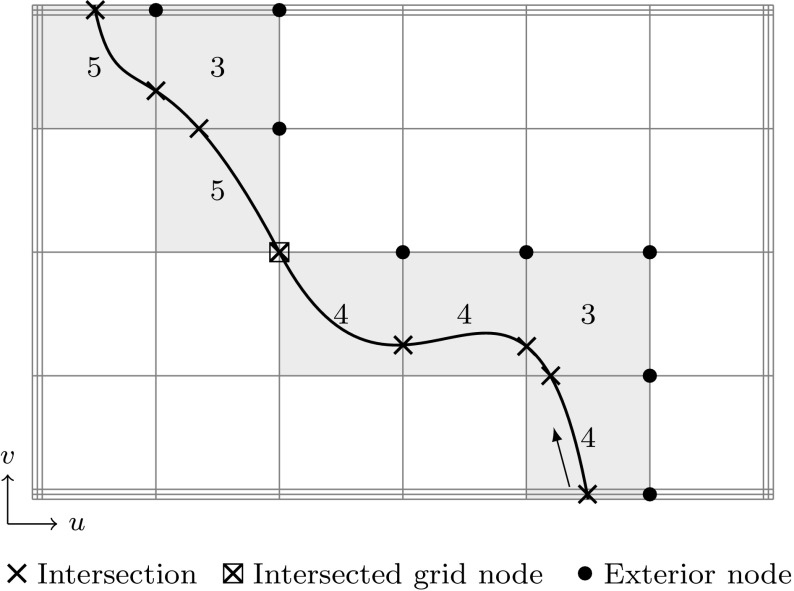
Starting point for the separation of interior and exterior elements following the procedure of Schmidt et al. [260]. White knot spans are not classified yet. The *arrow* indicates the direction of the trimming curve

It should be noted that successive intersection points mark start and end of trimming curve portions within an element. For the last task, the exterior nodes of cut elements can be used to initialize an incremental algorithm setting adjacent elements which are not labeled as cut elements to −1 [286]. Figure 50 illustrates the situation described. The nodes of these exterior elements are then used to determine further exterior elements. The procedure stops as soon as there are any adjacent elements of type 1 left.

On this basis, it may be concluded that the present approaches tackle the problem from two different directions. The former puts the element type in the focus with a subsequent calculation of the intersections of cut elements with the trimming curve, whereas the latter computes all intersections and derives the type of the elements afterwards. In general, the most important property of an element detection algorithm is its robustness since it hardly effects the overall efficiency of the simulation. Both approaches require a robust implementation of the curve-to-grid intersection computation. The Bézier clipping technique described in Sect. 3.5.2 could be used. The treatment of invalid cutting patterns applies also to both algorithms and depends on the subsequent integration procedure. The main difference between the approaches is that the former relies on a robust implementation of a point projection algorithm in order to determine the signed distance of a point to the trimming curve, while the latter requires a robust technique for detecting exterior elements based on the intersection information. There is perhaps no objective way to prefer one scheme to the other, but we would like to share our experiences with both schemes by mentioning some possible pitfalls in the following paragraphs.

The first point addresses the detection of exterior elements based on their relative position to cut elements. The starting point is illustrated in Fig. 50. Elements of type 1 are changed to type −1 if they are adjacent to the exterior nodes of cut and exterior elements. The search for adjacent elements can be performed in an incremental manner as it is done in “flood fill” algorithms, which are commonly used in graphics software [286]. It should, however, be emphasized that intersected grid nodes need special attention since the search for adjacent elements might propagate at these points to the valid domain. Furthermore, a proper treatment of zero knot spans is required.

The other note is concerned with the calculation of the signed distance to trimming curves. The shortest distance $d_{t}$ of a test point ${\varvec{x}}_{t}$ to a trimming curve ${\varvec{C}}^{t}({\tilde{u}})$ is defined as

89 $$\begin{aligned} d_{t} = \min \left\{ \left\Vert{\varvec{C}}^{t}({\tilde{u}})-{\varvec{x}}_{t} \right\Vert\right\} = \left\Vert{\varvec{C}}^{t}({\tilde{u}}^{*})-{\varvec{x}}_{t} \right\Vert. \end{aligned}$$

A Newton–Raphson iteration scheme is employed to determine the parametric values ${\tilde{u}}^{*}$ [192, 230]. The corresponding sign *s* indicates on which side of ${\varvec{C}}^{t}({\tilde{u}})$ the point ${\varvec{x}}_{t}$ is located. It can be computed by the cross product of the tangent vector ${\varvec{t}}=(t_{u},\,t_{v})^{\intercal }$ at the projected point ${\varvec{x}}_{p}={\varvec{C}}^{t}({\tilde{u}}^{*})$ and the direction vector ${\varvec{d}}=(d_{u},\,d_{v})^{\intercal }$ from ${\varvec{x}}_{p}$ to ${\varvec{x}}_{t}.$ In two dimensions, the analog to the cross product is given by the determinant, hence the sign is calculated by

90 $$\begin{aligned} s=t_{u} d_{v} - t_{v} d_{u}. \end{aligned}$$

**Fig. 51:**
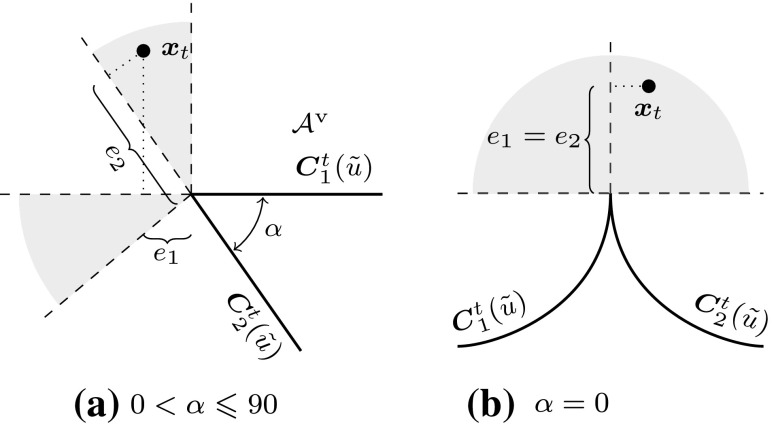
Determination of the correct sign in case of multiple trimming curves ${\varvec{C}}^{t}_{i}({\tilde{u}})$ which describe an acute angle. The area which returns ambiguous signs is indicated in *gray*. (Courtesy of Jakob W. Steidl)

In case of *non-smooth* trimming curves, more than one minimum might exist as pointed out in [287]. From a practical point of view this is only relevant if these minima have different signs. Such cases appear in the vicinity of sharp corners as shown in Fig. 51. The correct sign can be determined by the projected distance calculated by the dot product

91 $$\begin{aligned} e_{i}&= {\varvec{v}} \cdot {\varvec{t}}_{i},&i=1,\,2, \end{aligned}$$

92 $$\begin{aligned} s&= \text {sign}\left\{ \min \left\{ e_{1},\,e_{2}\right\} \right\} . \end{aligned}$$

If the angle $\alpha =0,$ i.e., $e_{1}=e_{2},$ the curvatures of the curves may be compared

93 $$\begin{aligned} s= \left\{ \begin{array}{rl} 1 &{} if \kappa _{1}> \kappa _{2},\\ -1 &{} otherwise , \end{array} \right. \end{aligned}$$

where $\kappa _{1}$ denotes the curvature of the curve that ends at the corner.

#### Integration

Various strategies to integrate cut elements ${\tilde{\tau} }\in {\mathcal {A}}^{\text{v}}$ are outlined in this subsection. In general, numerical integration is performed using conventional Gauss–Legendre quadrature. The integral over each ${\tilde{\tau} }$

94 $$\begin{aligned} I_{\tilde{\tau }}&= \int _{\Omega _{\tilde{\tau }}} f({\varvec{x}}) \;\text {d}\Omega _{\tilde{\tau }} \end{aligned}$$

is substituted by a weighted sum of point evaluations

95 $$\begin{aligned} I_{\tilde{\tau }}&\approx \sum _{g=1}^{n} f\left( {\varvec{y}}_{g}\right) G(u_{g},\,v_{g}) J_{{\grave{\tau }}}(\xi _{g},\,\eta _{g}) w_{g}. \end{aligned}$$

**Fig. 52:**
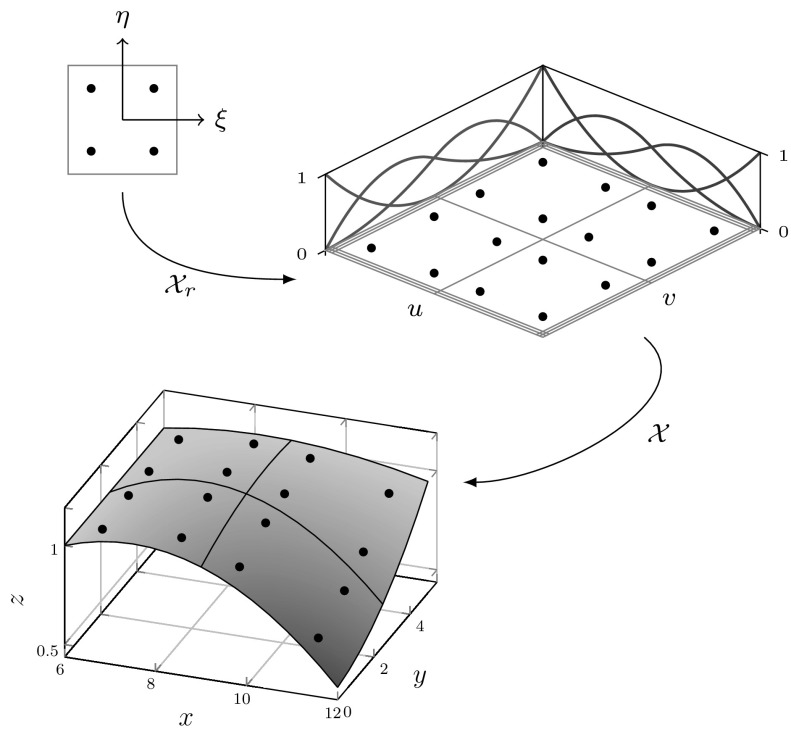
Distribution of quadrature points indicated by *black* points over a regular patch

The related quadrature points ${\varvec{y}}$ and the corresponding weights $w$ are specified in the reference element $\grave{\tau }=[-1,\,1]^{2}.$ The coordinates for the pointwise evaluation of the integrand *f* are determined by the integral transformation ${\mathcal {X}}_{r}(\xi ,\,\eta ){\text {:}}\, {\mathbb {R}}^{2}\mapsto {\mathbb {R}}^{2}$ from $\grave{\tau }$ to ${\tilde{\tau} }$ and the geometrical mapping ${\mathcal {X}}(u,\,v){\text {:}}\,{\mathbb {R}}^{2}\mapsto {\mathbb {R}}^{3},$ i.e., ${\varvec{y}}_{g}={\mathcal {X}}(u_{g},\,v_{g})={\mathcal {X}}({\mathcal {X}}_{r}(\xi _{g},\,\eta _{g}))$ as illustrated in Fig. 52. In order to take these mappings into account, the quadrature weights $w_{g}$ are multiplied by the Gram’s determinant

96 $$\begin{aligned} G(u,\,v)&:=\sqrt{\det \left( {\mathbf {J}}_{{\mathcal {X}}}^{\intercal }(u,\,v) {\mathbf {J}}_{{\mathcal {X}}} (u,\,v)\right) }, \end{aligned}$$

given by the Jacobian matrix ${\mathbf {J}}_{\mathcal {X}}$ of the geometrical mapping. The Jacobian determinant of ${\mathcal {X}}_{r}$

97 $$\begin{aligned} J_{{\grave{\tau }}}(\xi _{g},\,\eta _{g}) = \det \left( {\mathbf {J}}(\xi _{g},\,\eta _{g}) \right) , \end{aligned}$$

is evaluated with respect to the reference coordinates $\xi _{g}$ and $\eta _{g}$ of the integration point ${\varvec{y}}_{g}.$ The definition of the integral transformation ${\mathcal {X}}_{r}$ and the related $J_{{\grave{\tau }}}$ is straightforward in case of regular elements. However, the domain of cut elements is more complex and thus, the definition of ${\mathcal {X}}_{r}$ is more involved.

Numerical integration of cut elements is required in various simulation schemes. Besides the analysis of trimmed geometries, it is also needed in the context of fictitious domain methods and the extended finite element method. There are numerous approaches and a vast body of literature proposing strategies to specify a proper integration of ${\tilde{\tau} }.$ It may be performed so that the trimming curve is taken into account in an exact or approximate manner. In this paper, the main focus is on techniques presented in the context of trimmed NURBS objects. They can be broadly classified into the following categories: (i) local reconstruction, (ii) approximate treatment, and (iii) exact treatment. The former is performed in model space, while the others operate in the parameter space in general.

##### Local Reconstruction

Schmidt et al. [260] suggested to perform the adjustment of the integration region by a local reconstruction of the trimmed patch. Therefore, each cut element in the model space $\tau$ is remodeled as a single reconstruction patch $\hat{\tau }.$ In particular, $\hat{\tau }$ is specified as a Bézier patch with degrees $\hat{p}\geqslant p$ and $\hat{q}\geqslant q,$ where $p$ and $q$ refer to the degrees of the origin surface. The key idea is to represent $\hat{\tau }$ in terms of the original control points of $\tau .$ A transformation matrix ${\mathbf {T}}$ provides the relationship between the control points of the original and reconstructed patch. It can be computed by means of a least squares approximation where the system of equations is given by

98 $$\begin{aligned} {\hat{\mathbf {B}}} {\hat{\mathbf {P}}} = {\mathbf {S}} = {\mathbf {B}} {\mathbf {P}}. \end{aligned}$$

This equation consists of

99 $${\hat{ \mathbf{P}} } = \left[ {\begin{array}{{c}} {{{\hat {\varvec{c}}}_0}}\\ \vdots \\ {{{\hat {\varvec{c}}}_{{\hat {n}} - 1}}} \end{array}} \right],\quad \mathbf{P} = \left[ {\begin{array}{{c}} {{\varvec{c}_{0}}}\\ \vdots \\ {{\varvec{c}_{n - 1}}} \end{array}} \right],\quad {\rm{and}}\quad \mathbf{S} = \left[ {\begin{array}{{c}} {\varvec{x}_{0}^{s}}\\ \vdots \\ {\varvec{x}_{l}^{s}} \end{array}} \right],$$

representing the (unknown) control points of $\hat{\tau },$ the (known) control points of $\tau ,$ and a set of sampling points ${\varvec{x}}^{s}$ interpolated by both patches. The total number of control points involved is determined by the number of non-zero basis functions, i.e., $\hat{n}= ( \hat{p}+1 ) ( \hat{q}+1 )$ and $n= ( p+1 )( q+1).$ The number of sampling points, i.e., $l+1,$ is arbitrary but larger than $\hat{n}.$ The basis function values of $\hat{\tau }$ at ${\varvec{x}}^{s}$ are provided by

100 $${\hat {\mathbf{B}}} = \left[ {\begin{array}{{ccc}} {{{\hat {B}}_{0,\hat {p}}}(\hat {u}_{0}^{s}){{\hat {B}}_{0,\hat {q}}}(\hat {v}_{0}^{s})}& \cdots &{{{\hat {B}}_{\hat {p},\hat {p}}}(\hat {u}_{0}^{s}){{\hat {B}}_{\hat {q},\hat {q}}}(\hat {v}_{0}^{s})}\\ \vdots & \ddots & \vdots \\ {{{\hat {B}}_{0,\hat {p}}}(\hat {u}_{l}^{s}){{\hat {B}}_{0,\hat {q}}}(\hat {v}_{l}^{s})}& \cdots &{{{\hat {B}}_{\hat {p},\hat {p}}}(\hat {u}_{l}^{s}){{\hat {B}}_{\hat {q},\hat {q}}}(\hat {v}_{l}^{s})} \end{array}} \right],$$

and the corresponding values of $\tau$ are given by

101 $$\mathbf{B} = \left[ {\begin{array}{{ccc}} {{B_{0,p}}(u_{0}^{s}){B_{0,q}}(v_{0}^{s})}& \cdots &{{B_{p,p}}(u_{0}^{s}){B_{q,q}}(v_{0}^{s})}\\ \vdots & \ddots & \vdots \\ {{B_{0,p}}(u_{l}^{s}){B_{0,q}}(v_{l}^{s})}& \cdots &{{B_{p,p}}(u_{l}^{s}){B_{q,q}}(v_{l}^{s})} \end{array}} \right],$$

Note that a correlation between the parametric values has to be established so that ${\varvec{x}}^{s}_{i}=\tau ({u}^{s}_{i},\,{v}^{s}_{i}) = \hat{\tau }({\hat{u}}^{s}_{i},\,{\hat{v}}^{s}_{i}),\, i=0,\ldots ,l.$ The system of equations (98) is overdetermined consisting of $l+1$ equations and $\hat{n}$ unknowns. In general, it cannot be solved exactly, but a good approximation of the solution can be found by forming

102 $$\begin{aligned} {\hat{\mathbf {B}}}^{\intercal } {\hat{\mathbf {B}}} {\hat{\mathbf {P}}} = {\hat{\mathbf {B}}}^{\intercal } {\mathbf {S}} = {\hat{\mathbf {B}}}^{\intercal } {\mathbf {B}} {\mathbf {P}}, \end{aligned}$$

which yields the definition of the transformation matrix

103 $$\begin{aligned} {\mathbf {T}} = \left( {\hat{\mathbf {B}}}^{\intercal } {\hat{\mathbf {B}}}\right) ^{-1} {\hat{\mathbf {B}}}^{\intercal } {\mathbf {B}}, \end{aligned}$$

and the relation between the control points

104 $$\begin{aligned} {\hat{\mathbf {P}}} ={\mathbf {T}}{\mathbf {P}}. \end{aligned}$$

As a result, numerical integration can be performed based on the regular reconstruction patch $\hat{\tau }$ and the simple mapping of the regular integration can be applied. The values obtained are distributed to the control points of the original patch using the transformation matrix ${\mathbf {T}}.$ For more details, the interested reader is referred to [260].

This procedure can be directly applied to cut elements of types 3 and 4 as specified in Sect. 5.3.1. Type 5 elements may be subdivided into two four-sided regions. A drawback of the local reconstruction scheme is that it introduces an additional approximation error since the system of equations (102) cannot be solved exactly. Moreover, the stability of the computation of the transformation matrix might be affected if only a very small region of a cut element needs to be reconstructed.

##### Approximated Trimming Curve

The following two schemes approximate the trimming curve ${\varvec{C}}^{t}$ in order to define proper integration points within the parameter space. One uses a linear approximation of ${\varvec{C}}^{t}$ to set up a tailored integration rule, whereas the other applies an adaptive subdivision to approximate the domain of the cut element.

**Fig. 53:**
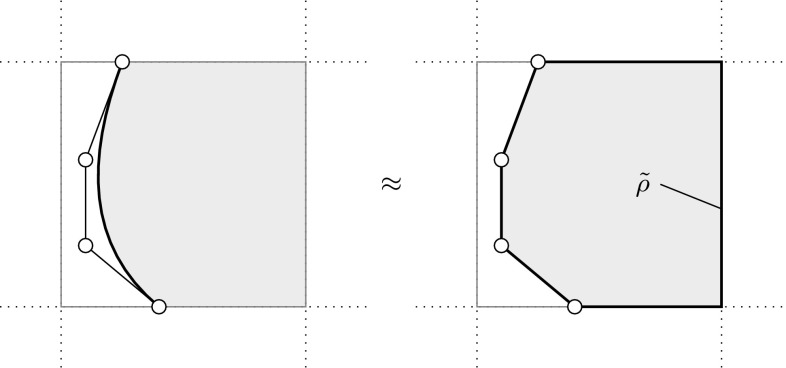
Approximation of the cut element by a polytope ${\tilde{\rho} }.$ The control points of the trimming curve are marked by *circles*

A *tailored integration* rule can be established for each cut element ${\tilde{\tau} }$ as proposed in [211, 305, 306]. The control polygon^20^ of ${\varvec{C}}^{t}$ is used to represent ${\tilde{\tau} }$ by a polytope ${\tilde{\rho} }$ as shown in Fig. 53. The integral over ${\tilde{\rho} }$ can be reduced to a sum of line integrals over the edges of ${\tilde{\rho }}$ using Lasserre’s theorems [183]. Therefore, the integration domain ${\Omega _{\tilde{\rho }}}$ has to be convex. Thus, a preprocessing step is applied to represent non-convex regions by a combination of convex ones. The line integrals provide reference solutions for the right-hand side of a set of moment-fitting equations given by

105 $$\begin{aligned} \sum _{i=1}^{m} f_{j}(u_i,\,v_{i}) w_{i}&= \int _{\Omega _{\tilde{\rho }}} f_{j}(u,\,v) \;\text {d}{\Omega _{\tilde{\rho }}},&j=1,\ldots ,n. \end{aligned}$$

They are used to computed a tailored quadrature rule, i.e., points ${\varvec{y}}_{i}=(u_{i},\,v_{i})^{\intercal }$ and weights $w_{i},$ for all functions $f_{j}$ of the desired functions space, e.g., monomials up to a certain degree. The goal is to find the lowest number of ${\varvec{y}}_{i}$ so that the Eq. (105) are satisfied up to a certain tolerance, for each ${\tilde{\tau }}$ or rather ${\tilde{\rho} }.$ The algorithm proposed in [211] starts with an initial set of ${\varvec{y}}_{i}$ and successively eliminates one superfluous point after another. In each step, the reduced set of points is used to solve the system of equations (105) in the least squares sense.

The construction of a tailored quadrature has two main benefits: (i) the number of integration points per ${\tilde{\tau} }$ is optimized and (ii) *all* cutting patterns are covered by a single technique, including cases which had been labeled as invalid in Sect. 5.3.1. Of course, this comes at the price of a more involved preprocessing phase since every cut element has to be treated individually. Furthermore, an error is introduced due to the approximation of ${\tilde{\tau} }$ by ${\tilde{\rho} }.$ This error can be reduced by refinement of the trimming curve since the control polygon converges to it. Still, the smooth higher degree representation is removed by a linear one. Finally, it should be pointed out that the reduction of integration points does not take the smoothness of the basis into account, as it is done in case of optimized quadrature rules for regular splines, see e.g., [118].

**Fig. 54:**
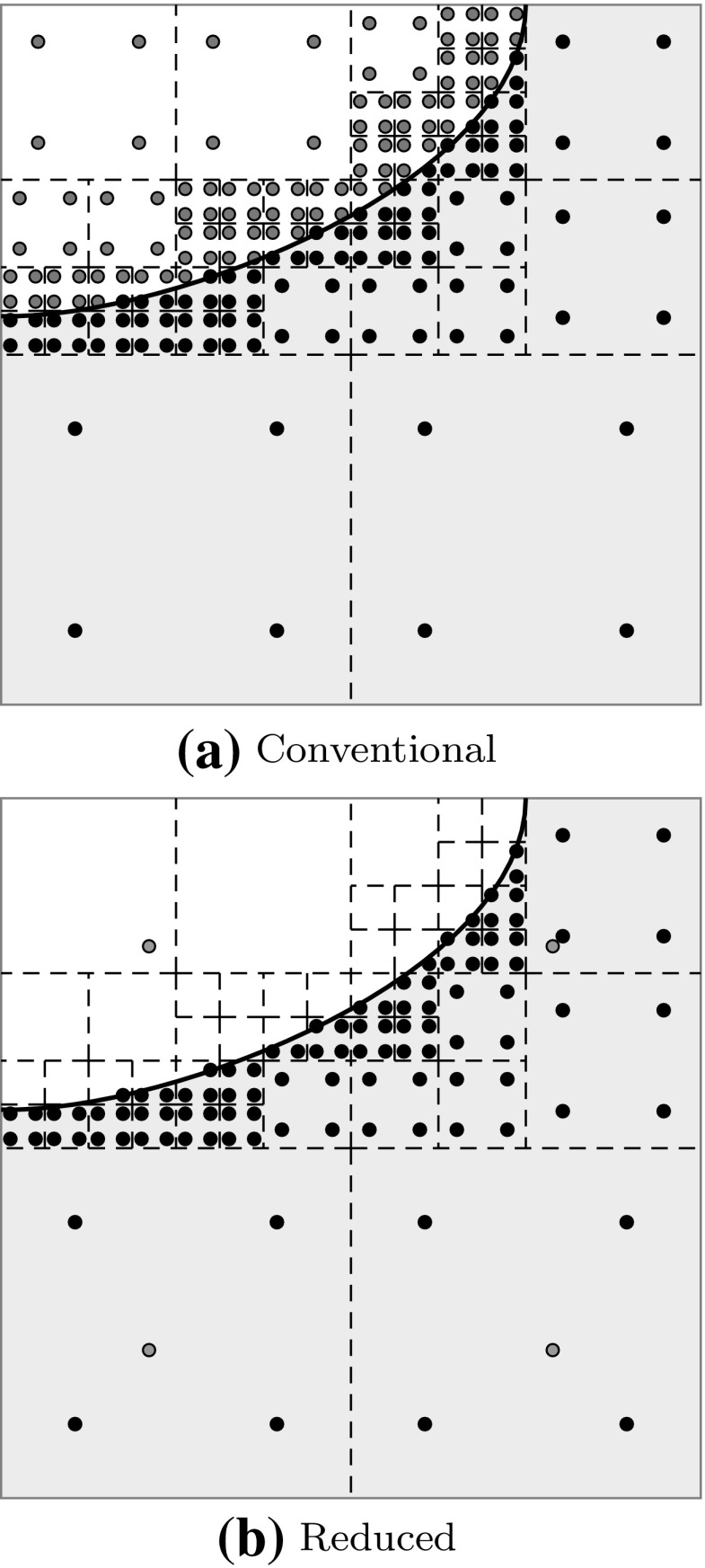
Sub-cell structure of a single cut element: **a** conventional approach with quadrature points distributed within the valid (*black points*) and exterior (*green points*) domain and **b** reduced approach integrating over the whole element (*orange points*) and the valid domain (*black points*). The sub-cells are indicated by *dashed lines*. (Color figure online)

A completely different strategy for the integration of cut elements in based on *adaptive subdivision*. Researchers who developed the *finite cell method* applied this technique to trimmed geometries [107, 239, 253, 254]. The basic idea is to use a composed Gauss quadrature that aggregates integration points along the trimming curve. A cut element ${\tilde{\tau} }$ is decomposed into axis-aligned sub-cells ${\tilde{\tau} }^{\boxplus }$ based on a tree-structure, i.e., a quadtree in two dimensions. Starting from the initial cut element, each sub-cell is further subdivided into equally spaced sub-cells if it contains the trimming curve as displayed in Fig. 54a. This recursive procedure is performed up to a user-defined maximal depth. Following the spirit of fictitious domain methods the integral $I^{c}$ over the complete element is defined as

106 $$\begin{aligned} I^{c} = \sum _{i=1}^{I} I^{\text{v}}_{{\tilde{\tau }}^{\boxplus }_{i}}(\alpha^{\text{v}}) + \sum _{j=1}^{J} I^{-}_{{\tilde{\tau }}^{\boxplus }_{j}}(\alpha ^{-}). \end{aligned}$$

The factors $I^{\text{v}}_{{\tilde{\tau} }^{\boxplus }_{i}}$ and $I^{-}_{{\tilde{\tau} }^{\boxplus }_{j}}$ are the integrals over the valid domain ${\mathcal {A}}^{\text{v}}$ and the complementary exterior domain ${\mathcal {A}}^{-},$ respectively. Integration points in the interior of ${\mathcal {A}}^{\text{v}}$ are multiplied by $\alpha ^{\text{v}}=1,$ whereas exterior integration points are multiplied by a value that is almost zero, e.g., $\alpha ^{-}=10^{-14}$ as suggested in [253]. The integration procedure can be improved with respect to the number of quadrature points by

107 $$\begin{aligned} I^{c} = I^{-}_{{\tilde{\tau }}}(\alpha ^{-}) + \sum _{i=1}^{I} I^{\text{v}}_{{\tilde{\tau} }^{\boxplus }_{i}}(\alpha ^{\text{v}}-\alpha ^{-}), \end{aligned}$$

where $I^{-}_{{\tilde{\tau} }}(\alpha ^{-})$ represents the integral over the whole cut element without taken the trimming curve into account. The integration over the valid domain is performed as before by the composite quadrature, yet with another weighting factor, i.e., $(\alpha ^{\text{v}}-\alpha ^{-}).$ Such an improved sub-cell integration is illustrated in Fig. 54b.

The key features of this approach are its simplicity and generality. The definition of integral transformation ${\mathcal {X}}_{r}$ and its Jacobian is straightforward, due to the axis-aligned shape of the sub-cells. Again, *all* cutting patterns (including invalid ones) can be addressed with a single algorithm. Moreover, the algorithm can be easily extended to higher dimensions. The downside is that the trimming curve is only approximated. Consequently, the integration region is not represented exactly and an additional approximation error is introduced. In fact, the accuracy of the integral ceases at a certain threshold [173, 175]. This threshold may be improved by the subdivision depth, but a fine resolution of sub-cells results in a vast number of quadrature points. Further, refined sub-cells do not converge to the trimming curve in contrast to the previous approach. One of the great successes of the finite cell method was the demonstrated ability to achieve higher rates of convergence for higher-order elements and splines, and even exponential rates in the context of the *p*-method.

##### Exact Trimming Curve

The following techniques focus on defining a proper mapping ${\mathcal {X}}_{r}$ from the reference element $\grave{\tau }$ to the cut element ${\tilde{\tau} }\in {\mathcal {A}}^{\text{v}}$ so that the trimming curve is exactly represented. Depending on the cutting pattern, ${\tilde{\tau} }$ may be represented by a disjointed set of integration regions ${\tilde{\tau} }^{\boxdot }$ such that

108 $$\begin{aligned} {\tilde{\tau} }= \bigcup _{i=1}^{I} {\tilde{\tau} }^{\boxdot }_{i}. \end{aligned}$$

In contrast to the sub-cells of the previous scheme, the regions ${\tilde{\tau} }^{\boxdot }$ are not aligned with the axes of the parameter space and at least one ${\tilde{\tau} }^{\boxdot }$ has an edge which is described by the portion of the trimming curve ${\varvec{C}}^{t}$ within ${\tilde{\tau} }.$

**Fig. 55:**
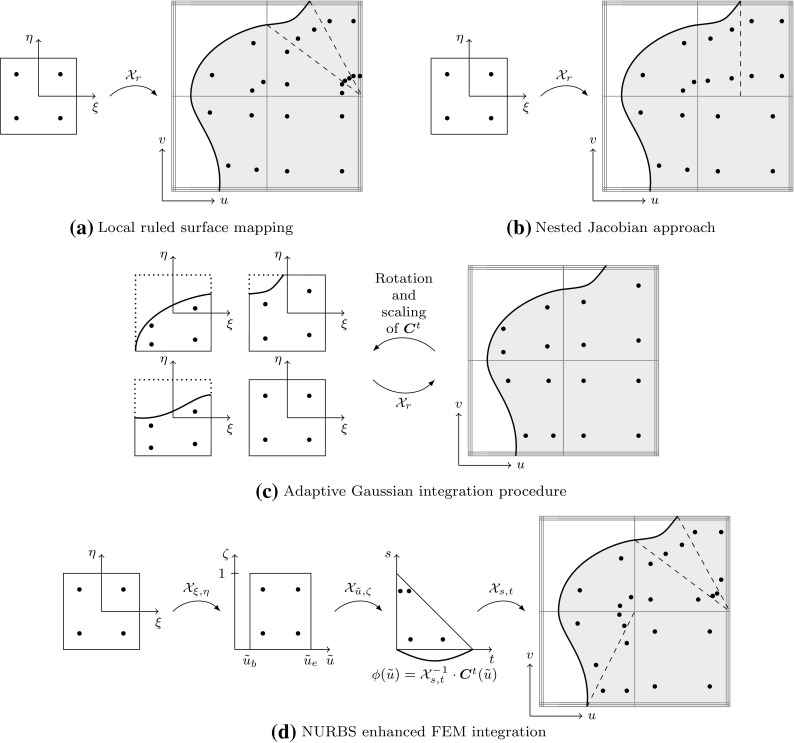
Distribution of quadrature points due to various approaches which represent the trimming curve exactly. *Dashed lines* indicate a subdivision of a cut element into integration regions

There are various ways to specify ${\mathcal {X}}_{r}.$ Ruled surface (26) and Coons patch (35) interpolation may be applied, where the portion of the trimming curve within ${\tilde{\tau} }$ is considered for the construction [199, 307]. An example of local ruled surface mappings for various element types are shown in Fig. 55a. These methods may be interpreted as local counterparts of the global reconstruction schemes presented in Sects. 5.2.1 and  5.2.2. It is worth noting that approaches based on the *blending function method* [95, 173, 174] can be included into this category, because this method also employs a transfinite mapping [103]. In the *nested Jacobian approach*, integral transformation is also defined by a local NURBS surface combined with a nested subdivision [38, 227]. Thus, ${\mathcal {X}}_{r}$ consists of the local surface mapping and an additional transformation to the subregion. A corresponding distribution of quadrature points is shown in Fig. 55b. In contrast to both previous references, i.e., [199, 307], type 5 elements are not decomposed into three triangular ones, but a bisection of the knot span is performed. Recently, an *adaptive Gaussian integration procedure* has been proposed [37]. This variation of the nested Jacobian approach defines the local surface parameterization within the reference space instead of the trimmed parameter space as illustrated in Fig. 55c. Therefore, the trimming curve is transformed to the reference space by scaling and rotation. The integration points and their weights are adapted by scaling the $\eta$-direction such that the points are located within the region described by the transformed trimming curve. The motivation for the adaptive Gaussian integration procedure is to treat the various cutting patterns by a single approach.

Another very common strategy is to adopt the integration scheme developed in the context of the *NURBS-enhanced finite element method* [147, 148, 160, 161, 272, 273]. Using this scheme, every cut element is subdivided into a set of triangles. Those triangles that only consist of straight edges are subjected to conventional integration rules for linear triangles. The other triangles are treated by a series of mappings that take the curved edge into account

109 $$\begin{aligned} {\mathcal {X}}_{r}:= {\mathcal {X}}_{s,t} ( {\mathcal {X}}_{{\tilde{u}},\zeta } ( {\mathcal {X}}_{\xi ,\eta } (\xi ,\eta ) ) ). \end{aligned}$$

Figure 55d displays the components of this series. Suppose the corner nodes of the triangle in the trimmed parameter space are labeled ${\varvec{x}}^{\vartriangle }_{1}$ to ${\varvec{x}}^{\vartriangle }_{3},$ where the beginning and the end of the trimming curve portion within the considered triangle are denoted by ${\varvec{x}}^{\vartriangle }_{2}$ and ${\varvec{x}}^{\vartriangle }_{3},$ respectively. The transformation ${\mathcal {X}}_{s,t}{\text {:}}\,{\varvec{x}}(s,\,t) \mapsto {\varvec{x}}(u,\,v)$ describes the mapping of a linear three node element

110 $$\begin{aligned} {\mathcal {X}}_{s,t} := {\varvec{x}} (u,\,v) = t{\varvec{x}}^{\vartriangle }_{1} + (1-s-t) {\varvec{x}}^{\vartriangle }_{2} + s{\varvec{x}}^{\vartriangle }_{3}. \end{aligned}$$

In order to address the curved edge, the trimming curve is transformed into the $s{,}\,t$-coordinate system by

111 $$\begin{aligned} \phi ({\tilde{u}})&={\mathcal {X}}_{s,t}^{-1} \cdot {\varvec{C}}^{t}({\tilde{u}}) \nonumber \\&= {\left[ {\begin{array}{{cc}} {x_{3}^{\Updelta} - x_{2}^{\Updelta}}&\quad{x_{1}^{\Updelta} - x_{2}^{\Updelta}} \end{array}} \right]^{ - 1}}\left( {{C^t}(\tilde {u}) - x_{2}^{\Updelta}} \right). \end{aligned}$$

The next mapping ${\mathcal {X}}_{{\tilde{u}},\zeta }{\text {:}}\,{\varvec{x}}({\tilde{u}},\,\zeta ) \mapsto {\varvec{x}}(s,\,t)$ converts the triangular domain into a rectangular one which possesses straight edges only. It is given by

112 $$\begin{aligned} {\mathcal {X}}_{{\tilde{u}},\zeta }:= \left\{ \begin{array}{rcl} s&{}=\phi _{s}({\tilde{u}}) &{}( 1 - \zeta ), \\ t&{}=\phi _{t}({\tilde{u}}) &{}( 1 - \zeta ) + \zeta . \end{array} \right. \end{aligned}$$

Finally, the transformation ${\mathcal {X}}_{\xi ,\eta }{\text {:}}\,{\varvec{x}}(\xi ,\,\eta ) \mapsto {\varvec{x}}({\tilde{u}},\,\zeta )$ of the reference space $[-1,\,1]^{2}$ to the rectangular region is performed by

113 $$\begin{aligned} {\mathcal {X}}_{\xi ,\eta }:= \left\{ \begin{array}{rl} {\tilde{u}}&{}= \frac{\xi }{2}( {\tilde{u}}_{e} - {\tilde{u}}_{b} ) + \frac{1}{2}( {\tilde{u}}_{e} + {\tilde{u}}_{b} ) \\ \zeta &{}= \frac{\eta }{2} + \frac{1}{2}, \end{array} \right. \end{aligned}$$

where ${\tilde{u}}_{b}$ and ${\tilde{u}}_{e}$ are the parametric values of the beginning and the end of the trimming curve portion within the triangle, i.e., ${\varvec{C}}^{t}({\tilde{u}}_{b}) = {\varvec{x}}^{\vartriangle }_{2}$ and ${\varvec{C}}^{t}({\tilde{u}}_{e}) = {\varvec{x}}^{\vartriangle }_{3}.$ The Jacobian determinant of the overall mapping ${\mathcal {X}}_{r}$ is determined by

114 $$\begin{aligned} J_{{\grave{\tau }}}= \det \left( {\mathbf {J}}_{s,t} \right) \det \left( {\mathbf {J}}_{{\tilde{u}},\zeta } \right) \det \left( {\mathbf {J}}_{\xi ,\eta } \right) , \end{aligned}$$

with

115 $${\mathbf{J}_{s,t}} = \left[ {\begin{array}{*{20}{c}} {u_3^\Delta - u_2^\Delta }&\quad{u_3^\Delta - v_2^\Delta }\\ {u_1^\Delta - u_2^\Delta }&\quad{v_1^\Delta - v_2^\Delta } \end{array}} \right],$$

116 $${\mathbf{J}_{\tilde u,\varsigma }} = \left[ {\begin{array}{{cc}} {\frac{{\partial {\phi _s}(\tilde u)}}{{\partial \tilde u}}(1 - \varsigma )}&{\frac{{\partial {\phi _t}(\tilde u)}}{{\partial \tilde u}}(1 - \varsigma )}\\ {-{\phi _s}(\tilde u)}&{1 - {\phi _t}(\tilde u)} \end{array}} \right],$$

117 $${\mathbf{J}_{\xi ,\eta }} = \left[ {\begin{array}{*{20}{c}} {\frac{1}{2}({{\tilde u}_e} - {{\tilde u}_b})}&0\\ 0&{\frac{1}{2}} \end{array}} \right].$$

The coefficients $u^{\vartriangle }_{i}$ and $v^{\vartriangle }_{i}$ refer to the coordinates of the corner nodes ${\varvec{x}}^{\vartriangle }_{i}$ and the derivative of the transformed trimming curve is calculated by

118 $$\frac{{\partial \phi (\tilde u)}}{{\partial \tilde u}} = {\left[ {\begin{array}{{cc}} {\varvec{x}_3^\Delta - \varvec{x}_2^\Delta }&\quad{\varvec{x}_1^\Delta - \varvec{x}_2^\Delta } \end{array}} \right]^{ - 1}}\left( {\frac{{\partial {\varvec{C}^t}(\tilde u)}}{{\partial \tilde u}}} \right).$$

**Fig. 56:**
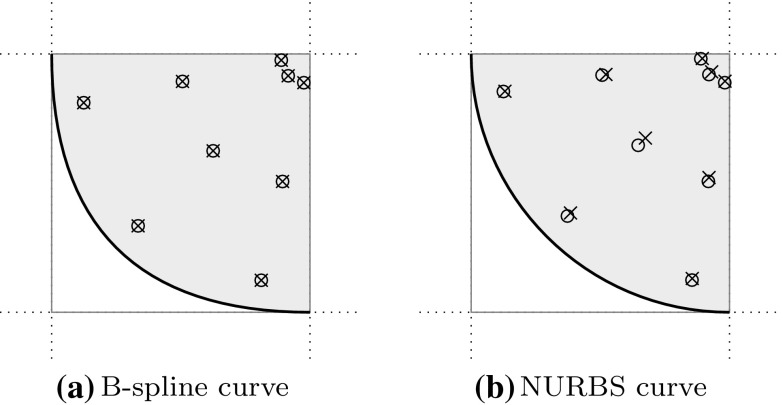
Comparison of the distribution of Gauss points within a cut element of type 3 based on the NURBS enhanced FEM mapping (*circles*) and ruled surface parameterization (*crosses*). The trimming curve is described by either **a** a B-spline curve or **b** a NURBS curve

The various integration schemes are summarized in Fig.55. Their common and most essential feature is that the integration region is exactly represented. The main difference between the strategies is the partitioning of a cut element ${\tilde{\tau }}$ into integration regions ${\tilde{\tau} }^{\boxdot }.$ In fact, the series of mappings (109) shown in Fig. 55d yields the same distribution of quadrature points over a triangular element as a ruled surface interpolation (26) illustrated in Fig. 55a, if the trimming curve is a B-spline curve. In case of NURBS curves, on the other hand, different distributions are obtained. These two cases are compared in Fig. 56.

In general, it seems that good results can be obtained with either of these concepts, especially for moderate degrees. However, it has been demonstrated that the properties of coordinate mappings and the corresponding placement of interior nodes is crucial for the convergence behavior of conventional higher degree ($p>3$) finite elements [216]. With this in mind, additional research might be useful to assess the quality of the mapping schemes presented with respect to their performance for higher degree.

#### Multipatch Geometries

A robust treatment of multiple patches is the most challenging part of analyzing trimmed geometries. While single patch analysis can exploit the benefits of trimmed representations, all the deficiencies elaborated in Sect. 3 surface as soon as solid models described by several trimmed patches are considered. In case of finite element methods, the main ingredients to cope with this situation are: (i) a weak coupling formulation and (ii) a robust procedure linking the degrees of freedom of adjacent patches to each other. This subsection closes with some general statements regarding the continuity of trimmed multipatch geometries.

##### Weak Coupling

Weak enforcement of constraints is a common problem in computational mechanics, see e.g., [138, 166, 312] and the references cited therein. Such techniques are required in several contexts like mesh-independent imposing of essential boundary conditions and domain decomposition methods. The latter covers a versatile field of applications including contact problems, parallelization, and coupling of subdomains described by different physics or non-conforming discretizations. Numerous approaches have been developed and each one possesses different benefits and disadvantages. The most popular schemes are based on Lagrange multipliers [12], the penalty method [13, 139], or Nitsche’s method [112, 215]. These methods are separated by a fine line: the penalty method may be viewed as an approximation of the Lagrange multiplier method [139]. Furthermore, the Nitsche method may be referred to as a consistent penalty method [252]. In addition, the close relationship of the Nitsche method to the stabilized Lagrange multiplier method [17, 18] has been outlined in [288].

The use of Lagrange multipliers is a very general way to enforce constraints to a system of equations which is applicable to all kinds of problems. Following Huerta et al. [138] the main disadvantages are: (i) the system of equations increases due to the Lagrange multipliers which are incorporated as additional degrees of freedom, (ii) the resulting system is not positive definite, and (iii) the introduction of a separate field for the Lagrange multipliers yields a saddle-point problem which must satisfy a stability condition known as the inf–sup or Babuška–Brezzi condition. In order to fulfill the last point, the interpolation fields of the unknowns and the Lagrange multipliers must be coordinated, which is not a trivial task, examples of choices for the interpolation functions can be found in [139].

The penalty method is easy to implement and avoids the problems mentioned above. However, uniform convergence to the solution can only be guaranteed if the applied penalty parameter increases as the mesh is refined [7]. This is crucial since the system matrix becomes ill-conditioned when the penalty parameter gets large. Usually, a fixed parameter value is chosen and as a result, the quality of the approximation cannot be improved below a certain error.

Nitsche’s method introduces a penalty term too, but it is considerably smaller than in the penalty method [80, 254]. According to Huerta et al. [138] the only problem of Nitsche’s method is that it is not as general as the other procedure. Thus, it is not straightforward to provide an implementation for some problem types.

These techniques have been successfully applied to various isogeometric analysis applications, e.g. [23, 24, 39, 80, 81, 163, 213]. A comparison of the three schemes can be found in [7]. Also in the context of coupling trimmed patches, the Lagrange multiplier method [307], the penalty method [37, 38], and the Nitsche method [107, 164, 254] have been successfully applied already. In none of these publications, the surface type motivated the choice of the weak coupling strategy. In other words, trimmed patches do not introduce additional arguments to prefer one approach to the other. Nevertheless, it is important to emphasize again that trimming curves of adjacent patches do *not* describe the same curve in model space. Thus, adjacent patches have non-conforming parameterizations as well as gaps and overlaps along their intersection.

##### Linking of Degrees of Freedom

**Fig. 57:**
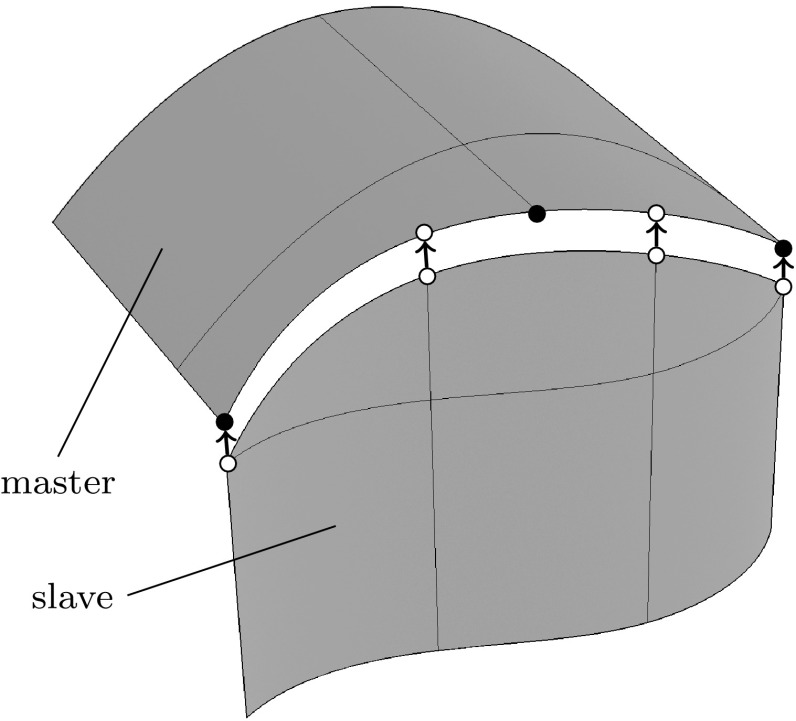
Closest point projections of a slave patch to a master patch. The *lines* on the surfaces represent the grid of the underlying parameter space. The intersections of these *lines* with the trimming curves are illustrated by *white* and *black dots* for the slave and master patch, respectively. The projections themselves are indicated by *arrows*

Breitenberger et al. [38] presented a procedure that is able to deal with complex design models and it has been discussed in more detail in the related thesis [37]. In addition to a weak coupling formulation, trimming curves of adjacent patches are connected by so-called edge elements that contain the required topological information. To be precise, the trimming curves are treated by a *master*–*slave* concept where points of the slave curve are mapped to the master curve. These points are the intersections of the slave trimming curve with the grid lines of its own parameter space. The mapping to the master curve is performed in model space by means of a *point inversion algorithm* [192, 230]. The algorithm is usually carried out by a Newton–Raphson scheme and provides the closest projection of a point to a curve as shown in Fig. 57. In addition, the related parametric values of the master curve are provided by the point inversion scheme. The accumulation of these values and the original grid intersections of the master curve define a set of integration regions. Within each region, quadrature points are specified and the corresponding points of the slave curve can again be computed by the point inversion algorithm. To sum up, the relation of two related trimming curves is establish by an iterative procedure in model space which computes the shortest distance of a point defined by one curve to the other curve. This is indeed the same concept as for the knot cross-seeding procedures presented in Sect. 5.2 in the context of global approaches. In theory, this is a straightforward task, but its robust implementation is challenging and crucial for the overall performance of an analysis.

**Fig. 58:**
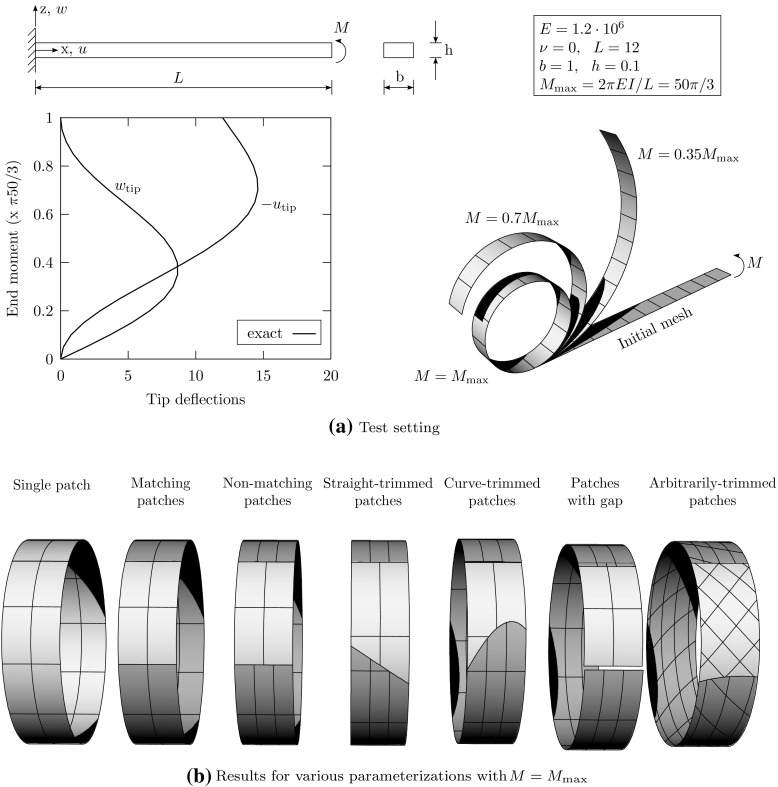
Different geometry models for analyzing a cantilever subjected to an end moment: **a** definition of the problem and **b** resulting solutions. In **b**, different *gray scales* indicate the distinct patches. Note the various complexities of the connection of adjacent patches. (Courtesy of Breitenberger [37, 38])

In the following we would like to highlight the importance of a robust association of adjacent patches by showing an example presented in [37, 38]. The basic setting of the problem is shown in Fig. 58a. This benchmark for geometric nonlinear shell analysis describes a cantilever that is subjected to an end moment. If the maximal moment $M_{max}$ is applied, the cantilever deforms to a closed circular ring. Figure 58b illustrates the numerical solution of this problem for various parameterizations. Note the different level of complexity along the edges of adjacent patches. It clearly demonstrates the vast diversity of situations that my occur in case of multipatch geometries even if they represent the same geometry.

**Fig. 59:**
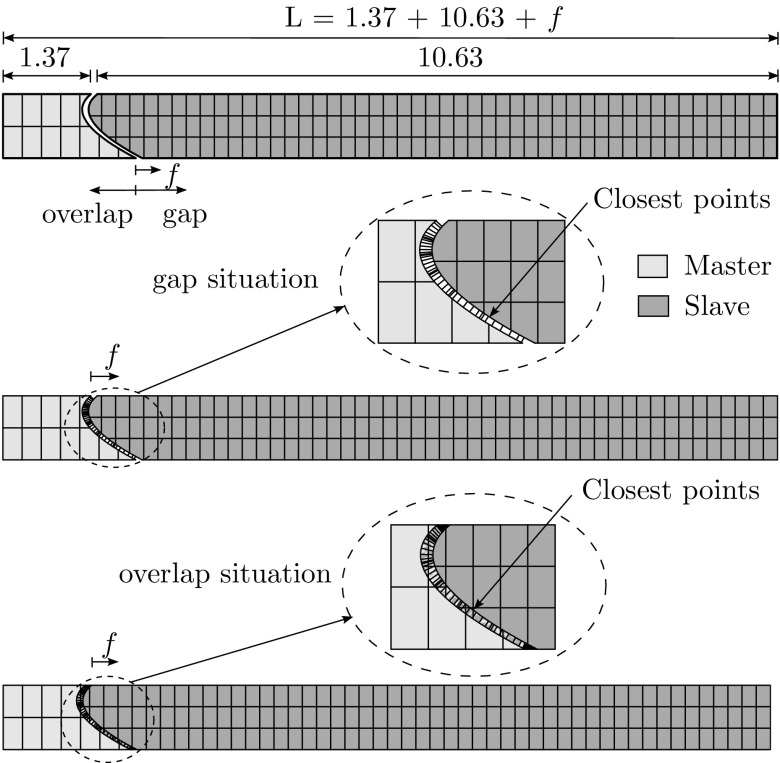
Geometry representation and definition of the gap–overlap parameter *f* for the investigation of the effect of non-watertight geometries on numerical results. (Courtesy of Michael Breitenberger [37, 38])

**Fig. 60:**
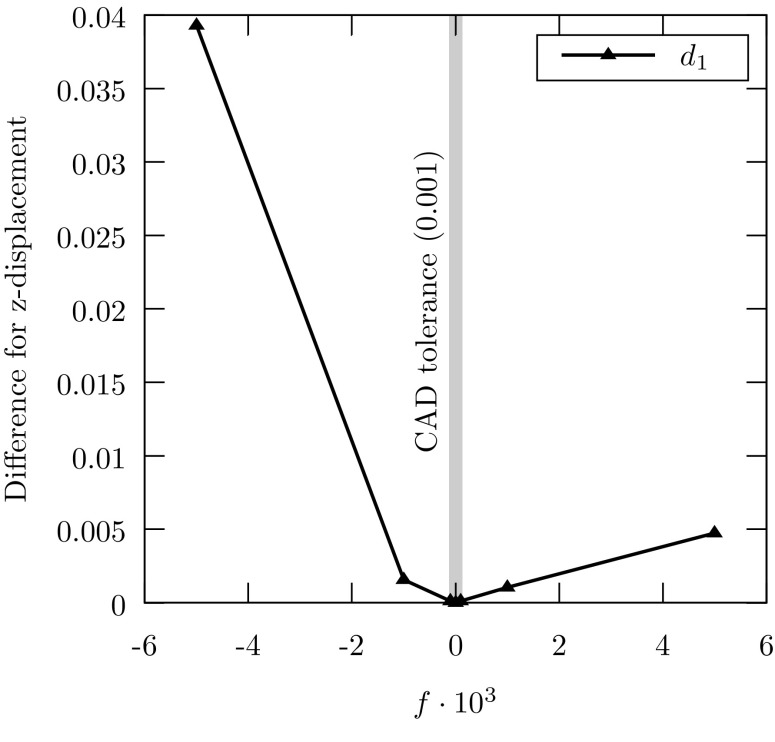
Comparison of the relative vertical displacement $d_{1}$ related to the gap–overlap parameter *f*. Gaps and overlaps are indicated by positive and negative values, respectively. The *gray area* of the diagram indicates the default tolerance of the CAD software used. (Courtesy of Breitenberger [37, 38])

Another important aspect studied by this example is the influence of the gap and overlap size between patches. Consider the geometrical discretization illustrated in Fig. 59. A gap–overlap function *f* is introduced to specify a user-defined inaccuracy along the curved intersection. Positive and negative values of *f* represent gaps and overlaps, respectively. The trimming curves are linked by the point inversion algorithm as described before. The resulting vertical displacements at the cantilever’s end $u_{Tip}$ of representations with different *f* are related to a reference solution $u_{ref}$ obtained with $f=0.$ The difference is calculated by

119 $$\begin{aligned} d_{1}=\left| u_{Tip}(f) - u_{ref}\right| , \end{aligned}$$

and the related results are summarized in Fig. 60. Based on the corresponding graph, it can be concluded that small gaps which are within CAD tolerance, i.e., 0.001 units, barely influence the quality of the simulation. The different behavior of gaps and overlaps can be explained by the minimal distance computation: in contrast to gaps, the assignment of points of the slave curve to the master curve is not unique in case of overlaps.

##### Continuity Considerations

The continuity along the intersection of two trimmed patches is usually not higher than $C^{0}.$ The construction of corresponding $C^{0}$ isogeometric spaces with optimal approximation properties is well understood for conforming parameterizations [299]. Brivadis et al. [39] showed this also for weakly imposed $C^{0}$ conditions. However, their isogeometric mortar method focuses on regular patches and a modification of the basis functions at the boundary is required to obtain stability, if the same degree is used for the primal and dual spaces. Although the influence of non-matching interfaces is discussed as well, the application in the context of trimmed surfaces has yet to be investigated in more detail.

The construction of smooth isogeometric spaces for trimmed models is an even more complicated open topic. In fact, smooth isogeometric spaces on unstructured geometries are a challenging and open problem in general [141, 296]. Locking effects may occur even for regular planar multipatch configurations [63, 150]. At this point, it should be noted that T-splines or subdivision surfaces provide geometric models which are globally smooth almost everywhere. Nevertheless, these representations seem to lack optimal approximation properties due to the existence of extraordinary vertices [145, 212].

#### Stabilization

**Fig. 61:**
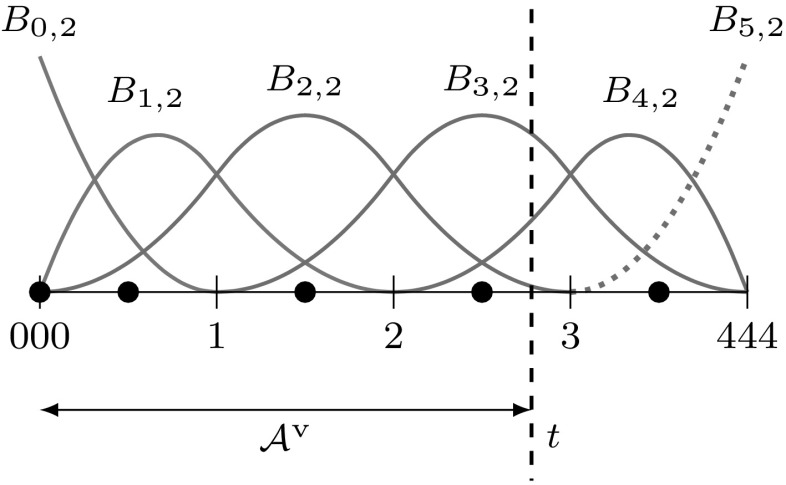
Univariate basis trimmed at a parameter $t.$ There are basis functions which are fully inside (*green*), partially inside (*blue*), and completely outside (*dotted*) of ${\mathcal {A}}^{\text{v}}.$ The Greville abscissae of the considered basis functions are marked by *circles*. (Color figure online)

A trimmed basis contains basis functions which are cut by the trimming curve and exist only partially within the valid area ${\mathcal {A}}^{\text{v}}.$ In order to clarify the problem statement, Fig. 61 illustrates a trimmed univariate basis. It should be noted that the Greville abscissae of cut basis functions may be located outside of ${\mathcal {A}}^{\text{v}}.$ In the example given, this is the case for $B_{4,2}.$ These points cannot be used for collocation or spline interpolation problems, despite the fact that they are the preferred choice for setting up a stable system of equations (see Sect. 2.3). Furthermore, the support of cut basis functions may be arbitrary small, e.g., this would be the case for $B_{4,2}$ as the trimming location $t$ approaches the knot value 2. Thus, the condition number of the resulting system matrices can become very large. In other words, a trimmed basis is not guaranteed to be stable.

**Fig. 62:**
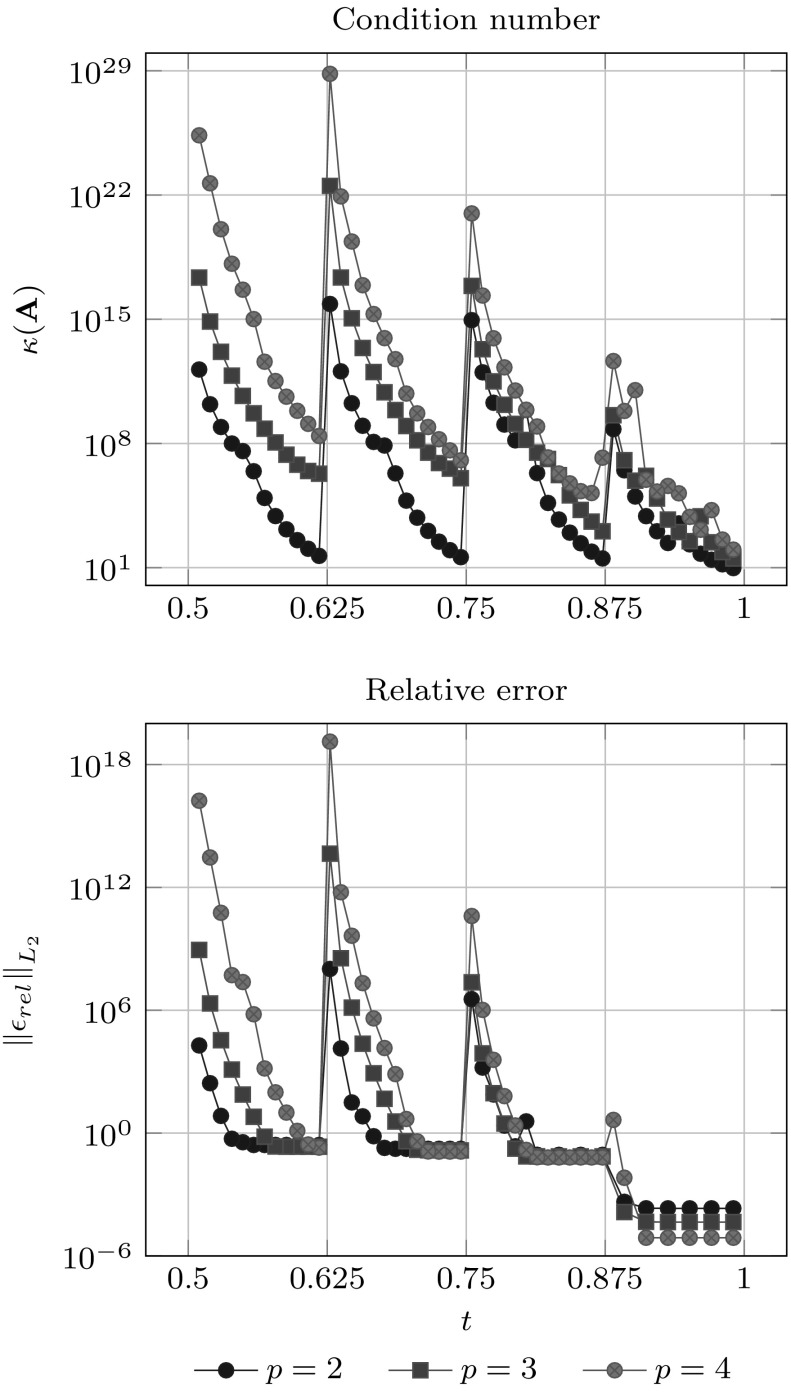
Condition number $\kappa ({\mathbf {A}})$ and relative interpolation error $\left\Vert \epsilon _{rel} \right\Vert_{L_{2}}$ of the bivariate basis for several degrees $p$ in both parametric directions related to the trimming parameter $t.$ The subdivision of the *horizontal axis* corresponds to the knot values of the trimmed basis

In order to emphasize this stability issue an interpolation problem is examined: a given function

120 $$\begin{aligned} f(u,\,v)&= \frac{1}{\sqrt{ \left( -1.2-u\right) ^{2} + \left( -1.2-v\right) ^{2} }}, \end{aligned}$$

shall be approximated by a B-spline surface ${\varvec{S}}_{h}.$ They agree at *k* interpolation points ${\bar{\varvec{x}}}_{i,j}=(\bar{u}_{i},\,\bar{v}_{j})^{\intercal },$ where *k* represents the total number of bivariate basis functions involved. The further components of the corresponding system of equations are the unknown coefficients $c_{i,j}$ and the bivariate spline collocation matrix ${\mathbf {A}}.$ The matrix is defined by

121 $$\begin{aligned}&{\mathbf {A}} [i+j\cdot J,\,m+n\cdot J] = B_{i,p}\left( \bar{u}_{m}\right) B_{j,q}\left( \bar{v}_{n}\right) , \end{aligned}$$

with $i,\,m=0,\ldots ,I$ and $j,\,n=0,\ldots ,J,$ where $I$ and $J$ are the number of basis functions in each parametric directions. The initial parameter space is given by an open knot vector with a uniform discretization from −1 to 1 in both directions, i.e., $u,\,v\in [-1,\,1],$ and the knot span size is specified by $h = 0.125.$ A trimming parameter $t\in [0.5,\,1)$ determines the square domain ${\mathcal {A}}^{\text{v}}\in [-1,\,t]^{2}$ considered for the interpolation problem. The interpolation points ${\bar{\varvec{x}}}$ of cut basis functions may have to be shifted into ${\mathcal {A}}^{\text{v}}.$ Exterior basis functions that are completely outside of ${\mathcal {A}}^{\text{v}}$ are not involved in the interpolation process. The quality and stability of the approximation ${\varvec{S}}_{h}$ are specified by the relative interpolation error measured in the $L_{2}$-norm $\left\Vert \epsilon _{rel} \right\Vert_{L_{2}}$ as well as the condition number of the spline collocation matrix $\kappa ({\mathbf {A}}).$ The results are summarized in Fig. 62 for various degree with $p= q.$

It can be observed that the condition number of ${\mathbf {A}}$ is considerably influenced by the trimming parameter $t.$ In particular, a peak is reached as soon as $t$ approaches a knot value, i.e., a support of cut basis functions becomes very small. Furthermore, the approximation quality is affected. The peaks of the relative error $\left\Vert \epsilon _{rel} \right\Vert_{L_{2}}$ near knot values are in fact disastrous. Hence, it is evident that the straightforward application of a trimmed basis negatively affects the condition number and subsequently the quality of the approximation.

The stability aspect of local approaches for the analysis of trimmed geometries has scarcely been considered in previous works. It is worth noting that Nitsche formulations may incorporate parameters which take cut elements into account, see e.g., [42, 80, 289]. A method-independent alternative that exploit the properties of B-splines is outlined in Sect. 6.

### Summary and Discussion

Various approaches to incorporate trimmed geometries into an analysis have been described in this review. While Sect. 5.1 addresses an early attempt which combines trimmed patches with Lagrange interpolation, recent research is the focus of the subsequent Sects. (5.2) and (5.3). To recapitulate the findings of the current approaches: there are two fundamentally different philosophies to deal with trimmed models. One seeks to resolve the deficiencies of trimmed models by a reconstruction of the geometric representation. This is performed as a preprocessing step before the actual analysis. Since these procedures affect entire patches and their connection, they are referred to as global approaches in this work. The other philosophy is to accept the flaws of trimmed models, implying that the analysis has to be adaptable enough to cope with them. This capability is accomplished by treating the occurring trimming situations on the knot span level. Hence, we classify such techniques as local approaches.

Global approaches address the core of the problem and aim to solve it at its origin. In fact, they are similar to the remodeling schemes of CAGD outlined in Sect. 3.4. They share the same shortcomings such as an increased number of control points and the dependence on a four-sided domain if regular tensor product surfaces are used for the reconstruction. It can be argued that global approaches are more related to CAGD than analysis. Consequently, their success is also determined by the acceptance in the design community. However, a compelling global scheme could eventually lead to design models which can be directly applied not only to analysis but *all* downstream applications, which is the holy grail of the trimming problem.

Local approaches focus on enhancing the analysis and thus, may seem more feasible for researchers in the field of computational mechanics. In fact, the majority of the publications on isogeometric analysis of trimmed geometries employ such concepts. There is a close relation to fictitious domain, or immersed, methods since the trimmed parameter space is used as a background parameterization. Hence, similar challenges have to be addressed: (i) detection of elements cut be the trimming curve, (ii) special integration schemes for these elements, (iii) weak coupling of adjacent patches, and (iv) the stability issue induced by the trimmed basis. The main difference is that additional effort has to be made to associate the degrees of freedom of adjacent patches, keeping in mind that their intersections possess non-matching parameterizations, gaps, and overlaps. These distinct tasks are clearly separated from each other. For example, weak coupling is mandatory for finite element methods but may be neglected if a boundary element method is applied. Most researchers have drawn their attention to the integration of cut elements. The application of weak formulations has also been addressed by several authors. On the other hand, the stability of a trimmed basis and the robust association of adjacent patches are barely discussed in the literature, despite the fact that the latter task is crucial for the analysis of practical design models. Another issue of using a trimmed basis for the analysis is that the Greville abscissae of cut basis functions are not guaranteed to be located inside of the domain of interest. Consequently, an application to interpolation and collocation methods requires further considerations. However, the modular structure of local approaches is indeed a benefit compared to global approaches which require a self-contained concept which becomes more and more sophisticated with its capabilities.

## Stabilization of a Trimmed Basis

There are two reasons for presenting a distinct section on the stabilization of trimmed parameter spaces: first and foremost, we want to draw attention to this issue which has been scarcely discussed so far, and, in addition, some of our recent research is focused on this topic allowing a more detailed observation of it. The general problem statement has already been given in Sect. 5.3.4, where it has been demonstrated that basis functions cut by a trimming curve can yield ill-conditioned system matrices. Further, Greville abscissae of such basis functions may be outside of the valid domain and thus, they cannot be applied to methods which employ these points like isogeometric collocation [11, 258]. In order to identify the troublesome components, we classify the basis functions of a trimmed parameter space as stable, degenerate, or exterior. The support of the latter is completely outside of the valid domain ${\mathcal {A}}^{\text{v}}$ and hence, it can be neglected for the analysis. The distinguishing feature of the other types is that the Greville abscissae of stable B-splines are within ${\mathcal {A}}^{\text{v}}$ whereas the Greville abscissae of degenerate ones are outside of ${\mathcal {A}}^{\text{v}}.$

The following stabilization scheme resolves the issues induced by degenerate basis functions in a simple and flexible manner. The concept is referred to as *extended B-splines*. Originally, these splines have been developed by Höllig and co-workers in the context of a B-spline based fictitious domain method [124–127]. Here, the main aspects of extended B-splines are outlined based on the findings provided in [199, 202].

### Definition of Extended B-splines

**Fig. 63:**
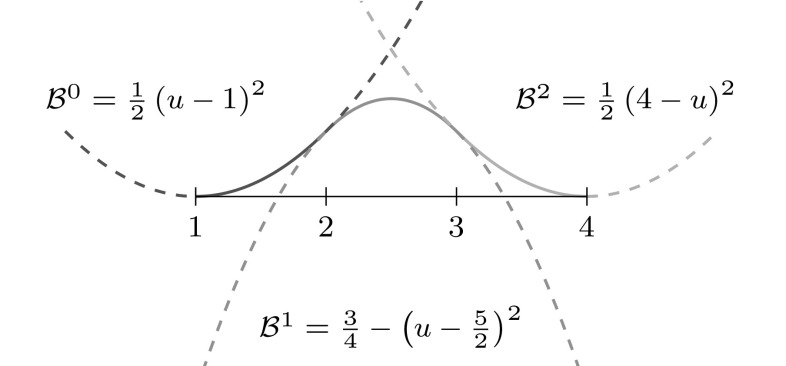
Polynomial segments ${\mathcal {B}}^{s}$ of a B-spline. The extensions of the segments are indicated by *dashed lines*

We start the description of extended B-splines by recalling two fundamental properties of conventional B-spline: (i) B-splines $B_{i,p}$ are represented by a set of polynomial segments ${\mathcal {B}}^{s}_{i}$ and (ii) B-splines form a basis of a space ${\mathbb {S}}_{p,\varXi }$ which contains every piecewise polynomial $f_{p,\varXi }$ of degree $p$ over a knot sequence $\varXi .$ The former property is illustrated in Fig. 63. It should be noted that each polynomial segment ${\mathcal {B}}^{s}$ may be *extended* beyond its associated knot span $s.$ With this in mind, it is straightforward to grasp the essential idea of extended B-splines, namely to re-established the stability of a trimmed basis by substituting degenerate, and therefore potentially unstable, B-splines by extensions of stable ones. These extensions can be exactly represented by the basis since they are within ${\mathbb {S}}_{p,\varXi }$ by definition.

**Fig. 64:**
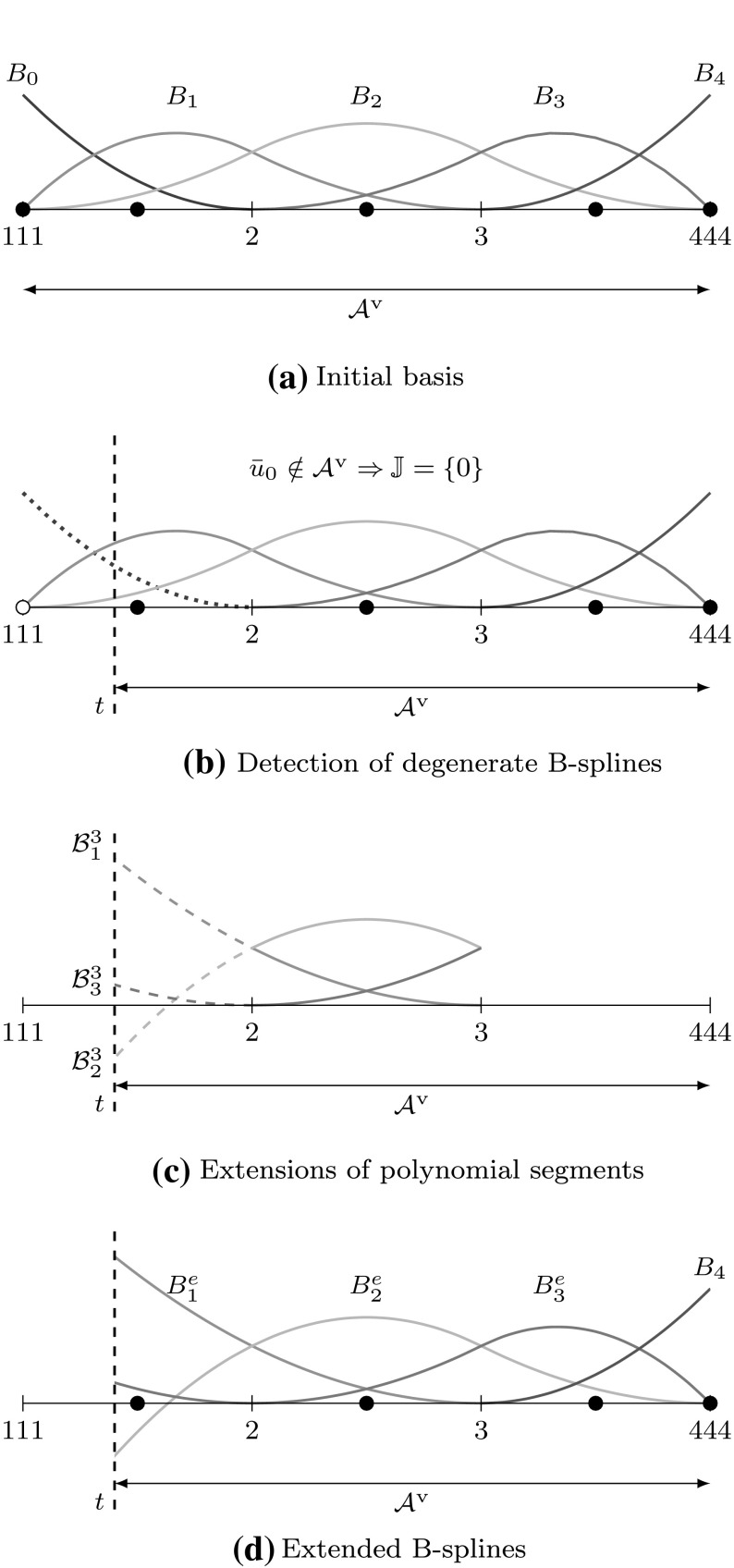
Basic procedure to get from **a** conventional to **d** extended B-splines: **b** determination of degenerate B-splines and substitution of trimmed polynomial segments by **c** extensions of non-trimmed ones

The overall construction procedure of extended B-splines is summarized in Fig. 64. Firstly, it is determined if the Greville abscissae of non-exterior B-splines are located inside or outside of ${\mathcal {A}}^{\text{v}}.$ In the latter case the basis function is labeled as degenerate and the corresponding index is stored in the index-set ${\mathbb {J}}.$ Secondly, the polynomial segments of trimmed knot spans are replaced by the extensions of the polynomial segments of the closest non-trimmed knot span that contains stable B-splines only. These extensions together with the polynomial segments of the non-trimmed knot spans form the extended B-spline basis. The final step is to represent the extended B-splines by a linear combination of the original B-splines. An extended B-spline is defined by

122 $$\begin{aligned} B^e_{i,p} = B_{i,p} + \sum _{j\in {\mathbb {J}}_{i}} e_{i,j} B_{j,p}, \end{aligned}$$

where $B_{i,p}$ is the stable B-spline that provides the extension and ${\mathbb {J}}_{i}$ is the index-set of all degenerate B-splines related to the current $B^e_{i,p}.$ The extrapolation weights $e_{i,j}$ can be determined by solving an interpolation problem. To be precise, the given polynomial function *f* of the extension over the trimmed knot span shall be represented by means of the basis functions $B_{j,p}.$ The coefficient $e_{i,i}$ is trivial since $B^e_{i,p}$ must be equal to $B_{i,p}$ within the non-trimmed knot spans, thus $e_{i,i}=1.$

Spline interpolation as described in Sect. 2.3 is not optimal to compute $e_{i,j}$ because the Greville abscissae of $B_{j,p}$ are not located within the trimmed knot span in general. Hence, a *quasi interpolation* scheme is preferred which allows an explicit computation of B-spline coefficients. In particular, the so-called *de Boor–Fix* or dual functional $\lambda _{j,p}$ [34, 35] is used: for any piecewise polynomial $f \in {\mathbb {S}}_{p,\varXi },$

123 $$\begin{aligned} f&= \sum ^{J-1}_{j=0} \lambda _{j,p}(f) B_{j,p}, \end{aligned}$$

with

124 $$\begin{aligned} \lambda _{j,p}(f)&= \frac{1}{p!} \sum ^{p}_{k=0} (-1)^{k} \psi ^{\left( p-k\right) }_{j,p}(\mu _{j}) f^{\left( k\right) }(\mu _{j}), \end{aligned}$$

125 $$\begin{aligned} \psi _{j,p}(u)&= \prod _{m=1}^{p} \left( u- u_{j+m} \right) . \end{aligned}$$

The evaluation point $\mu _{j}$ can be chosen arbitrarily within $[u_{j},\, u_{j+p+1} ].$ Substituting *f* of Eq. (124) by ${\mathcal {B}}^{s}_{i}$ yields the extrapolation weights

126 $$\begin{aligned} e_{{i},{j}}&= \frac{1}{p!} \sum ^{p}_{k=0} (-1)^{k} \psi ^{\left( p-k\right) }_{j,p}(\mu _{j}) {\mathcal {B}}^{s^{\left( k\right) }}_{i}(\mu _{j}). \end{aligned}$$

When the polynomials $\psi _{j,p}$ and ${\mathcal {B}}^{s}_{i}$ are expressed in power basis form

127 $$\begin{aligned} \psi _{j,p}(u) = \sum ^{p}_{k=0} \beta _{k} u^{k}&&\,\text {and}\,&&{\mathcal {B}}^{s}_{i}(u) = \sum ^{p}_{k=0} {\alpha }_{k} u^{k}, \end{aligned}$$

expression (126) simplifies to

128 $$\begin{aligned} e_{{i},{j}}&= \frac{1}{p!} \sum ^{p}_{k=0} (-1)^{k} \left( p- k\right) ! \beta _{p-k} k! {\alpha }_{k}. \end{aligned}$$

The interested reader is referred to [202] for details on the conversion to power basis form and further details regarding the evaluation of the dual functional. In case of a uniform knot vector, a simplified formula can be derived which solely relies on the indices of the B-splines involved, see e.g., [124].

**Fig. 65:**
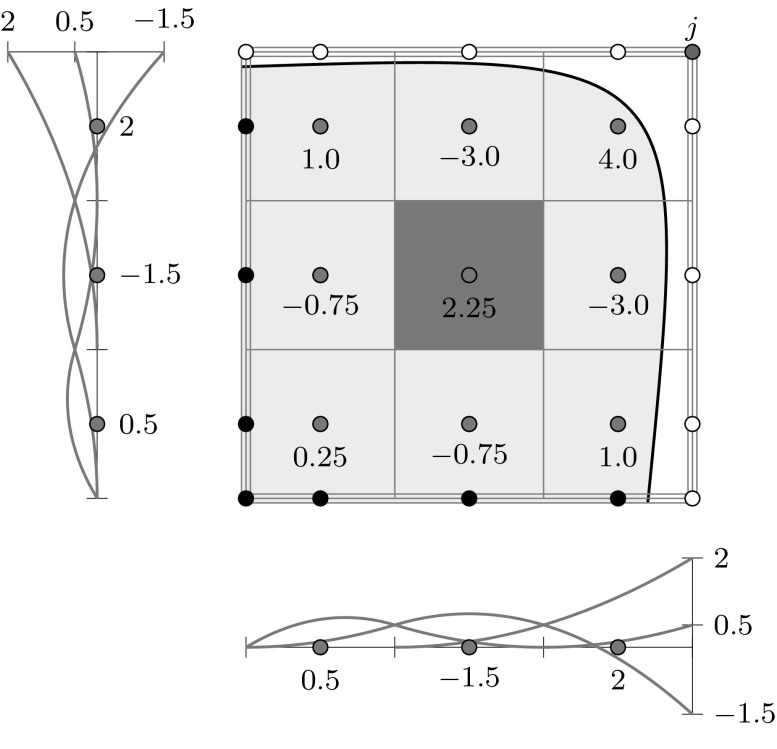
The construction of bivariate extrapolation weights $e_{{i},{j}}$ for a biquadratic basis. Stable B-splines are marked by *black* and *green circles*. The shown values of $e_{{i},{j}}$ are related to the degenerate basis function marked by the *blue circle* in the upper right corner of the parameter space. B-splines of the closest non-trimmed knot span are indicated by *green circles*. (Color figure online)

Bivariate extrapolation weights are simply obtained by the tensor product of their univariate counterparts calculated for each parametric direction as illustrated in Fig. 65. Note that the degenerate B-spline is distributed to $(p+1)(q+1)$ stable ones.

### Properties of Extended B-splines

**Fig. 66:**
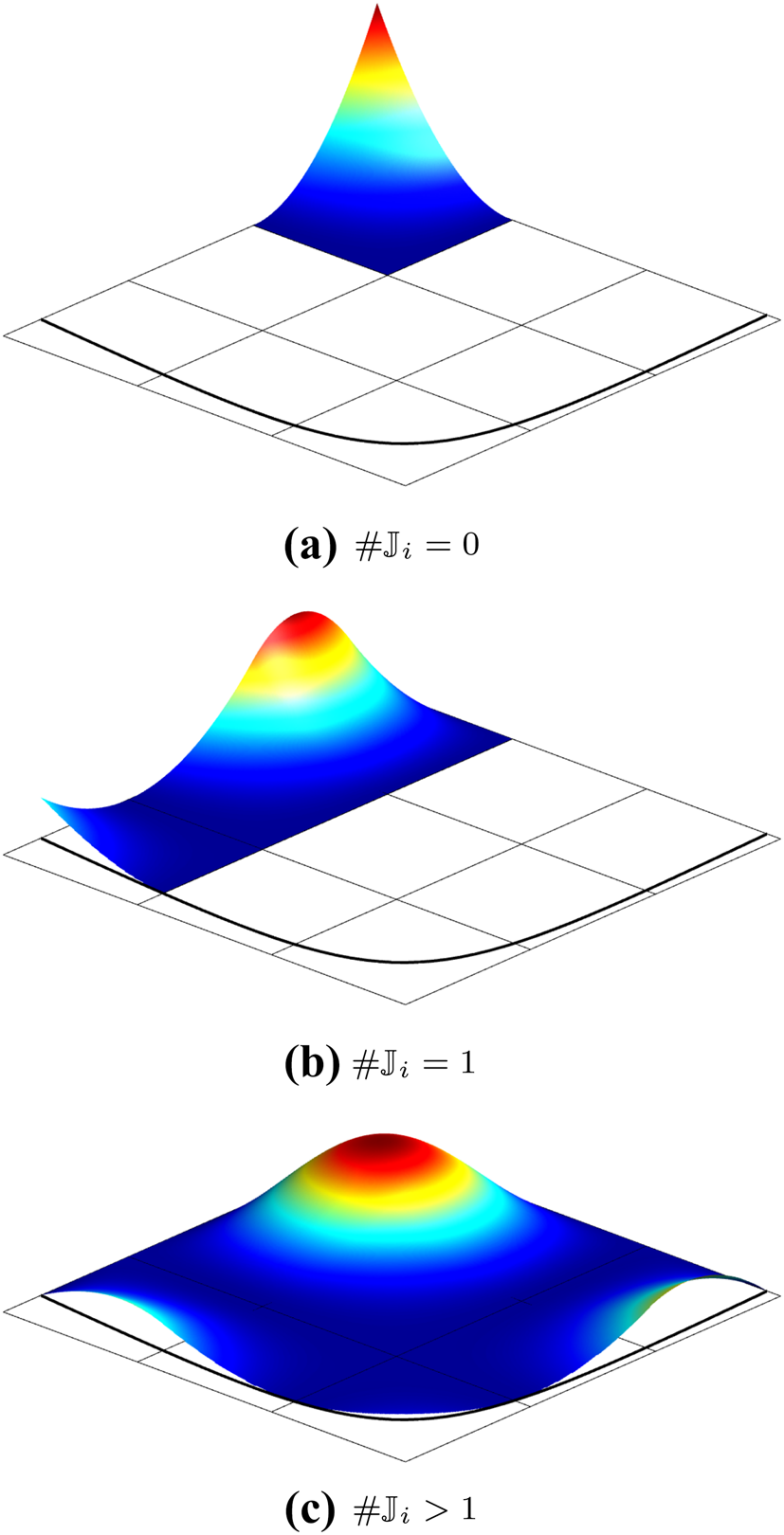
Bivariate extended B-splines $B^e_{i,p}$ with various cardinalities of the index-set ${\mathbb {J}}_{i}$ which indicates the number of related degenerate B-splines. Note that **a** is in fact a conventional B-spline, i.e., $B^e_{i,p} \equiv B_{i,p},$ since ${\mathbb {J}}_{i}$ is empty

Extended B-splines inherit most essential properties of conventional B-splines [124, 125, 127]. They are linearly independent and polynomial precision is guaranteed. Thus, they form a basis for a spline space. Each knot span has exactly $p+ 1$ non-vanishing basis functions which span the space of all polynomials of degree $\leqslant p$ over ${\mathcal {A}}^{\text{v}}.$ Furthermore, approximation estimates have the same convergence order as conventional B-splines. Extended B-splines have local support in the sense that only B-splines near the trimming curve are subjected to the extension procedure. The actual size of the affected region depends on the fineness of the parameter space, the degree of its basis functions, and the number of degenerate $B_{j,p}$ related to the stable $B_{i,p}.$ The latter is given by the cardinality of the corresponding index-set $\# {\mathbb {J}}_{i}.$ Figure 66 illustrates various examples of extended B-splines. The basis function shown in Fig. 66a is in fact a conventional B-spline since it is far away from the trimming curve.

However, there are also some differences. It is important to note that the extrapolation weights may be *negative*, hence the evaluation of extended B-splines may lead to negative values. Conventional B-splines, on the other hand, are strictly non-negative. This property is exploited in some contact formulations [295] and structural optimization [210], for instance. In such cases, the application of extended B-splines requires further considerations. The main difference in favor of extended B-splines is the *stability* of the corresponding basis. The condition number of a system is independent of the location of the trimming curve due to the substitution of B-splines with small support. Another benefit is that all Greville abscissae are located within ${\mathcal {A}}^{\text{v}}$ by construction.

### Assembling

Extended B-splines can be applied to an analysis in a very convenient manner. Suppose we have a linear system of *n* equations, one for each stable B-spline, set up by all basis functions *m* which are at least partially inside ${\mathcal {A}}^{\text{v}}.$ This yields

129 $$\begin{aligned} {\mathbf {K}} {\mathbf {u}}&= {\mathbf {f}}&&\,\text {where}\,&&{\mathbf {u}},\,{\mathbf {f}} \in {{\mathbb {R}}}^{n}&\,\text {and}\,&&{\mathbf {K}} \in {{\mathbb {R}}}^{ n \times m }, \end{aligned}$$

with $m>n.$ Upon this point, only conventional B-splines have been used to compute the system matrix ${\mathbf {K}}.$ In other words, ${\mathbf {K}}$ is set up as usual but equations related to degenerate B-splines are neglected. In order to obtain a square matrix an *extension matrix* ${\mathbf {E}} \in {{\mathbb {R}}}^{ m \times n }$ is introduced [124]. This sparse matrix ${\mathbf {E}}$ contains all extrapolation weights $e_{{i},{j}}$ including the trivial ones, i.e., $e_{{i},{i}} = 1.$ The transformation of the original to the stable extended B-spline basis is performed by multiplying the extension matrix to the system matrix. The resulting stable system

130 $$\begin{aligned} {{\mathbf {K}}_{e}} {{\mathbf {u}}}&= {\mathbf {f}}&\,\text {with}\,&&{{\mathbf {K}}_{e}} = {\mathbf {K}} {\mathbf {E}},&&{{\mathbf {K}}_{e}}\in {{\mathbb {R}}}^{ n \times n }, \end{aligned}$$

is subsequently solved and the obtained solution ${\mathbf {u}}$ corresponds to the extended B-splines of the unknown field. In case of multi-patch geometries, the extrapolation weights $e_{{i},{j}}$ of each patch have to be assembled to ${\mathbf {E}}$ with respect to the global degrees of freedom. The application of the extension operator is particularly convenient, if extended B-splines are added to an existing code.

### Application to NURBS

The stabilization described is tailored to B-spline functions where it is exploited that the extensions of any polynomial segment ${\mathcal {B}}^{s}_{i}$ can be exactly represented by a linear combination of basis functions of the trimmed knot span. In case of NURBS this property is not guaranteed due to the local influence of the weights. In order to apply extended B-splines to a trimmed NURBS basis, two different approaches may be used: (i) conversion of the CAGD model to a B-spline representation or (ii) application of an independent field approximation [199–201]. The benefit of the latter is that it allows performing the analysis based on the original NURBS model without any geometrical approximations. The key idea of independent field approximation is to use different basis functions for the representation of the geometry and the approximations of the physical fields. Hence, conventional B-splines can be used for the discretization of the field variables over NURBS patches. This allows the straightforward application of extended B-splines. In addition, the combination of NURBS for the geometry description and B-splines for the approximations has been shown to be more efficient [199] and does not lead to a loss of accuracy [184, 201, 299]. There is one caveat: independent fields are inconsistent with the isoparametric concept in mechanics and can upset the precise representation of constant strain states and rigid body motions [139].

### Assessment of Stability

**Fig. 67:**
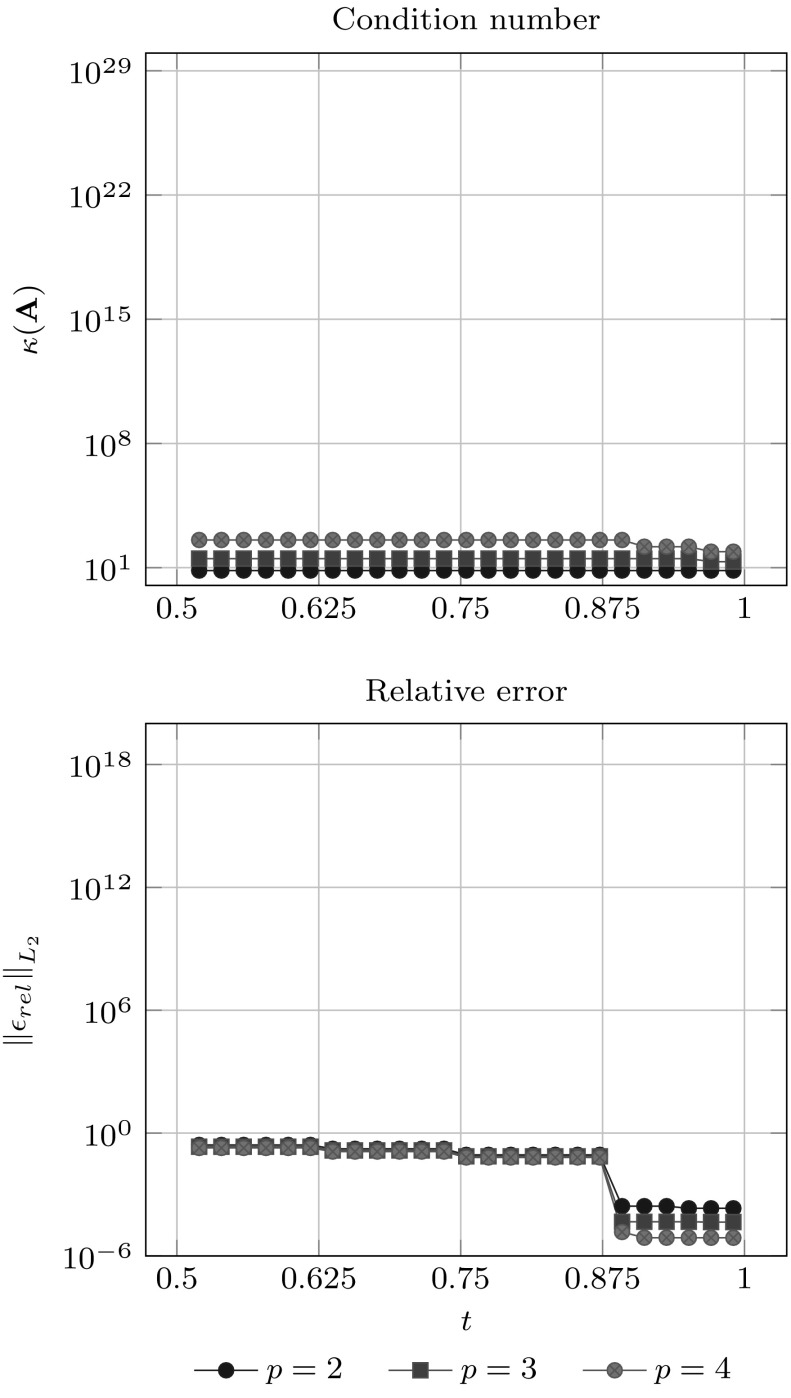
Spline interpolation problem with extended B-splines for several degrees $p.$ The condition number $\kappa ({\mathbf {A}})$ and the relative interpolation error $\left\Vert \epsilon _{rel} \right\Vert_{L_{2}}$ are related to the trimming parameter $t.$ The labels of the *horizontal axis* indicate knots of the trimmed basis

In order to assess the approximation quality and stability of extended B-splines the same interpolation problem as in Sect. 5.3.4 is considered. Again, the relative interpolation error measured in the $L_{2}$-norm $\left\Vert \epsilon _{rel} \right\Vert_{L_{2}}$ and the condition number of the spline collocation matrix $\kappa ({\mathbf {A}})$ are examined. The results are summarized in Fig. 67.

Comparing Figs. 62 and  67 shows the significant improvement of extended B-splines. If extended B-splines are used, $\kappa ({\mathbf {A}})$ hardly changes and is independent of the trimming parameter $t.$ In other words, the extended B-spline basis is stable. Consequently, the approximation quality is significantly improved. In Fig. 67, the reduction of the approximation accuracy occurs only due to the reduction of the degrees of freedom $n,$ i.e., number of extended B-spline, as the trimming parameter $t\rightarrow 0.5.$

### Summary and Discussion

The concept of extended B-splines substitutes unstable basis functions by extensions of stable ones. It is established in a very flexible manner and requires only the presence of a sufficient number of stable basis functions. In general, this requirement is non-restrictive and can be fulfilled by refinement of the basis. Still, it may be an issue if the design object contains very small fillets. Only B-splines close to the trimming curve are affected by the stabilization procedure. The number of B-splines depends on the distance of the trimming curve to the knot span which provides the stable B-splines. This correlates with the degree $p$ of the basis function since the size of its support extends over $p+1$ knot spans.

## Final Remarks and Conclusions

The present work accumulates several topics related to the treatment of trimmed models and the interoperability problem between CAD and downstream applications in general.

It is apparent that trimming is a fundamental technique for geometric design. Most importantly, it enables the computation of intersections between free-form surfaces. However, intersection curves cannot be determined exactly, which leads to various problems. As a result, an intersection is usually approximated by several independent curves, one in model space and one in the parameter space of each surface involved. Their images in model space do not coincide and there is no link between these curves. The resulting gaps and overlaps between the surfaces yield robustness issues due to a lack of exact topological consistency. These problems are still unresolved, despite the fact that they have been the focus of an enormous amount of research.

Since the robustness issues of trimmed models are particularly crucial for downstream applications, the exchange of CAD data is examined as well. Neutral exchange standards seem to be the most comprehensive strategy, but it is important to note that all translations lead to loss of information. Moreover, the capabilities of the various exchange formats are *not* equivalent. It is demonstrated that STEP is superior to IGES. STEP should be preferred in general and especially if the topology of a model needs to be extracted.

In the context of analysis, the current approaches can be divided into two different philosophies. On the one hand, global approaches aim to fix the problems of trimmed models before the simulation by a reconstruction of the geometric representation. Using local approaches, on the other hand, trimmed models are directly employed, but the analysis has to be enhanced in order to deal with all the flaws of the geometric representation. It may be argued that the former addresses the issue from a CAD point of view, whereas the latter utilizes an analysis perspective. The fact that the problem can be tackled by these diverse directions emphasizes the central role of trimmed models for the integration of design and analysis.

The main conclusions of the present review can be summarized as follows:

- Trimming seems to be a simple and benign procedure at first glance, but its consequences are profound.
- Robustness issues are the price for the flexibility of trimmed models.
- Flaws of trimmed models are usually hidden from the user, but surface as soon as they are applied to a downstream application.
- To overcome these issues is a crucial aspect regarding the integration of design and analysis.
- The success of CAD data exchange depends on the quality of the design model *and* the capability of the exchange format.
- There is no canonical way to deal with trimmed models, neither in analysis nor in design, at least so far.

It is hoped that this review provides a helpful introduction to the topic and an impetus for further research activities. There are indeed several open issues worth exploring: optimization of the reparameterization of the global approach based on isocurves, assessment of the affect of gaps on the analysis in case of local schemes, and application to isogeometric collocation, just to name a few. In general, the step from academic examples to practical multipatch models is perhaps the most challenging task. Robust algorithms should be able to take the tolerances of a design model into account. On the other hand, it may be an unrealistic aim to find a solution that can deal with every possible trimmed geometry. Similar to the quality of conventional meshes, an isogeometric simulation is effected by the quality of the design model. Hence, the specification of distinct properties that classify a design model to be analysis-suitable are needed so that a designer can get a direct feedback if a model requires an improvement—providing the right information to the right person at the right time. We close this review by emphasizing that a holistic treatment of the engineering design process requires the aligned efforts of both the design and the analysis communities.

## Appendix: Exchange Data File Examples

The source files of the neutral exchange format example presented in Sect. 4.2.3 are given in this section. The models have been constructed with the commercial CAD software Rhinoceros 5.

It should be pointed out that the IGES examples, i.e., Files 1 and 2, provide the *same* information although the topological data of the two models was different before the extraction procedure. In particular, these files differ only in the representation of some floating point values, e.g., 0.0D0 and 8.881D-16, and the sequence of the numbering of a few parametric data entities, e.g., 0000029P of File 1 is equal to 0000037P of File 2. The corresponding STEP examples with the correct topology data are given in Files 3 and 4.

The interested reader is referred to the homepage of STEP Tools, Inc. 21 for further examples of STEP files covering various application protocols.

## Footnotes

1 The model space is also referred to as physical space.

2 The genus *g* of a plane algebraic curve is specified by the degree $p$ of the curve and the number $I$ and multiplicity $m$ of its singular points: $g=\frac{1}{2}\left( {p^{2}}-3p+2-{\sum _{i=1}^{I}} {m_{i}} ({m_{i}-1})\right) .$

3 Marching methods are also referred to as tracing methods.

4 A multi-affine and totally symmetric mapping is called a blossom (or polar form). Blossoms can be used to define spline algorithms in an elegant way. For details see [236].

5 B-Rep models with free-form surfaces are also referred to as sculptured models.

6 Stereolithography is an early and widely used 3D printing technique.

7 Clough–Tocher is a splitting scheme to construct ${C^{1}}$-continuous splines over triangulations.

8 Regular internal vertices have four incident edges, also referred to as valency $k=4.$ All other settings, i.e., $k\ne 4,$ are denoted as extraordinary vertices.

9 Bjarne Stroustrup is the inventor of the programming language C++.

10 The word “interim” was used in the first draft.

11
http://www.iso.org, September 2016.

12
https://www.pdesinc.org/ResourceIndex.html, September 2016.

13
http://www.steptools.com/library/standard/, September 2016

14
http://www.steptools.com/sc4/archive/, September 2016.

15 There exist also a compressed format for details see [155].

16 There are various names for fictitious domain methods such as embedded domain methods, finite cell methods, WEB-spline methods, and immersed boundary methods. The principle idea, however, is the same.

17 Usually, the jump term coefficient depends on the geometric angle of the boundary at the point considered, see e.g., [96].

18 A diffeomorphism is a ${C^{\infty }}$ mapping with a ${C^{\infty }}$ inverse.

19 Regions related to a $\gamma ^{u}$-portion are treated in an analogous manner.

20 To be precise, it is suggested to use the control points of the intersection curves in the model space and to apply point inversion to determine their location in the parameter space. However, there is no particular reason why the control polygon of the trimming curve cannot be used directly.
